# Amphibians and Reptiles Exhibit Different Ecological and Evolutionary Spatial Patterns in the Amazon Basin

**DOI:** 10.1002/ece3.70677

**Published:** 2025-03-19

**Authors:** Jhon Jairo López‐Rojas, Diego Henrique Santiago, Mirco Solé, Ricardo Lourenço‐de‐Moraes

**Affiliations:** ^1^ Programa de Pós‐graduação em Zoologia, Departamento de Ciências Biológicas Universidade Estadual de Santa Cruz, Rodovia Jorge Amado Ilhéus Bahia Brazil; ^2^ Facultad de Ecología Universidad Nacional de San Martín Moyobamba Peru; ^3^ Instituto Federal do Paraná, PPG Sustentabilidade, Campus Umuarama Paraná Brazil; ^4^ Museum Koenig Bonn Leibniz Institute for the Analysis of Biodiversity Change Bonn Germany; ^5^ Departamento de Engenharia e Meio Ambiente Universidade Federal da Paraíba Rio Tinto Paraíba Brazil

**Keywords:** Amazon, diversity metrics, ecoregion, functional, phylogenetic, taxonomic

## Abstract

Understanding spatial variability in ecological and evolutionary patterns is key to Amazonian biodiversity conservation. This study examined taxonomic, phylogenetic, and functional diversity across amphibians and reptiles, assessing the influence of elevation, interrelationships among metrics, and distribution across five Amazon Basin ecoregions, exploring the “cradle” (speciation) and “museum” (lineage preservation) hypotheses. We analyzed 1011 amphibian species from three lineages and 828 reptile species from four lineages. Integrating distribution maps, phylogenies, and trait data, we calculated phylogenetic (PD), functional (FD), and taxonomic (TD) diversity, including mean phylogenetic (PD_mntd_) and functional (FD_mntd_) distance to the nearest taxon. We examined spatial regressions between diversity metrics and elevation, assessed correlations among metrics, and compared diversity metrics across ecoregions for each lineage. Diversity metrics across amphibian and reptile lineages reveal distinct geographical patterns related to elevation. Anurans exhibit higher PD, FD, and TD in the western Amazon, while squamates show hotspots at low altitudes. Testudines are linked to major rivers, and crocodilians display high PD near the equator. Anurans and squamates show elevated PD_mntd_ and FD_mntd_ in the Andes, whereas testudines are found in cratonic regions. Significant correlations and notable differences among ecoregions were found, especially in the Andes and low regions of the Amazon Basin. This study highlights the diverse eco‐evolutionary patterns among amphibian and reptile lineages in the Amazon Basin, each exhibiting distinct hotspots distributed across ecoregions. The findings align with the cradle‐museum hypothesis, suggesting that some regions serve as centers of ongoing diversification, others preserve ancient lineages, or serve as both. The cradle‐museum hypothesis should be carefully analyzed, as each taxon presents a distinct pattern. This research underscores the necessity for targeted conservation strategies tailored to distinct ecological and evolutionary dynamics across ecoregions.

## Graphical abstract

This study investigates the spatial variability of ecological and evolutionary patterns in amphibian and reptile lineages within the Amazon Basin. Our findings highlight distinct diversity patterns across lineages and ecoregions.

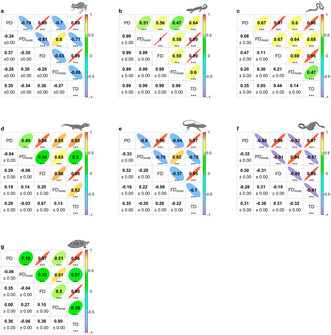

## 1 Introduction

Over millions of years, the Earth experienced a great number of stochastic events (Raup 1986). During the Cenozoic Era, the Amazon region underwent important landscape changes due to geomorphogenetic processes (Val et al. 2021). The formation of the Andes, the development of a vast mega wetland system that once covered much of western Amazonia, the emergence of the Amazon rivers, and the separation of the proto‐Amazon in the east (across the Brazilian and Guiana Shields) all played a critical role in shaping the spatial patterns of biodiversity (Guayasamin et al. 2021; Zapata‐Ríos et al. 2021; Val et al. 2021). In this ecological and evolutionary context, the “cradles and museums” hypothesis has been proposed (Stebbins 1974; Jablonski 1993; Chown and Gaston 2000). According to this hypothesis, certain geographical areas act as “cradles” of speciation, while other regions function as “museums,” preserving ancient evolutionary lineages (Stebbins 1974; Jablonski 1993; Chown and Gaston 2000). This hypothesis also suggests that while some areas act as museums, preserving existing traits and ecological functions, other areas serve as cradles, promoting the generation and maintenance of functional characteristics (e.g., body size, feeding, and reproductive strategies) and ecological functions (e.g., predation, seed dispersal, and nutrient cycling) (Freitas et al. 2021; Ochoa‐Ochoa et al. 2020).

Multiple metrics of diversity have been proposed and implemented to capture the spatial patterns of ecological and evolutionary aspects of biodiversity (Tucker et al. 2017; Mammola et al. 2021; Lourenço‐de‐Moraes et al. 2023) and have been studied at multiple spatial or temporal scales of various taxonomic groups (Coronado et al. 2015; Ochoa‐Ochoa et al. 2020; Freitas et al. 2021). These metrics of diversity include Taxonomic diversity (TD), Phylogenetic diversity (PD), and Functional diversity (FD). Taxonomic diversity is a widely recognized metric for assessing biological diversity, providing a quantifiable measure of the number of species in a given area (e.g., Lourenço‐de‐Moraes et al. 2021). PD measures the length of the branches of a phylogenetic tree encompassing all the species present in a community (Faith 1992). It is a measure that takes into account the depth of phylogenetic divisions (i.e., how long ago species shared a common ancestor) (Faith 1992). Regions with high PD may contain different levels of evolutionary history, ranging from more recent to more basal species (Davies and Buckley 2011; Faith 2016).

Functional diversity (FD) considers the ecological functions performed by species in a community, providing a better understanding of how functional traits vary over space in a biogeographical context (Magurran 2004; Mammola et al. 2021). It assumes that closely related species have similar functions and may overlap more in their ecological niche, leading to sympatry and divergent trait evolution (Gerhold et al. 2015). Functional traits are used to estimate functional diversity, quantify the diversity and distribution of ecological strategies, and help identify factors in the structure of a community, such as abiotic conditions or dispersal filters (Rosenfeld 2002; Mason et al. 2013). Ecological, biogeographical, and evolutionary forces may either expand or constrain trait space, thereby driving functional divergence (Tucker et al. 2017; Mammola et al. 2021). Another index used to interpret these divergences is the mean nearest taxon distance (MNTD) (Webb 2000). The MNTD calculates the mean distance between each species in a community and its nearest identical species in the phylogenetic Tree and is therefore influenced by recent speciation events (Mazel et al. 2016). Both phylogenetic and functional diversity can be measured using the MNTD (Montaño‐Centellas, Loiselle, and Tingley 2021). Given the increasing threats from habitat loss and climate change in the Amazon (Nobre et al. 2020), understanding the spatial patterns of biodiversity is crucial for guiding conservation efforts. Identifying areas of high phylogenetic and functional diversity can inform strategies to protect not only species but also their evolutionary potential and ecological roles.

Our understanding of taxonomic (TD), functional (FD), and phylogenetic (PD) diversity patterns in Amazonian amphibian and reptile groups remains limited. To investigate the drivers of these lineages' spatial distribution, we first describe the distribution patterns of TD, PD, and FD and explore how elevation influences these patterns. In high‐altitude regions, we expect “museum” areas to exhibit high PD values, preserving ancient lineages. Conversely, in regions identified as “cradles,” we anticipate higher TD and PD values due to recent speciation events. At intermediate elevations, we predict increased FD, where old and new lineages coexist, resulting in a broader range of functional traits.

We also divided the Amazon into ecoregions to examine how these metrics vary across regions according to the cradle‐museum hypothesis. In regions identified as “cradles,” we expect high TD and PD values, reflecting the predominance of recent lineages. In contrast, “museum” ecoregions are expected to have high PD, indicating the preservation of ancient lineages. Some ecoregions may even function as both “cradles” and “museums,” presenting patterns that combine ancient and recent lineages.

## 2 Materials and Methods

### 2.1 Study Area

This study focuses on the Amazon Basin, the world's largest river basin stretching from the Andes Mountains to the Atlantic Ocean, covering 5,982.303 km^2^ (Mayorga et al. 2012). This vast region spans several countries, including Brazil, Bolivia, Colombia, Ecuador, Peru, Venezuela, Guyana, Suriname, and French Guiana (Mayorga et al. 2012). The Amazon Basin's geological traits, shaped by tectonic activity and volcanic sediment deposits, influence the mineral composition of sediments in its rivers (Val et al. 2021). Its geology can be categorized into four main components: the Andes, the Amazonian floodplain, and the Guiana and Brazil‐Central shields, each contributing distinct geological traits that shape the region's biodiversity (Val et al. 2021). The Amazon Basin has a tropical forest climate with 2225 mm of average annual precipitation and a mean temperature of about 24°C, varying throughout the region (Zhong et al. 2021). It encompasses diverse habitats across various ecoregions (Olson et al. 2001), ranging from sea level to an altitude of 6540 m (Saatchi 2013).

### 2.2 Spatial Data

To define the area of interest, the Amazon Basin, we generated a mask using the *amzbasin.tif* file obtained from Mayorga et al. (2012). This mask was then used to create, clip, and geoprocess the grid system, the metrics for each lineage, and the reclassification of ecoregions.

Spatial records of species distribution were gathered from diverse origins. Amphibian and crocodilian data were extracted from the IUCN database version 2023.1 (2024). Data regarding Squamata, encompassing lizards and snakes, were sourced from Roll et al. (2017), while details about testudines were acquired from Rhodin et al. (2021). This new version is more detailed and has more defined polygons, excluding areas where the species may not occur. Our analysis included a total of 1839 species (see Appendix S1, Table S1.1, in Supporting Information), comprising 1011 amphibians (979 Anura, 27 Gymnophiona, and five Caudata) and 828 reptiles (394 snakes, 403 lizards, 24 Testudines, and seven crocodilians). We used georeferenced point observations of herpetofauna recorded during field expeditions between 2008 and 2022 in various locations of the Amazon Basin (Appendix S1, Figure S1.1) solely to verify the validity of the current distribution maps for each taxonomic group. After confirming that all occurrences fell within the established distribution ranges, we proceeded with the analysis using only these distribution maps. To map the species distribution of each lineage, a presence‐absence matrix was generated on a grid comprising 49,940 cells, each spanning 0.1° latitude and longitude, employing ArcGIS Pro (Esri 2021). To ensure the accuracy of our analysis, all species were confirmed with the latest taxonomy based on current publications and credible records for amphibians and reptiles, following Frost (2024) and Uetz, Freed, and Hošek (2023), respectively.

### 2.3 Functional Diversity

To map functional diversity (FD), we compiled a database of 6–8 functional trait categories, encompassing morphology, life history, and behavioral characteristics (e.g., Ferreira et al. 2019; Nunes‐de‐Almeida, Haddad, and Toledo 2021; Lourenço‐de‐Moraes et al. 2023; Macip‐Ríos, Butterfield, and Raya‐García 2023). These traits were derived from original studies and fieldwork observations conducted between 2008 and 2022 (refer to Appendix S2, Table S2.1–S2.5). For most functional traits, a species may have multiple associated values, allowing a species to exhibit various characteristics within the same trait without these values being mutually exclusive.

For amphibians, six functional traits were considered (see Table S2.1): (1) maximum snout‐vent length (mm) or tail (for Caudata); (2) activity (diurnal and nocturnal); (3) habit (terrestrial, arboreal, aquatic, semi‐aquatic, phytotelmate, fossorial, rheophilic, and cryptozoic); (4) habitat (closed: closed forests, primary or secondary; open: open areas, punas, paramos, shrublands, natural pastures, crops, and urban areas; semi‐open: areas with partial vegetation, forest transition, and open areas, forest plantations, savannas, poorly vegetated areas, elfin forest, semi‐arid forest, and dry forests; floodplain: *varzeas*, *igapos, aguajales, restingas*, and swamp forest); (5) nine Antipredator mechanisms, modified from Ferreira et al. (2019): toxic, non‐toxic, unpalatable or bad odor, camouflage, immobility, interrupt call, aposematism, charge, posture, escape, warming sound, cloacal discharge, secretion, aggression, and distress call; (6) 39 reproductive modes following Nunes‐de‐Almeida, Haddad, and Toledo (2021). Each trait was assessed separately for each taxon (Anuran, Caudata, and Gymnophiona).

We also assessed specific functional traits for each reptile lineage. Crocodilians (Table S2.2): (1) body size (largest male size and female size at maturity); (2) Reproduction (nest type, relative clutch mass); (3) tolerance to extreme climates (aestivation and brumation); (4) potential to act as ecosystem engineers (ability to dig burrows); (5) habitat type (generality, salt tolerance, and terrestriality); (6) diet/foraging strategy (diet generality, skull shape, and bite force); (7) activity (day and night).

Lizards (Table S2.3): (1) activity (diurnal and nocturnal); (2) body size (mm): small (< 100 mm), medium (100–250 mm), and large (> 250 mm); (3) tail (%–proportional to body size): short (< 50%), medium (50%–100%), and large (> 100%); (4) reproduction (oviparous and viviparous); (5) habitat (closed, open, and semi‐open); (6) habit (terrestrial, saxicolous, arboreal, aquatic, cryptic, bromelicolous, and fossorial); (7) prey (insectivores, crustaceans, mollusks, worms, piscivores, birds, reptiles, amphibians, fungi, eggs, mammals, fruit, and plants).

Snakes (Table S2.4): (1) activity (diurnal and nocturnal); (2) total length (mm): small (< 500 mm), medium (500–1000 mm), and large (> 1000 mm); (3) tail (%–proportional to large): short (< 15%), medium (15%–30%), and large (> 30%); (4) reproduction (oviparous and viviparous); (5) teeth (aglyphous, opisthoglyphous, proteroglyphous, and solenoglyphous); (6) habitat (closed and open); (7) habit (terrestrial, arboreal, aquatic, and fossorial); and (8) prey (mammals, birds, lizards, snakes, frogs, fishes, mollusks, earthworms, eggs, arthropods, caecilians, and amphisbaenians).

Testudines (Table S2.5): (1) carapace length (mm); (2) shape of the shell following (Macip‐Ríos, Butterfield, and Raya‐García 2023): spherical shell, flatter shell, and surface area‐to‐volume ratio of the carapace (SA/V); (3) aestivation status (absent and present); (4) habit (terrestrial and semi‐aquatic); (5) activity (diurnal and nocturnal); (6) prey (fishes, annelids, arthropods, mollusks, mammals, amphibians, birds, aquatic plants, and plant/seeds terrestrial); (7) environment (swamp/lake, forest floor, stream, and river); and (8) habitat (closed and open).

### 2.4 Phylogenetic Diversity

We obtained branch lengths from three time‐calibrated phylogenies: one comprising 7238 extant amphibian species (Jetz and Pyron 2018; Portik, Streicher, and Wiens 2023), another encompassing 9755 squamates (Tonini et al. 2016), and the third involving crocodilians and testudines (Colston et al. 2020). For each phylogeny, we randomly resolved these tree polytomies 1000 times on non‐zero length branches using the “fix.poly” commands with the “RRphylo” package (Castiglione et al. 2018). Phylogenetic diversity metrics were calculated for 958 amphibian species, 734 squamate reptiles, seven crocodilians, and 19 testudines.

We calculated Faith's PD to measure the PD of each lineage, which summarizes the total length of phylogenetic branches of all species in a community, being especially sensitive to rare and basal species (Faith 1992). Furthermore, dendrograms are employed to calculate functional diversity (FD), which represents the trait space of a community (e.g., Andrew et al. 2024; Poch et al. 2024). This allows for the measurement of total functional richness by adding the lengths of the branches of the tree constructed for the species grouped in said space (Petchey and Gaston 2006; Mammola et al. 2021). A functional dendrogram of each lineage was created using a dissimilarity matrix of its traits using the “hclust” function with the “average” method of the “ade4” package (Thioulouse et al. 2018), this matrix was converted into a tree object of class phyllo with the “as.phylo” function of the “ape” package (Paradis and Schliep 2019). This graphical representation facilitates the visualization of functional relationships between species and is closely linked to tree‐based phylogenetic diversity, providing a coherent framework for comparing different metrics of diversity (phylogenetic and functional) (Mammola et al. 2021). Using the respective functional and phylogenetic dendrograms, we calculated TD, FD, and PD for each grid cell using the “pd” function of the R package “picante” (Kembel et al. 2010).

### 2.5 Phylogenetic and Functional Diversity With MNTD

Mean Nearest Taxon Distance (MNTD) is a metric that uses the phylogenetic distance matrix to quantify the average distance of the minimum relationship values between pairs of species in a community (i.e., the average distance that separates each species in a community from its closest relative, see Webb 2000). MNTD is a terminal metric that weights relationships at the extremes of the phylogeny (e.g., species within the same genus) (Webb 2000).

While we chose to use Mean Nearest Taxon Distance (MNTD) to measure phylogenetic diversity in this study, this decision was driven by our interest in capturing recent speciation events and focusing on the terminal branches of the phylogeny, which MNTD is particularly sensitive to. This allowed us to explore how closely related species within the same community contribute to phylogenetic diversity and how this relationship may be influenced by geographical factors such as elevation. While the Mean Pairwise Distance (MPD) provides a broader view of phylogenetic relationships by considering the average distance between all species pairs in a community, we prioritized MNTD for its ability to detect terminal diversification patterns, which were central to our analysis of community structure and diversity dynamics (Webb, Ackerly, and Kembel 2002).

To assess whether PD, FD, PD_mntd_ and FD_mntd_ of each lineage were influenced by species richness, we employed the “ses.pd” function for PD and FD, and the “ses.mntd” function for PD_mntd_ and FD_mntd_. We applied “independent swap” null models (Devictor et al. 2010; Swenson 2014) of the “picante” package in R (Kembel et al. 2010), allowing us to determine whether the observed estimates for each metric significantly deviated (either lower or higher) from those expected by random chance. We conducted 1000 replicates for each metric, obtaining *p* > 0.05, indicating that the observed values were consistent with those predicted (see Appendices S3 and S4).

To visually represent the metrics across the grid of 49,940 cells, we used the raster maps with minimum and maximum values for each amphibian and reptile lineage using the “ggplot2” (Wickham 2016) and “raster” (Hijmans 2020) packages.

To explore the spatial relationships between the metrics and elevation, we utilized the Spatial Relationship Modeling Toolbox in ArcGIS Pro (Esri 2021) to calculate both Ordinary Least Squares (OLS) and Geographically Weighted Regression (GWR) models. The OLS model was used as a baseline to assess initial global patterns, while the GWR model was applied to capture local variations, adjusting for the spatial autocorrelation of the data. The GWR model employed a default fixed distance kernel and the corrected Akaike Information Criterion (AICc) as the bandwidth selection method to optimize model performance. Both models were assessed using the Akaike Information Criterion (AIC), with the model producing the lowest AIC value considered the best fit.

In addition, we performed a correlation analysis of the metrics using the correlogram from the “corrplot” package (Wei and Simko 2024) and assessed the spatial associations between metric pairs for each lineage by applying Tjøstheim's coefficient with the “cor.spatial” function from the “SpatialPack” package (Vallejos, Osorio, and Bevilacqua 2020).

### 2.6 Creating and Testing Ecoregions

The world's terrestrial ecoregions (Olson et al. 2001) were downloaded and clipped to the Amazon Basin with ArcGIS Pro (Esri 2021). The ecoregions were merged leading to their reclassification into five distinct ecoregions (see Appendix S1, Figure S1.2).

This reclassification, based on geological history, physical geography, topography, vegetation, and hydrography, follows Val et al. (2021) and includes the Andes, Western Lowland, Eastern Lowland, Guiana Shield, and Brazilian Shield. Metric values for each lineage in each ecoregion were obtained using the ArcGIS Pro (Esri 2021) Intersect tool. Subsequently, we verified that the ecological and evolutionary values of each ecoregion differed from each other. This analysis was conducted using analysis of variance (ANOVA) followed by Tukey's post hoc test. The statistical analysis employed the “anova_test” and “tukey_hsd” functions from the “rstatix” package (Kassambara 2023).

## 3 Results

### 3.1 Geographic Patterns of Diversity Metrics and Elevation Influence in Amphibians and Reptiles Lineages

**FIGURE 1:**
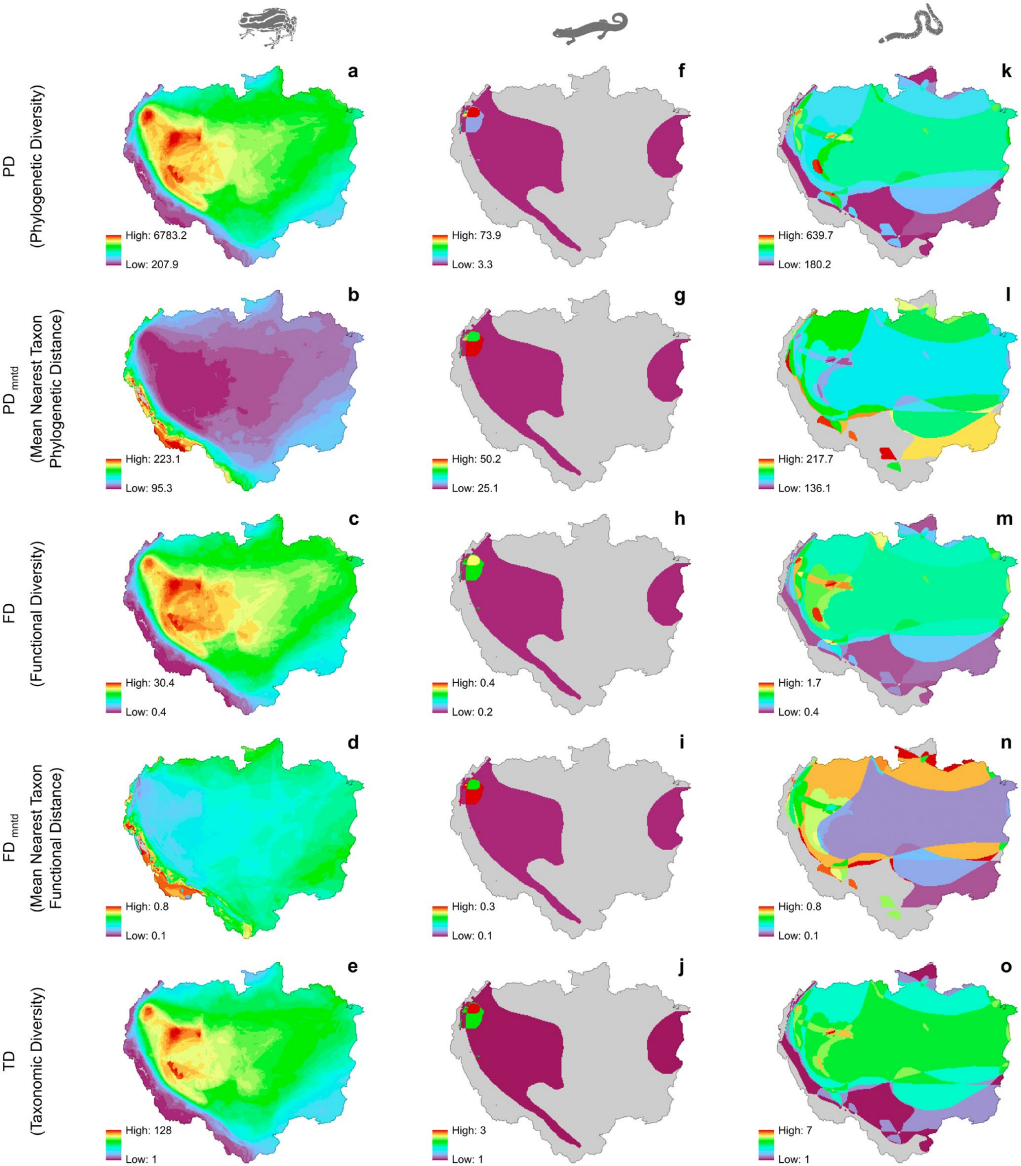
Multiple metrics of diversity distribution patterns of amphibian lineages (a–e: Anura; f–j: Caudata; k–o: Gymnophiona) in the Amazon Basin.

**FIGURE 2:**
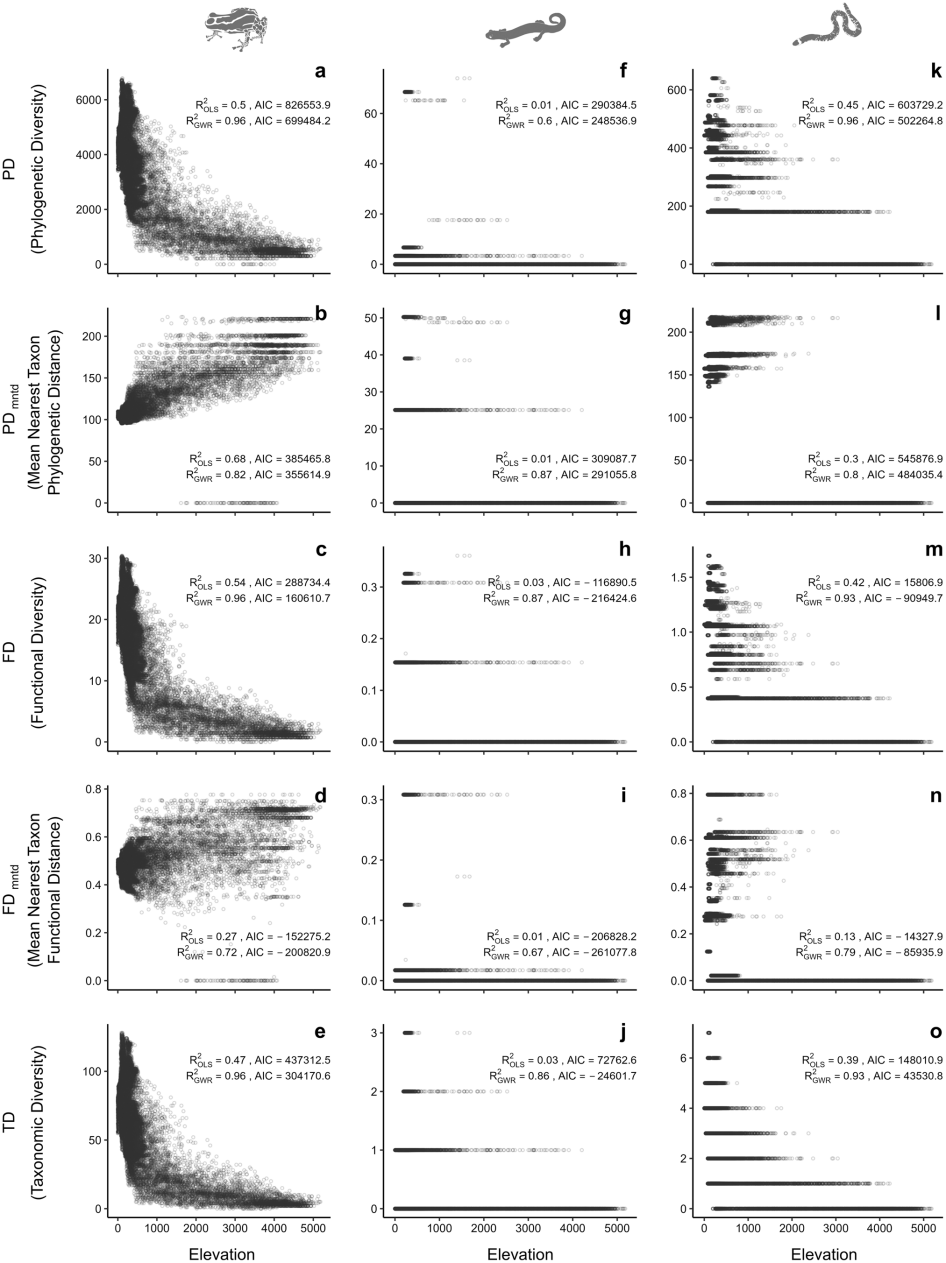
Multiple metrics of diversity distribution patterns of amphibian lineages (a–e: Anura; f–j: Caudata; k–o: Gymnophiona) along elevational gradients in the Amazon Basin.

Anurans exhibited high PD, FD and TD values in the western region of the Amazon Basin (Figure 1a,c,e). Elevation was negatively associated with FD, PD, and TD variation. The adjusted $R_{\mathrm{OLS}}^{2}$ values from the OLS model ranged from 0.47 to 0.54, indicating a moderate fit that provides a general overview of the relationship between diversity metrics and elevation without accounting for specific spatial variations. In contrast, the Geographically Weighted Regression (GWR) models revealed stronger associations, with adjusted $R_{\mathrm{GWR}}^{2}$ values reaching 0.96 (Figure 2a,c,e). This high R^2^ suggests that the relationship between elevation and diversity metrics varies significantly across regions and that the GWR model represents these local variations with greater precision. Conversely, lower and intermediate regions of the Amazon Basin exhibited low values of PD_mntd_ and FD_mntd_ (Figure 1b,d). The GWR model provided a stronger fit for these relationships, highlighting the positive influence of elevation on the variation of PD_mntd_ and FD_mntd_, with higher values observed at altitudes above 1100 and 500 m.a.s.l, respectively (Figure 2b,d).

Caudata exhibited high values for all metrics in the northwestern region of the Basin (Figure 1h,j). The GWR model demonstrated a stronger fit compared to OLS models, effectively capturing the varying levels of association between diversity metrics and elevation (Figure 2f–j). Gymnophiona exhibited high PD, FD, and TD values in the lower and intermediate regions of the Amazon Basin (Figure 1k,m,o). Elevation was negatively associated with PD, FD, and TD variation showing adjusted $R_{\mathrm{OLS}}^{2}$ values of 0.45, 0.42, and 0.39, respectively (Figure 2k,m,o). Notably, elevation explained 30% of the variation in PD_mntd_ and 13% in FD_mntd_. around the Amazon Basin. The GWR models provided a better fit for these relationships, particularly for PD and TD, which had GWR‐adjusted $R_{\mathrm{GWR}}^{2}$ values of 0.93 (Figure 2l,n).

**FIGURE 3:**
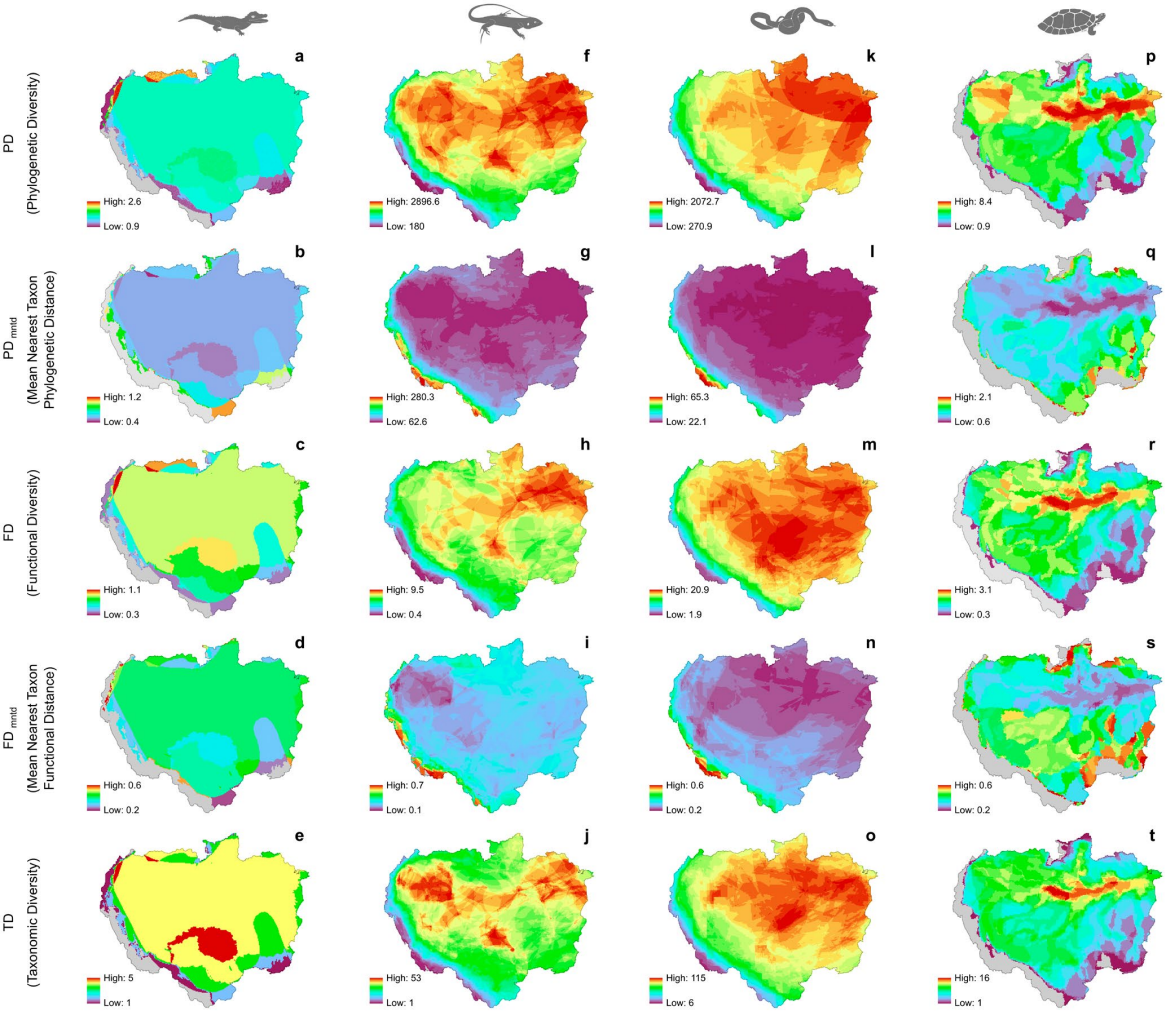
Multiple metrics of diversity distribution patterns of reptile lineages (a–e: Crocodilians; f–j: Lizards; k–o: Snakes; p–t: Testudines) in the Amazon Basin.

**FIGURE 4:**
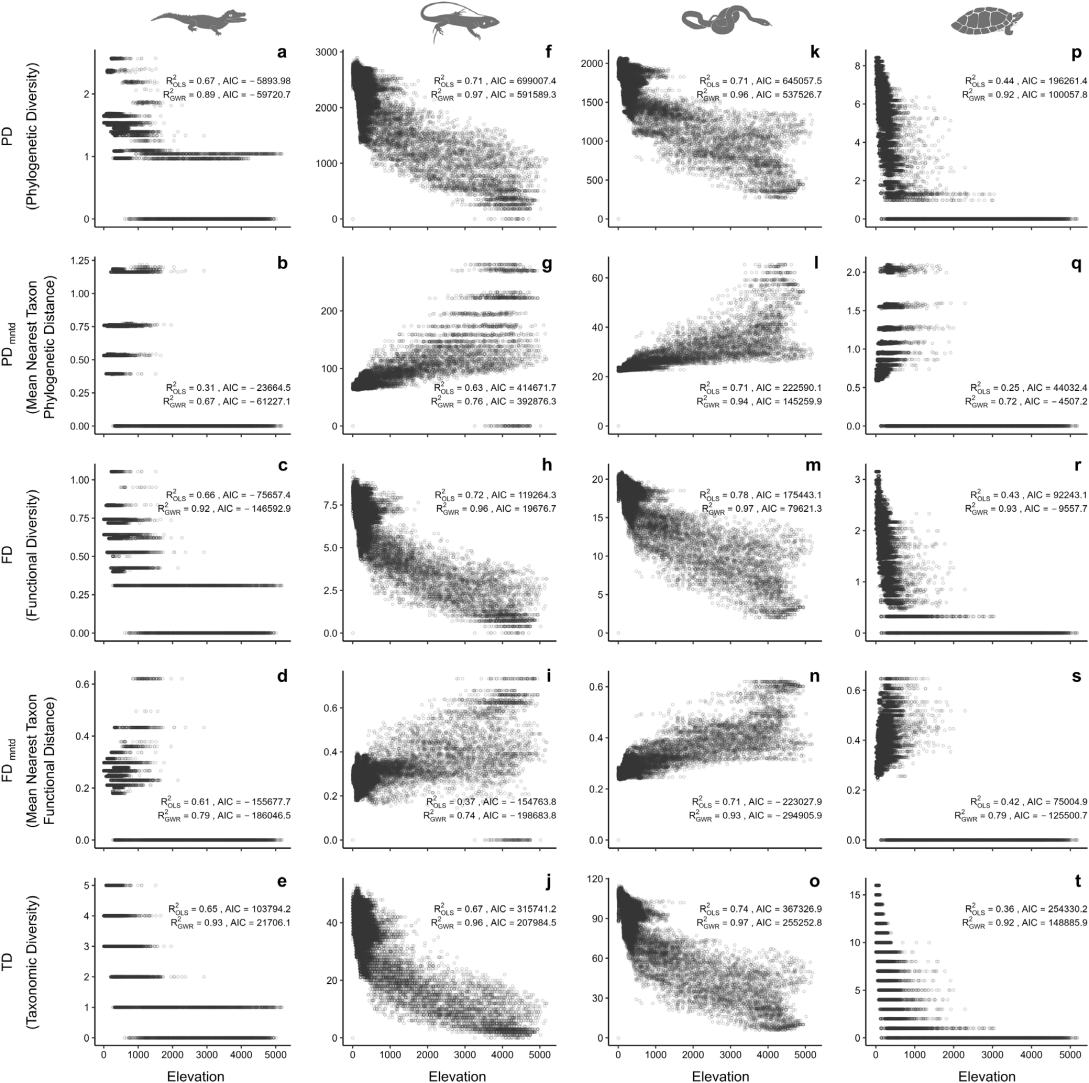
Multiple metrics of diversity distribution patterns of reptile lineages (a–e: Crocodilians; f–j: Lizards; k–o: Snakes; p–t: Testudines) along elevational gradients in the Amazon Basin.

Crocodilians presented high PD, FD, and TD values in the northwestern extreme of the Amazon Basin (Figure 3a,c,e). Elevation explained more than 60% of the variation in PD, FD, TD, and FD_mntd_. GWR models exhibited robust fits for these relationships, particularly for PD, FD, and TD, with adjusted $R_{\mathrm{GWR}}^{2}$ values of 0.89, 0.92, and 0.93, respectively (Figure 4a–e).

Lizards and snakes exhibited PD, FD and TD hotspots primarily across the Amazonian floodplain, extending from the northeastern to the central and northwestern regions (Figure 2f,h,j,k,m,o). Both lineages also showed high PD_mntd_ and FD_mntd_ values in the central Andes (Figure 2g,i,l,n).

For lizards, according to the OLS model, elevation negatively explained 72%, 71%, and 67% of the variation in FD, PD, and TD, respectively, while showing a positive association with PD_mntd_, accounting for 63% of its variation. The GWR models significantly improved the fit for these relationships, with adjusted $R_{\mathrm{GWR}}^{2}$ values reaching 0.97 for PD, and 0.96 for both FD and TD.

In snakes, elevation was a significant predictor of diversity metrics, explaining over 70% of the negative variation in PD, FD, and TD, while positively influencing PD_mntd_ and FD_mntd_ in the OLS model. GWR models further improved these fits, with adjusted $R_{\mathrm{GWR}}^{2}$ values rising to 0.97 for PD, FD, and TD, and reaching 0.94 for PD_mntd_ and 0.93 for FD_mntd_.

The high PD, FD, and TD values of Testudines are strongly linked to the main Amazon rivers (Figure 3p,r,t). Elevation was negatively associated with the PD, FD, and TD variation, with adjusted $R_{\mathrm{OLS}}^{2}$ values of 0.44, 0.43, and 0.36, respectively (all *p* < 0.001). GWR models provided robust fits for these associations, with adjusted $R_{\mathrm{GWR}}^{2}$ values reaching 0.92 for PD, 0.93 for FD, and 0.92 for TD (Figure 4p,r,t). In addition, high PD_mntd_ and FD_mntd_ values were observed in the northeastern and southeastern regions of the Amazon Basin (Figure 3q,s). GWR models for PD_mntd_ and FD_mntd_ also showed improved fits as well, with adjusted $R_{\mathrm{GWR}}^{2}$ values of 0.72 and 0.79, respectively (Figure 4q,s).

### 3.2 Spatial Correlations Among Diversity Metrics in Amphibians and Reptiles Lineages

**FIGURE 5:**
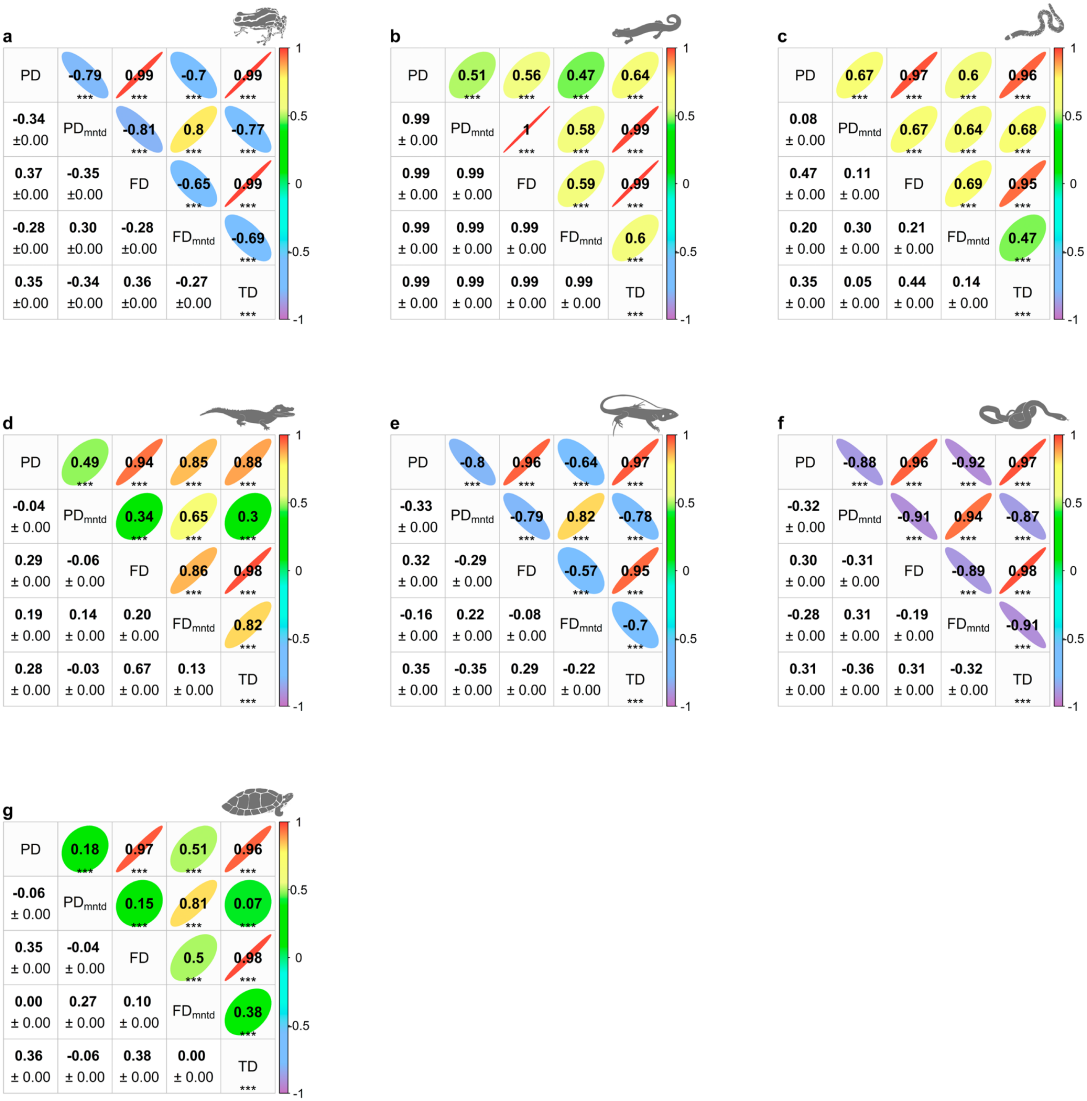
Correlation analysis among diversity metrics for amphibian (a: Anura, b: Caudata, c: Gymnophiona) and reptile (d: Crocodilians, e: Lizards, f: Snakes, g: Testudines) lineages. The upper diagonal shows the Pearson correlation coefficients (r), while the lower diagonal presents Tjøstheim's coefficient along with its variance. Each ellipse represents a pairwise comparison, where the direction indicates the sign (positive or negative) of the correlation, the color denotes the strength, and the thickness reflects the magnitude (with thinner ellipses indicating stronger correlations closer to ±1, and rounder ellipses indicating weaker correlations). Significance levels are indicated as follows: Ns *p* ≥ 0.05, * < 0.05; **p < 0.01; ***p < 0.001.

In the Anura lineage, significant spatial correlations were found among various diversity metrics. PD, FD, and TD are highly positively correlated, while PD_mntd_ and FD_mntd_ show negative correlations with PD, FD, and TD (Figure 5a). For Caudata, all spatial correlations showed a high positive relationship between the different diversity metrics (Figure 5b). In Gymnophionas, PD, FD, and TD are highly positively correlated. However, while positive correlations exceeding 60% were identified between PD_mntd_ with PD, FD, and TD, the spatial correlations for PD_mntd_ were notably weaker compared to the other metrics (Figure 5c).

Crocodilians exhibited a high positive correlation was observed between PD, FD, and TD (Figure 5d). Additionally, FD_mntd_ exhibited positive correlations of over 80% with PD, FD, and TD. However, spatial correlations for PD_mntd_ were weaker, with notably lower values compared to the overall correlations (Figure 5d).

For Lizard, PD, FD, and TD were highly positively correlated. In contrast, negative correlations over 64% were identified between PD_mntd_ and FD_mntd_ with PD, FD, and TD. The correlation between FD_mntd_ and FD was weaker than those observed with the other metrics (Figure 5e). In the case of Snakes, PD, FD, and TD displayed strong correlations, alongside a significant relationship between PD_mntd_ and FD_mntd_, exceeding 94% (Figure 5f). Notably, negative correlations of over 85% were identified between PD_mntd_ and FD_mntd_ with PD, FD, and TD (Figure 5f).

Lastly, Testudines showed high positive correlations among PD, FD, and TD. However, the spatial correlations for PD_mntd_ y FD_mntd_ with PD, FD, and TD were notably low (Figure 5g).

### 3.3 Testing Ecoregions

**FIGURE 6:**
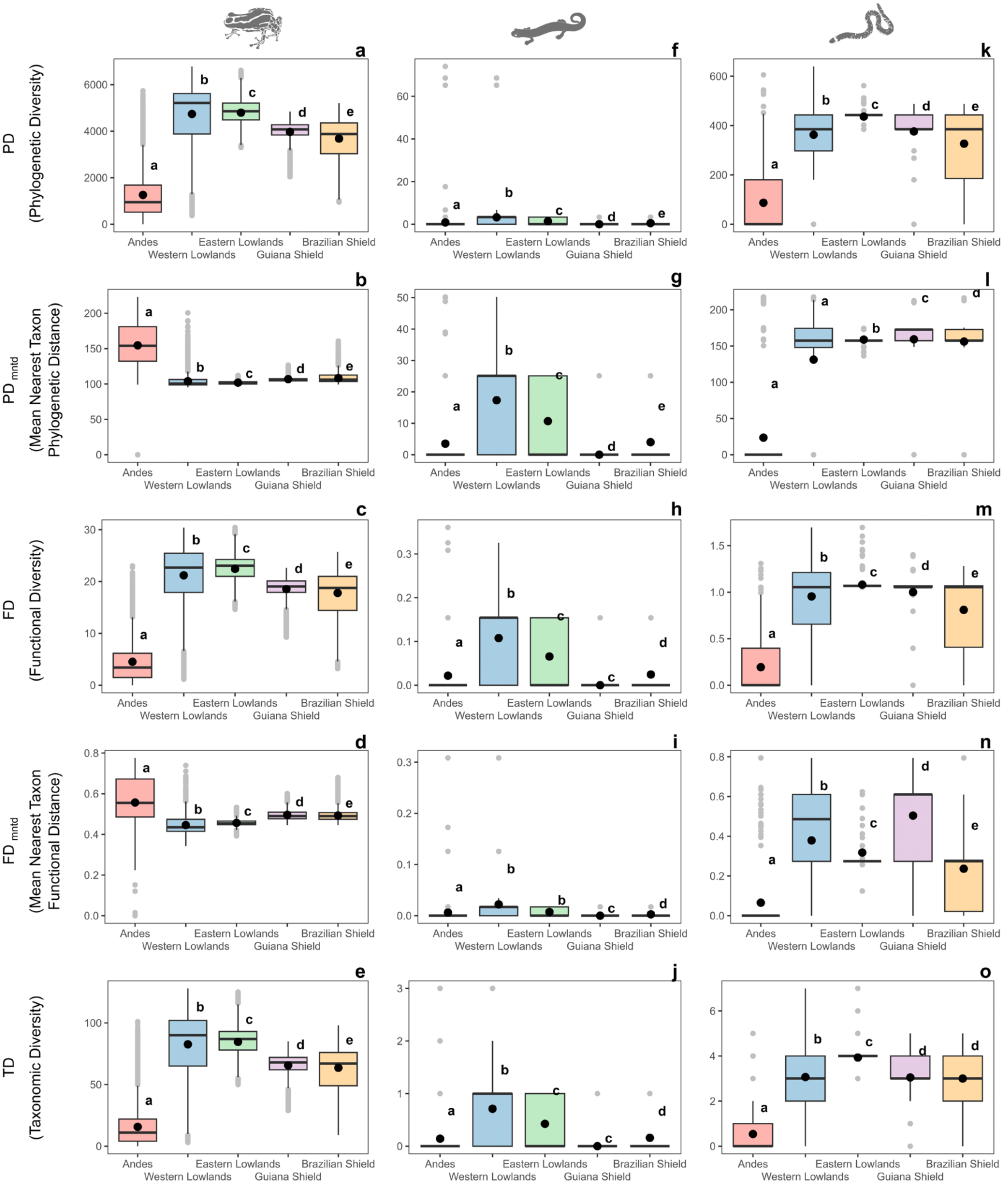
ANOVA with Tukey HSD test comparing ecoregions across multiple metrics of diversity distribution patterns for amphibian lineages in the Amazon Basin. Letters represent amphibian lineages (a–e: Anura; f–j: Caudata; k–o: Gymnophiona). Identical letters within each boxplot indicate ecoregions that are not significantly different, while different letters denote significant differences.

The analyses revealed significant differences across all metrics among the studied ecoregions for amphibian (*F* > 173, *p* < 0.001) and reptile (*F* > 3753, p < 0.001) lineages (Appendix S1, Tables S1.2 and S1.3). According to the post hoc Tukey analysis, anurans exhibited high values of PD, FD, and TD in the eastern and western lowlands, as well as the Guiana and Brazilian Shields (Figure 6a,c,e). In contrast, the values of PD_mntd_ and FD_mntd_ were high in the Andes (Figure 6b,d).

For Caudata, all metrics exhibited higher variation in the Eastern and Western Lowlands compared to the other ecoregions (Figure 6f–j).

In the case of Gymnophiona, the Eastern and Western Lowlands, along with the Guiana and Brazilian Shields, exhibited high values across all metrics, whereas the Andes showed low values across the five metrics (Figure 6k–o).

**FIGURE 7:**
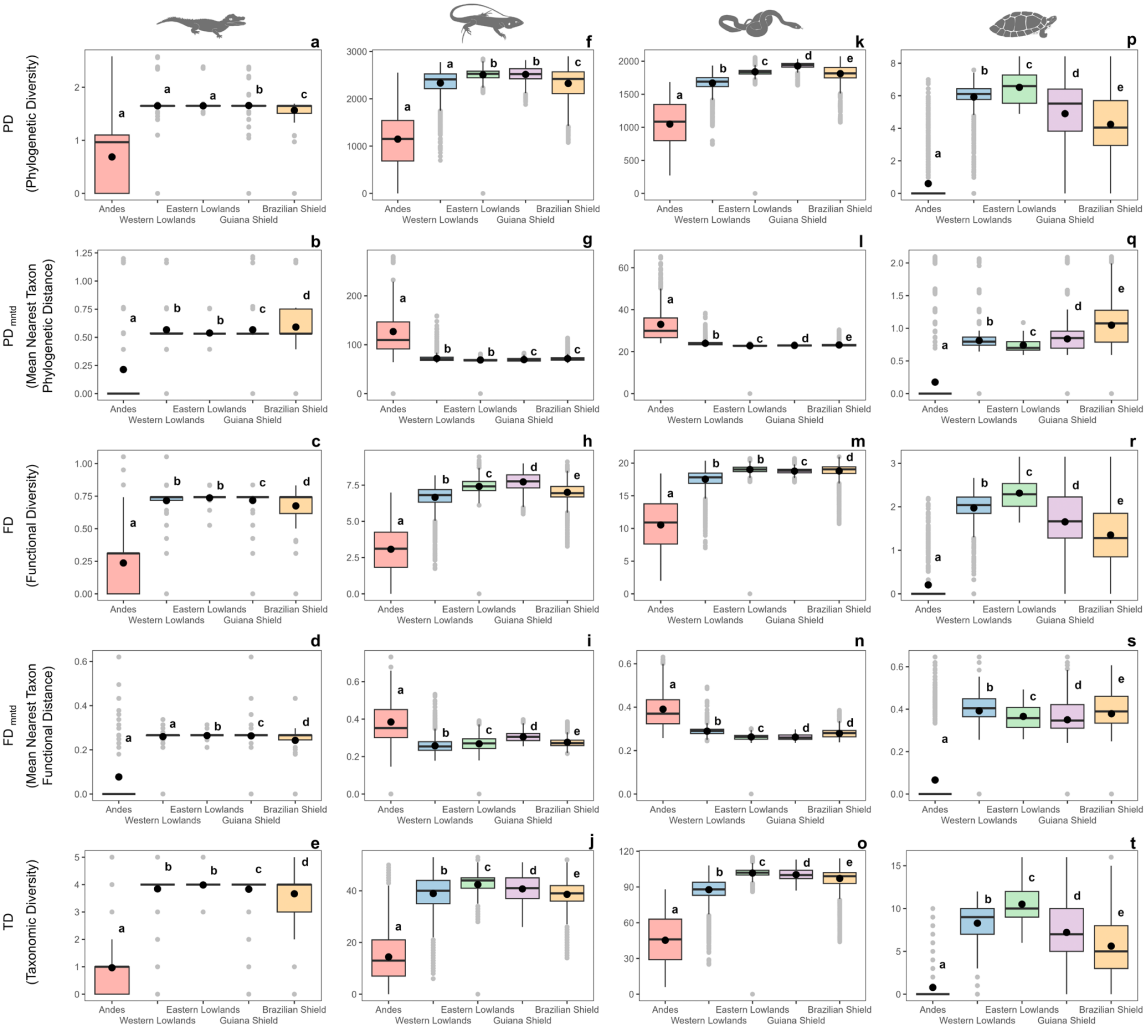
ANOVA with Tukey HSD test comparing ecoregions across multiple metrics of diversity distribution patterns for reptile lineages in the Amazon Basin. Letters represent reptile lineages (a–e: Crocodilians; f–j: Lizards; k–o: Snakes; p–t: Testudines). Identical letters within each boxplot indicate ecoregions that are not significantly different, while different letters denote significant differences.

Regarding crocodilians, the Guiana Shield, Eastern Lowlands, and Western Lowlands were phylogenetically similar and superior to the Andes and Brazilian Shield (Figure 7a). However, the Guiana Shield and Western Lowlands differed from the Eastern Lowlands in both FD and TD (Figure 7c,e). The Andes exhibited low values across all five metrics (Figure 7a–e).

Squamates exhibited high variations in PD, FD, and TD across the Eastern and Western Lowlands, as well as the Guiana and Brazilian Shields. In contrast, the Andes displayed higher PD_mntd_ and FD_mntd_ values compared to the other ecoregions (Figure 7f–o). For lizards, the Guiana Shield and Eastern Lowlands were similar in PD, distinguishing themselves from the other ecoregions (Figure 7f). In terms of FD and TD, all regions exhibited significant differences (Figure 7h,j). For snakes, the Guiana and Brazilian Shields were similar in FD, contrasting with the other ecoregions (Figure 7m). Regarding PD and TD, all regions showed significant differences (Figure 7k,o).

Finally, Testudines showed high PD, FD, and TD values in the Eastern and Western Lowlands, while cratonic regions displayed high PD_mntd_ values, and the Guiana Shield and Western Lowlands showed high FD_mntd_ values. In contrast, the Andes exhibited low values across all five metrics (Figure 7p–t).

## 4 Discussion

### 4.1 Diversity Patterns of Amphibians and Reptiles

We used three metrics of diversity to explore how the distribution patterns of lineages of Amazonian amphibians and reptiles vary spatially. Our research reveals that each lineage exhibited different spatial patterns in each of the diversity metrics. Our findings highlight the Western Lowlands as the largest hotspot for amphibians' phylogenetic, functional and taxonomic diversity. For instance, anuran PD, FD, and TD peaked at elevations ranging from 60 to 800 m.a.s.l., particularly in regions north of the eastern montane forests of the Napo sub‐basin (All the metrics for Caudata were also high in this region), the Iquitos region, and the Juruá sub‐basin. These geographical areas are closely linked to precipitation, temperature, and seasonal evapotranspiration patterns in the Amazon Basin (Maeda et al. 2017), which are robust predictors of amphibian diversity (Ochoa‐Ochoa et al. 2019; Ochoa‐Ochoa et al. 2020).

However, not all lineages in our study follow this pattern. For example, the distribution of squamates largely coincides with regions at altitudes below 1800 m.a.s.l. Snakes showed FD and TD hotspots in the Purus‐Madeira interfluve and the northeastern and northwestern regions of the Amazon Basin, with lizards showing a similar distribution of PD, FD, and TD. The three metrics of Testudine diversity are strongly related to the main Amazonian rivers, close to equatorial latitudes. Our findings reflect, for the first time, the distribution of both phylogenetic and functional diversity and divergence among Amazonian Testudines.

The distribution of Mean Nearest Taxon Distance (MNTD) values reveals distinct patterns of phylogenetic and functional divergence that reflect specific ecological adaptations and evolutionary processes for each group at different altitudes. In anurans, the high PD_mntd_ values at elevations above 1100 m.a.s.l. and FD_mntd_ values above 500 m.a.s.l. suggest that amphibian communities at higher elevations maintain species with large phylogenetic and functional distances from their closest relatives, indicating lower rates of recent speciation and preservation of ancient traits in these regions. For squamates, the high PD_mntd_ and FD_mntd_ values at elevations above 2000 m.a.s.l. suggest strong phylogenetic and functional divergence, possibly due to specific ecological pressures at high altitudes that favor differentiation.

In the lowlands and mid‐Amazon Basin, communities tend to be more phylogenetically and functionally similar, reflecting either a more recent species composition or ecological pressures that promote the coexistence of closely related species. Gymnophionans, on the other hand, exhibit greater phylogenetic and functional divergence across a wide altitudinal gradient, possibly reflecting adaptation to diverse subterranean microhabitats. Crocodilians display high PD_mntd_ and FD_mntd_ values at altitudes between 196 and 2915 m, mainly concentrated in the Central Andes, Brazilian Shield, and Rio Negro sub‐basin, indicating that these regions sustain differentiated lineages with specific ecological roles. In testudines, phylogenetic and functional divergences concentrate at intermediate altitudes, particularly in the Negro, Trombetas, Xingu, Tapajós, and Madeira sub‐basins, suggesting that these areas provide ecological conditions supporting both ancestral lineages and recent adaptations.

These divergence patterns suggest that ecological niches and evolutionary pressures differ significantly across altitudinal gradients, indicating potential habitat specialization or varying responses to climatic factors. Such variations may be partially explained by paleogeographic and paleoenvironmental processes in Amazonia throughout the Miocene (23–5 Mya) and potentially related to the Amazon Basin's altitudinal and latitudinal ranges, which impact biotic events in the lineages (Antonelli et al. 2010, 2018).

### 4.2 Metrics Correlation in Amphibians and Reptiles

The spatial relationship between diversity metrics was examined. Phylogenetic and functional diversity patterns were positively correlated with taxonomic diversity, although these relationships varied spatially among taxa. For example, in Caudata, taxonomic diversity only explained 64% of the phylogenetic diversity association. For the other amphibian and reptile lineages, taxonomic diversity explained more than 80% of the phylogenetic and functional diversity association. These associations indicate that the three diversity metrics were congruent in each taxon.

All amphibian and reptile lineages showed lower trait variability (high FD_mntd_ values) in the absence of close relatives (high PD_mntd_ values) indicating that functional differentiation is constrained when species are phylogenetically distant. This pattern is consistent with the idea that communities with few closely related species may have limited opportunities for niche differentiation. Conversely, regions with higher trait variability (low FD_mntd_ values) are associated with greater coexistence of closely related species, where competitive pressures may foster greater divergence in traits. This suggests that in regions with a higher concentration of phylogenetically similar species, competition can play a key role in driving functional diversification as species adapt to reduce niche overlap (Gerhold et al. 2015).

All lineages of amphibians and reptiles in the Amazon Basin exhibit a positive FD‐TD correlation, indicating that TD hotspots have ample ecological space and/or high ecological stability promoting trait dissimilarity and functional diversity, based on the natural history of each lineage. In contrast, in regions with low diversity, such as in high Andean altitudes, the observed patterns indicate that certain traits may be more conserved, likely due to environmental filtering favoring species adapted to hostile conditions (Navas 2006). Additionally, the patterns observed in sites with high PD values suggest low rates of recent speciation or high dispersal, as these areas exhibit a greater accumulation of phylogenetic diversity. Conversely, sites with lower PD and higher FD values may indicate ecological opportunities that promote functional diversification, possibly driven by mechanisms such as competitive interactions or adaptive radiation (Schluter 2000). Although the study does not directly measure speciation or dispersal events, the calculated diversity metrics align with the expected patterns in areas where ecological processes shape functional and phylogenetic diversity (Davies and Buckley 2011).

Anuran and squamate assemblages displayed higher diversity across all three metrics when coexisting with phylogenetically and functionally close relatives (low PD_mntd_ and FD_mntd_ values), indicating that phylogenetic and functional terminal divergence in the Andean region was more sensitive to changes in TD. The relationship between higher niche availability and the presence of species from various genera indicates a broader range of traits within the community, potentially leading to weaker competition due to greater trait variability, as suggested by Mouchet et al. (2010). While different lineages (with high PD_mntd_ values) with similar functional traits (with low FD values) may colonize regions or converge through habitat filtering (Ndiribe, Salamin, and Guisan 2013). However, as the geographic gradient transitions from the higher‐altitude Andean regions to the lowlands of the Amazon Basin, the absence of phylogenetically and functionally close relatives decreases as species richness increases. This pattern suggests a shift towards a more diverse community structure, where the coexistence of closely related species (species of the same genus) leads to greater trait divergence and functional diversity under conditions of overdispersion (Ndiribe, Salamin, and Guisan 2013).

By contrast, PD_mntd_ and FD_mntd_ for other amphibian (i.e., Caudata and Gymnophiona) and reptile (i.e., crocodilians and Testudines) lineages showed positive correlations with PD, FD, and TD. This suggests that phylogenetic and functional terminal clustering in the Amazon Basin's lower and middle regions was less sensitive to changes in taxonomic diversity. For instance, crocodilians and Testudines displayed medium to low associations when high evolutionary history sites are linked to interspecific interactions among species of the same genus with dissimilar traits. This pattern reflects the ancient lineage of these species and their relatively low evolutionary rates (Meseguer and Condamine 2020; Thomson, Spinks, and Shaffer 2021). In contrast, the strong associations in Gymnophiona suggest that their diversification has not been affected by their subterranean lifestyle for approximately 200 Myr (Jetz and Pyron 2018).

Our research reveals that species richness alone is insufficient for understanding ecological and evolutionary dynamics that affect species distributions in the Amazon Basin. Integrating taxonomic, phylogenetic, and functional diversity offers deeper insights into how processes like ecological opportunity, habitat filtering, and competition shape biodiversity patterns across landscapes (Ochoa‐Ochoa et al. 2020). Although the metrics analyzed did not differ significantly from a null model, suggesting an absence of strong environmental filtering or clustering, the complexity of community dynamics may involve subtle factors not captured in this study. Therefore, future research should explore these factors to better understand species distribution.

### 4.3 Amphibians and Reptiles Lineages of the Ecoregions

Our results reveal different ecological and evolutionary patterns of amphibian and reptile lineages between ecoregions. The findings reflect biogeographical patterns that resemble the cradle and museum hypothesis, indicating regions that maintain both ancient lineages and areas of active diversification.

The Andean region of the Amazon Basin contains less closely related terminal lineages in both anurans and squamates, indicating that geographic isolation and unique environmental pressures in these mountainous regions are associated with evolutionary divergence. In contrast, the western and eastern lowlands exhibit higher PD, FD, and TD values in anurans and squamates, which aligns with their role as hotspots for species richness where diverse lineages co‐occur. The co‐occurrence of closely related species in these regions contributes to lower PD_mntd_ and higher PD values.

However, in the lowlands and intermediate elevations of the cratonic regions (Brazilian and Guiana Shields), both anuran and squamate lineages reflect a mix of ancient lineages and recently diversified species, suggesting that these areas act as both museums, preserving lineages with deep evolutionary histories, and cradles for ongoing diversification. This dual role is evident in the higher PD and FD values observed across these regions for anurans and squamates, reflecting the presence of both ancient and newly emerging lineages. These variations may be attributed to MNTD, which assesses phylogenetic or functional relationships between co‐occurring species and quantifies distances between shallow nodes in the assemblage, whereas PD considers the time since species shared a common ancestor (Webb 2000).

The complex and varied biogeographic histories of each lineage contribute to the observed diversity patterns. In anurans, the presence of both ancient lineages like Archaeobatrachia and more recently diversified groups such as Neobatrachia, including diverse families like Hylidae, Bufonidae, and Craugastoridae (Feng et al. 2017; Hutter, Lambert, and Wiens 2017), likely contributes to the observed diversity patterns. For lizards, ancient lineages such as Sphaerodactylini (Gamble et al. 2011; Burbrink et al. 2020) and the diversification of more recent groups like *Stenocercus* (Torres‐Carvajal 2007) in the Andes also contribute to the variation in diversity metrics. The diversification of colubrid snakes during the Neogene period, spanning from approximately 23–2.6 million years ago (Serrano et al. 2024), corresponds to elevated PD values, especially within recently diversified genera such as *Atractus* (Serrano et al. 2024). These lineage‐specific histories likely shape the observed variation in diversity metrics across regions.

However, the other taxa followed different paths. The phylogenetic diversity of Gymnophionas, crocodilians, and testudines decreases with increasing altitude. At the same time as species richness increases (i.e., Gymnophionas, crocodilians, and testudines), the absence of species with distinct evolutionary origins (evidenced by high PD_mntd_ values) increases along their respective altitudinal ranges. These patterns may also be attributed to the evolutionary history and ecology of each lineage.

In the northwestern Amazon Basin humid forests, *Bolitoglossa* salamanders exhibit high values in all metrics. These patterns may reflect the known dispersal and diversification history of the genus in the region (Jaramillo et al. 2020). Except for the Andes, the other ecoregions present high phylogenetic diversity with the absence of close relatives (high PD_mntd_ values) in the Gymnophionas, reflecting conserved aspects of their function and physiology that may have remained stable despite past climatic events (Jetz and Pyron 2018; Torres‐Sánchez et al. 2019).

In the northwestern end Amazon Basin, crocodilians exhibit higher PD and FD values, likely due to the coexistence of species from both the Crocodylidae (e.g., *Crocodylus*) and Alligatoridae families (e.g., *Paleosuchus*, *Melanosuchus*, and *Caiman*). This pattern aligns with the diversification and radiation history of South American crocodilians (Salas‐Gismondi et al. 2015; Lourenço‐de‐Moraes et al. 2023). Conversely, the presence of less related terminal species at intermediate altitudes in the northern, western, and southeastern Amazon Basin suggests overdispersal may result from competition interactions among Alligatoridae species.

In the modern river system of the Amazon Basin and its lowlands, Testudines exhibit close relations to older lineages, particularly within the Pleurodira clade, which includes species dating back to the Upper Cretaceous and Eocene. This lineage likely experienced continuous diversification and low rates of recent speciation (Meseguer and Condamine 2020; Thomson, Spinks, and Shaffer 2021), highlighting their long‐standing presence in the Amazon. The high PD_mntd_ values are likely influenced by the presence of more recently emerged Cryptodira species (Meseguer and Condamine 2020; Thomson, Spinks, and Shaffer 2021), inhabiting neighboring biomes in the lower and middle regions of the Brazilian (around the Cerrado‐Amazonia transition) and the Guiana Shield, respectively. The cratonic rivers in these areas have a low flood pulse, unlike those near the equator and in the eastern Amazon Basin, which provide an ideal breeding habitat for most Amazonian testudines species.

The range of climates resulting from the elevation of the Andes strongly influences the physiological traits of organisms, acting as environmental filters that facilitate the aggregation of related species (Rangel et al. 2018). Andean uplift promoted geographic isolation and diversification (Hutter, Lambert, and Wiens 2017; Esquerré et al. 2019; Serrano et al. 2024), as reflected in the observed results, which indicate altitudinal patterns of less related terminal species (species of different genera). In contrast, the Amazon Basin's lowlands, with their relatively recent history compared to the Andes, serve as a significant biogeographic barrier, promoting in situ diversity and increasing phylogenetic diversity in these regions (Coronado et al. 2015; Rangel et al. 2018). This aligns with the diversity metrics reported in the study. Conversely, the ancient cratonic areas (Guiana and the Brazilian Shield), characterized by geological stability and rare vicariant events, have limited diversification in lineages (Antonelli et al. 2010; Val et al. 2021), which corresponds with the low diversity values observed in these regions.

From a conservation perspective, understanding whether a region functions as a cradle or a museum is key. Cradle ecoregions should prioritize habitat preservation to sustain processes driving diversity. Meanwhile, museum ecoregions require strategies focused on safeguarding unique lineages and habitats. Additionally, the high functional diversity in regions such as the Eastern and Western Lowlands highlights the need to maintain not just species but also their ecological roles to ensure ecosystem resilience. Overall, these diversity patterns underscore the need for conservation strategies tailored to the unique evolutionary and ecological dynamics of each region.

### 4.4 Limitations and Implications

In recent years, the description of new species of anurans and reptiles, together with the increase in collections and species lists, has helped to fill existing information gaps. However, this makes compiling, segregating, and obtaining accurate data on the species' natural history difficult. Due to the Linnean and Wallacean deficit (Hortal et al. 2015), we are far from having a fully resolved Tree of Life for these taxa. Nevertheless, we believe our database is sufficiently robust to construct a comprehensive macro list of amphibians and reptiles from the Amazon Basin, compiled from various reliable data sources and field observations. Furthermore, molecular phylogenetic analyses and functional traits of amphibian and reptile lineages provide us with a broad view of understanding the evolutionary and functional relationships between lineages.

Mountainous regions may offer limited insights into the dynamics of diversification in different groups (Vasconcelos, O'Meara, and Beaulieu 2022). However, our results suggest that this cradle and museum hypothesis should be carefully analyzed, as each taxon examined in this study exhibits distinct patterns. For instance, the outliers observed in the Andes across all metrics and lineages (see Figures 4 and 5) indicate that specific Andean regions could serve as both museums and centers of endemism. This highlights the unique evolutionary histories of species in the Amazon Basin, from colorful poison frogs to tree snakes and prehistoric caimans, contributing to its remarkable diversity of forms and functions. Additionally, the basin serves as an evolutionary “museum,” harboring lineages dating back to ancient times.

We revealed new patterns of phylogenetic, functional, and taxonomic diversity of amphibian and reptile lineages in the Amazon Basin. The three diversity metrics exhibited relatively congruent patterns within each amphibian and reptile lineage, attributed to ecological and evolutionary processes. Our study indicates that the lower and middle regions of the Amazon Basin were characterized by high phylogenetic, functional, and taxonomic diversity. Phylogenetic and functional divergence metrics varied among lineages. In the Andean regions, lineages with higher species richness had fewer related terminal species. In addition, our results suggest that conservation efforts should prioritize both “cradle” regions, where active diversification occurs, and “museum” regions that preserve ancient lineages. By focusing on areas with high functional diversity, conservation strategies can also ensure the protection of key ecosystem services, such as nutrient cycling and seed dispersal, which are critical for the resilience of Amazonian ecosystems.

## Author Contributions

**Jhon Jairo López‐Rojas:** conceptualization (equal), data curation (equal), formal analysis (lead), methodology (equal), visualization (lead), writing – original draft (lead), writing – review and editing (equal). **Diego Henrique Santiago:** data curation (equal), writing – review and editing (supporting). **Mirco Solé:** writing – review and editing (supporting). **Ricardo Lourenço‐de‐Moraes:** conceptualization (equal), formal analysis (supporting), methodology (equal), writing – original draft (equal), writing – review and editing (equal).

## Ethics Statement

The authors have nothing to report.

## Consent

The authors have nothing to report.

## Conflicts of Interest

The authors declare no conflicts of interest.

## Supporting information


Appendix S1.

Appendix S2.

Appendix S3.

Appendix S4.

## Data Availability

Data are available publicly from the following sources:
We used the IUCN Redlist advanced search (https://www.iucnredlist.org/search/grid), with two filters: “Taxonomy” and “Land Region”. For “Taxonomy”, we selected the Amphibian lineages and crocodilians. In “Land Region”, we selected nine countries surrounding the Amazon basin. In “Download, the Range data” the “Polygons (.shp)” option was used. Ranges maps of Testudines species were taken from “Turtles of the World: Annotated Checklist and Atlas of Taxonomy, Synonymy, Distribution, and Conservation Status (9th Ed.)”, by Rhodin et al. (2021). In the acknowledgments, section we thank the author, Anders Rhodin, for sending us digital copies. The distribution maps for Squamata (lizards and snakes), in shapefile format, were obtained from the current version of the Global Assessment of Reptile Distributions (GARD; http://www.gardinitiative.org/). The GARD 1.7 database is accessible at the following link: https://doi.org/10.5061/dryad.9cnp5hqmb
Three time‐calibrated phylogenies were retrieved. For amphibians, they were taken from “The Interplay of Past Diversification and Evolutionary Isolation with Present Imperilment across the Amphibian Tree of Life” by Jetz and Pyron (2018), https://doi.org/10.1038/s41559‐018‐0515‐5. Squamate reptiles were taken from “Fully‐Sampled Phylogenies of Squamates Reveal Evolutionary Patterns in Threat Status” by Tonini et al. (2016), https://doi.org/10.1016/j.biocon.2016.03.039. For Crocodilians and Testudines, we used “Phylogenetic and Spatial Distribution of Evolutionary Diversification, Isolation, and Threat in Turtles and Crocodilians (Non‐Avian Archosauromorphs).” by Colston et al. (2020), https://doi.org/10.1186/s12862‐020‐01642‐3
