# Supplementary material for: Amphibians and Reptiles Exhibit Different Ecological and Evolutionary Spatial Patterns in the Amazon Basin
Source: Ecol Evol. 2025 Mar 19;15(3):e70677. doi: 10.1002/ece3.70677 (PMC11922541; doi:10.1002/ece3.70677)
Supplement: Supplementary file 1 [file ECE3-15-e70677-s003.docx]

Supporting information

Amphibians and reptiles exhibit different ecological and evolutionary spatial patterns in the Amazon Basin

López-Rojas, Jhon Jairo1,2; Santiago, Diego Henrique3; Solé, Mirco1,4; Lourenço-de-Moraes, Ricardo5. 

Supplemental table

[Table S1.1: Species list of amphibians and reptiles of the Amazon Basin.	2](#__RefHeading__27263_422032560)

[Table S1.2: ANOVA and Tukey HSD test comparing diversity metrics of amphibian lineages across ecoregions in the Amazon Basin.	45](#__RefHeading__27265_422032560)

[Table S1.3: ANOVA and Tukey HSD test comparing diversity metrics of reptile lineages across ecoregions in the Amazon Basin.	51](#__RefHeading__27267_422032560)

[Figure S1.1: Additional sampling points located in the Amazon Basin. Sites: 1) Reserva Nacional Pacaya Samiria; 2) Centro de Investigación y Enseñanza Forestal (CIEFOR); 3) Reserva Forestal Santa Cruz; 4) Reserva Nacional Allpahuayo Mishana; 5) Iquitos; 6) Project Amazon; 7) Requena; 8) Catuaba; 9) Balbina; 10) Presidente Figueredo; 11) Segunda Jerusalén; 12) Aguas Calientes – Huacharos; 13) Inkaterra; 14) Juninjillo; 15) Mâncio Lima; 16) Mashira; 17) Puerto Elvira; 18) Soledad; 19) Salvador Pava; 20) Pintoyacu; 21) Alto Nanay; 22) Cuenca Rio Itaya; 23) Tamshiyacu; 24) Situche Norte 25) Bretaña; 26) Machinguenga; 27) Chambira.	56](#__RefHeading__27269_422032560)

[Figure S1.2: Comparison of Olson et al. (2001) Ecoregion Classification (a) with the Present Study's Reclassified Ecoregions (b) in the Amazon Basin. 	58](#__RefHeading__27271_422032560)

# Table S1.1: Species list of amphibians and reptiles of the Amazon Basin.

| Taxa | Taxomomic group | Specie | Reference |
| --- | --- | --- | --- |
| Amphibia | Anura | Adelastes hylonomos | IUCN, 2024 |
| Amphibia | Anura | Adelophryne adiastola | IUCN, 2024; Present study |
| Amphibia | Anura | Adelophryne gutturosa | IUCN, 2024 |
| Amphibia | Anura | Adelphobates castaneoticus | IUCN, 2024 |
| Amphibia | Anura | Adelphobates galactonotus | IUCN, 2024 |
| Amphibia | Anura | Adelphobates quinquevittatus | IUCN, 2024 |
| Amphibia | Anura | Adenomera andreae | IUCN, 2024; Present study |
| Amphibia | Anura | Adenomera coca | IUCN, 2024 |
| Amphibia | Anura | Adenomera diptyx | IUCN, 2024 |
| Amphibia | Anura | Adenomera heyeri | IUCN, 2024 |
| Amphibia | Anura | Adenomera hylaedactyla | IUCN, 2024; Present study |
| Amphibia | Anura | Adenomera lutzi | IUCN, 2024 |
| Amphibia | Anura | Adenomera martinezi | IUCN, 2024 |
| Amphibia | Anura | Agalychnis buckleyi | IUCN, 2024 |
| Amphibia | Anura | Agalychnis hulli | IUCN, 2024 |
| Amphibia | Anura | Allobates alessandroi | IUCN, 2024 |
| Amphibia | Anura | Allobates brunneus | IUCN, 2024 |
| Amphibia | Anura | Allobates caeruleodactylus | IUCN, 2024 |
| Amphibia | Anura | Allobates conspicuus | IUCN, 2024; Present study |
| Amphibia | Anura | Allobates crombiei | IUCN, 2024 |
| Amphibia | Anura | Allobates femoralis | IUCN, 2024; Present study |
| Amphibia | Anura | Allobates fratisenescus | IUCN, 2024 |
| Amphibia | Anura | Allobates fuscellus | IUCN, 2024 |
| Amphibia | Anura | Allobates gasconi | IUCN, 2024 |
| Amphibia | Anura | Allobates granti | IUCN, 2024 |
| Amphibia | Anura | Allobates insperatus | IUCN, 2024; Present study |
| Amphibia | Anura | Allobates kingsburyi | IUCN, 2024 |
| Amphibia | Anura | Allobates marchesianus | IUCN, 2024; Present study |
| Amphibia | Anura | Allobates masniger | IUCN, 2024 |
| Amphibia | Anura | Allobates mcdiarmidi | IUCN, 2024 |
| Amphibia | Anura | Allobates melanolaemus | IUCN, 2024 |
| Amphibia | Anura | Allobates myersi | IUCN, 2024 |
| Amphibia | Anura | Allobates nidicola | IUCN, 2024 |
| Amphibia | Anura | Allobates ornatus | IUCN, 2024 |
| Amphibia | Anura | Allobates paleovarzensis | IUCN, 2024 |
| Amphibia | Anura | Allobates picachos | IUCN, 2024 |
| Amphibia | Anura | Allobates subfolionidificans | IUCN, 2024; Present study |
| Amphibia | Anura | Allobates sumtuosus | IUCN, 2024; Present study |
| Amphibia | Anura | Allobates trilineatus | IUCN, 2024; Present study |
| Amphibia | Anura | Allobates vanzolinius | IUCN, 2024 |
| Amphibia | Anura | Allobates zaparo | IUCN, 2024; Present study |
| Amphibia | Anura | Allophryne ruthveni | IUCN, 2024 |
| Amphibia | Anura | Amazophrynella bokermanni | IUCN, 2024 |
| Amphibia | Anura | Amazophrynella minuta | IUCN, 2024 |
| Amphibia | Anura | Ameerega bassleri | IUCN, 2024 |
| Amphibia | Anura | Ameerega bilinguis | IUCN, 2024 |
| Amphibia | Anura | Ameerega boliviana | IUCN, 2024 |
| Amphibia | Anura | Ameerega braccata | IUCN, 2024 |
| Amphibia | Anura | Ameerega cainarachi | IUCN, 2024 |
| Amphibia | Anura | Ameerega flavopicta | IUCN, 2024 |
| Amphibia | Anura | Ameerega hahneli | IUCN, 2024; Present study |
| Amphibia | Anura | Ameerega ignipedis | IUCN, 2024 |
| Amphibia | Anura | Ameerega ingeri | IUCN, 2024 |
| Amphibia | Anura | Ameerega macero | IUCN, 2024; Present study |
| Amphibia | Anura | Ameerega parvula | IUCN, 2024 |
| Amphibia | Anura | Ameerega pepperi | IUCN, 2024 |
| Amphibia | Anura | Ameerega petersi | IUCN, 2024 |
| Amphibia | Anura | Ameerega picta | IUCN, 2024; Present study |
| Amphibia | Anura | Ameerega planipaleae | IUCN, 2024 |
| Amphibia | Anura | Ameerega pongoensis | IUCN, 2024 |
| Amphibia | Anura | Ameerega rubriventris | IUCN, 2024 |
| Amphibia | Anura | Ameerega shihuemoy | IUCN, 2024 |
| Amphibia | Anura | Ameerega silverstonei | IUCN, 2024 |
| Amphibia | Anura | Ameerega simulans | IUCN, 2024 |
| Amphibia | Anura | Ameerega smaragdina | IUCN, 2024 |
| Amphibia | Anura | Ameerega trivittata | IUCN, 2024; Present study |
| Amphibia | Anura | Ameerega yoshina | IUCN, 2024 |
| Amphibia | Anura | Ameerega yungicola | IUCN, 2024 |
| Amphibia | Anura | Andinobates abditus | IUCN, 2024 |
| Amphibia | Anura | Anomaloglossus baeobatrachus | IUCN, 2024 |
| Amphibia | Anura | Anomaloglossus beebei | IUCN, 2024 |
| Amphibia | Anura | Anomaloglossus kaiei | IUCN, 2024 |
| Amphibia | Anura | Anomaloglossus leopardus | IUCN, 2024 |
| Amphibia | Anura | Anomaloglossus parimae | IUCN, 2024 |
| Amphibia | Anura | Anomaloglossus stepheni | IUCN, 2024 |
| Amphibia | Anura | Anomaloglossus tamacuarensis | IUCN, 2024 |
| Amphibia | Anura | Aparasphenodon venezolanus | IUCN, 2024 |
| Amphibia | Anura | Atelopus andinus | IUCN, 2024 |
| Amphibia | Anura | Atelopus angelito | IUCN, 2024 |
| Amphibia | Anura | Atelopus ardila | IUCN, 2024 |
| Amphibia | Anura | Atelopus bomolochos | IUCN, 2024 |
| Amphibia | Anura | Atelopus boulengeri | IUCN, 2024 |
| Amphibia | Anura | Atelopus dimorphus | IUCN, 2024 |
| Amphibia | Anura | Atelopus epikeisthos | IUCN, 2024 |
| Amphibia | Anura | Atelopus erythropus | IUCN, 2024 |
| Amphibia | Anura | Atelopus exiguus | IUCN, 2024 |
| Amphibia | Anura | Atelopus gigas | IUCN, 2024 |
| Amphibia | Anura | Atelopus halihelos | IUCN, 2024 |
| Amphibia | Anura | Atelopus ignescens | IUCN, 2024 |
| Amphibia | Anura | Atelopus loettersi | IUCN, 2024 |
| Amphibia | Anura | Atelopus nanay | IUCN, 2024 |
| Amphibia | Anura | Atelopus nepiozomus | IUCN, 2024 |
| Amphibia | Anura | Atelopus onorei | IUCN, 2024 |
| Amphibia | Anura | Atelopus orcesi | IUCN, 2024 |
| Amphibia | Anura | Atelopus oxapampae | IUCN, 2024 |
| Amphibia | Anura | Atelopus pachydermus | IUCN, 2024 |
| Amphibia | Anura | Atelopus palmatus | IUCN, 2024 |
| Amphibia | Anura | Atelopus pastuso | IUCN, 2024 |
| Amphibia | Anura | Atelopus patazensis | IUCN, 2024 |
| Amphibia | Anura | Atelopus peruensis | IUCN, 2024 |
| Amphibia | Anura | Atelopus petersi | IUCN, 2024 |
| Amphibia | Anura | Atelopus petriruizi | IUCN, 2024 |
| Amphibia | Anura | Atelopus planispina | IUCN, 2024 |
| Amphibia | Anura | Atelopus podocarpus | IUCN, 2024 |
| Amphibia | Anura | Atelopus pulcher | IUCN, 2024; Present study |
| Amphibia | Anura | Atelopus pyrodactylus | IUCN, 2024 |
| Amphibia | Anura | Atelopus reticulatus | IUCN, 2024 |
| Amphibia | Anura | Atelopus seminiferus | IUCN, 2024 |
| Amphibia | Anura | Atelopus simulatus | IUCN, 2024 |
| Amphibia | Anura | Atelopus siranus | IUCN, 2024 |
| Amphibia | Anura | Atelopus spumarius | IUCN, 2024; Present study |
| Amphibia | Anura | Atelopus tricolor | IUCN, 2024 |
| Amphibia | Anura | Boana aguilari | IUCN, 2024 |
| Amphibia | Anura | Boana albopunctata | IUCN, 2024 |
| Amphibia | Anura | Boana balzani | IUCN, 2024 |
| Amphibia | Anura | Boana boans | IUCN, 2024; Present study |
| Amphibia | Anura | Boana calcarata | IUCN, 2024; Present study |
| Amphibia | Anura | Boana callipleura | IUCN, 2024 |
| Amphibia | Anura | Boana cinerascens | IUCN, 2024; Present study |
| Amphibia | Anura | Boana crepitans | IUCN, 2024 |
| Amphibia | Anura | Boana dentei | IUCN, 2024 |
| Amphibia | Anura | Boana fasciata | IUCN, 2024; Present study |
| Amphibia | Anura | Boana geographica | IUCN, 2024; Present study |
| Amphibia | Anura | Boana gladiator | IUCN, 2024 |
| Amphibia | Anura | Boana hobbsi | IUCN, 2024 |
| Amphibia | Anura | Boana hutchinsi | IUCN, 2024 |
| Amphibia | Anura | Boana lanciformis | IUCN, 2024; Present study |
| Amphibia | Anura | Boana lemai | IUCN, 2024 |
| Amphibia | Anura | Boana leucocheila | IUCN, 2024 |
| Amphibia | Anura | Boana marianitae | IUCN, 2024 |
| Amphibia | Anura | Boana melanopleura | IUCN, 2024 |
| Amphibia | Anura | Boana microderma | IUCN, 2024 |
| Amphibia | Anura | Boana multifasciata | IUCN, 2024; Present study |
| Amphibia | Anura | Boana nympha | IUCN, 2024; Present study |
| Amphibia | Anura | Boana ornatissima | IUCN, 2024 |
| Amphibia | Anura | Boana palaestes | IUCN, 2024 |
| Amphibia | Anura | Boana pellucens | IUCN, 2024 |
| Amphibia | Anura | Boana punctata | IUCN, 2024; Present study |
| Amphibia | Anura | Boana raniceps | IUCN, 2024 |
| Amphibia | Anura | Boana riojana | IUCN, 2024 |
| Amphibia | Anura | Boana roraima | IUCN, 2024 |
| Amphibia | Anura | Boana sibleszi | IUCN, 2024 |
| Amphibia | Anura | Boana tepuiana | IUCN, 2024 |
| Amphibia | Anura | Boana tetete | IUCN, 2024 |
| Amphibia | Anura | Boana wavrini | IUCN, 2024 |
| Amphibia | Anura | Bryophryne abramalagae | IUCN, 2024 |
| Amphibia | Anura | Bryophryne bakersfield | IUCN, 2024 |
| Amphibia | Anura | Bryophryne bustamantei | IUCN, 2024 |
| Amphibia | Anura | Bryophryne cophites | IUCN, 2024 |
| Amphibia | Anura | Bryophryne flammiventris | IUCN, 2024 |
| Amphibia | Anura | Bryophryne gymnotis | IUCN, 2024 |
| Amphibia | Anura | Bryophryne hanssaueri | IUCN, 2024 |
| Amphibia | Anura | Bryophryne nubilosus | IUCN, 2024 |
| Amphibia | Anura | Bryophryne phuyuhampatu | IUCN, 2024 |
| Amphibia | Anura | Bryophryne zonalis | IUCN, 2024 |
| Amphibia | Anura | Callimedusa atelopoides | IUCN, 2024 |
| Amphibia | Anura | Callimedusa baltea | IUCN, 2024 |
| Amphibia | Anura | Callimedusa duellmani | IUCN, 2024 |
| Amphibia | Anura | Callimedusa ecuatoriana | IUCN, 2024 |
| Amphibia | Anura | Callimedusa perinesos | IUCN, 2024 |
| Amphibia | Anura | Callimedusa tomopterna | IUCN, 2024; Present study |
| Amphibia | Anura | Centrolene azulae | IUCN, 2024 |
| Amphibia | Anura | Centrolene bacatum | IUCN, 2024 |
| Amphibia | Anura | Centrolene buckleyi | IUCN, 2024 |
| Amphibia | Anura | Centrolene condor | IUCN, 2024 |
| Amphibia | Anura | Centrolene geckoideum | IUCN, 2024 |
| Amphibia | Anura | Centrolene hesperium | IUCN, 2024 |
| Amphibia | Anura | Centrolene huilense | IUCN, 2024 |
| Amphibia | Anura | Centrolene hybrida | IUCN, 2024 |
| Amphibia | Anura | Centrolene lemniscatum | IUCN, 2024 |
| Amphibia | Anura | Centrolene medemi | IUCN, 2024 |
| Amphibia | Anura | Centrolene muelleri | IUCN, 2024 |
| Amphibia | Anura | Centrolene pipilata | IUCN, 2024 |
| Amphibia | Anura | Centrolene sabini | IUCN, 2024 |
| Amphibia | Anura | Centrolene sanchezi | IUCN, 2024 |
| Amphibia | Anura | Centrolene solitaria | IUCN, 2024 |
| Amphibia | Anura | Ceratophrys cornuta | IUCN, 2024; Present study |
| Amphibia | Anura | Ceratophrys cranwelli | IUCN, 2024 |
| Amphibia | Anura | Ceratophrys testudo | IUCN, 2024 |
| Amphibia | Anura | Ceuthomantis aracamuni | IUCN, 2024 |
| Amphibia | Anura | Ceuthomantis cavernibardus | IUCN, 2024 |
| Amphibia | Anura | Chiasmocleis albopunctata | IUCN, 2024 |
| Amphibia | Anura | Chiasmocleis anatipes | IUCN, 2024 |
| Amphibia | Anura | Chiasmocleis antenori | IUCN, 2024; Present study |
| Amphibia | Anura | Chiasmocleis avilapiresae | IUCN, 2024 |
| Amphibia | Anura | Chiasmocleis bassleri | IUCN, 2024; Present study |
| Amphibia | Anura | Chiasmocleis carvalhoi | IUCN, 2024; Present study |
| Amphibia | Anura | Chiasmocleis devriesi | IUCN, 2024 |
| Amphibia | Anura | Chiasmocleis hudsoni | IUCN, 2024 |
| Amphibia | Anura | Chiasmocleis jimi | IUCN, 2024 |
| Amphibia | Anura | Chiasmocleis magnova | IUCN, 2024; Present study |
| Amphibia | Anura | Chiasmocleis mehelyi | IUCN, 2024 |
| Amphibia | Anura | Chiasmocleis shudikarensis | IUCN, 2024 |
| Amphibia | Anura | Chiasmocleis tridactyla | IUCN, 2024; Present study |
| Amphibia | Anura | Chiasmocleis ventrimaculata | IUCN, 2024; Present study |
| Amphibia | Anura | Chimerella corleone | IUCN, 2024 |
| Amphibia | Anura | Chimerella mariaelenae | IUCN, 2024 |
| Amphibia | Anura | Cochranella erminea | IUCN, 2024 |
| Amphibia | Anura | Cochranella guayasamini | IUCN, 2024 |
| Amphibia | Anura | Cochranella nola | IUCN, 2024 |
| Amphibia | Anura | Cochranella phryxa | IUCN, 2024 |
| Amphibia | Anura | Cochranella resplendens | IUCN, 2024 |
| Amphibia | Anura | Cochranella riveroi | IUCN, 2024 |
| Amphibia | Anura | Colostethus jacobuspetersi | IUCN, 2024 |
| Amphibia | Anura | Colostethus mertensi | IUCN, 2024 |
| Amphibia | Anura | Colostethus poecilonotus | IUCN, 2024 |
| Amphibia | Anura | Cruziohyla craspedopus | IUCN, 2024; Present study |
| Amphibia | Anura | Ctenophryne aequatorialis | IUCN, 2024 |
| Amphibia | Anura | Ctenophryne barbatula | IUCN, 2024 |
| Amphibia | Anura | Ctenophryne carpish | IUCN, 2024 |
| Amphibia | Anura | Ctenophryne geayi | IUCN, 2024; Present study |
| Amphibia | Anura | Dendrobates leucomelas | IUCN, 2024 |
| Amphibia | Anura | Dendrobates tinctorius | IUCN, 2024 |
| Amphibia | Anura | Dendropsophus acreanus | IUCN, 2024 |
| Amphibia | Anura | Dendropsophus anataliasiasi | IUCN, 2024 |
| Amphibia | Anura | Dendropsophus aperomeus | IUCN, 2024 |
| Amphibia | Anura | Dendropsophus bifurcus | IUCN, 2024 |
| Amphibia | Anura | Dendropsophus bokermanni | IUCN, 2024 |
| Amphibia | Anura | Dendropsophus brevifrons | IUCN, 2024; Present study |
| Amphibia | Anura | Dendropsophus cachimbo | IUCN, 2024 |
| Amphibia | Anura | Dendropsophus coffea | IUCN, 2024 |
| Amphibia | Anura | Dendropsophus delarivai | IUCN, 2024 |
| Amphibia | Anura | Dendropsophus frosti | IUCN, 2024 |
| Amphibia | Anura | Dendropsophus haraldschultzi | IUCN, 2024; Present study |
| Amphibia | Anura | Dendropsophus joannae | IUCN, 2024 |
| Amphibia | Anura | Dendropsophus juliani | IUCN, 2024 |
| Amphibia | Anura | Dendropsophus koechlini | IUCN, 2024; Present study |
| Amphibia | Anura | Dendropsophus leali | IUCN, 2024; Present study |
| Amphibia | Anura | Dendropsophus leucophyllatus | IUCN, 2024; Present study |
| Amphibia | Anura | Dendropsophus manonegra | IUCN, 2024 |
| Amphibia | Anura | Dendropsophus marmoratus | IUCN, 2024; Present study |
| Amphibia | Anura | Dendropsophus mathiassoni | IUCN, 2024 |
| Amphibia | Anura | Dendropsophus melanargyreus | IUCN, 2024 |
| Amphibia | Anura | Dendropsophus microcephalus | IUCN, 2024 |
| Amphibia | Anura | Dendropsophus minimus | IUCN, 2024 |
| Amphibia | Anura | Dendropsophus minutus | IUCN, 2024; Present study |
| Amphibia | Anura | Dendropsophus miyatai | IUCN, 2024 |
| Amphibia | Anura | Dendropsophus nanus | IUCN, 2024 |
| Amphibia | Anura | Dendropsophus parviceps | IUCN, 2024; Present study |
| Amphibia | Anura | Dendropsophus pauiniensis | IUCN, 2024 |
| Amphibia | Anura | Dendropsophus reichlei | IUCN, 2024 |
| Amphibia | Anura | Dendropsophus rhodopeplus | IUCN, 2024; Present study |
| Amphibia | Anura | Dendropsophus riveroi | IUCN, 2024 |
| Amphibia | Anura | Dendropsophus rossalleni | IUCN, 2024 |
| Amphibia | Anura | Dendropsophus rubicundulus | IUCN, 2024 |
| Amphibia | Anura | Dendropsophus sarayacuensis | IUCN, 2024; Present study |
| Amphibia | Anura | Dendropsophus schubarti | IUCN, 2024 |
| Amphibia | Anura | Dendropsophus timbeba | IUCN, 2024 |
| Amphibia | Anura | Dendropsophus tintinnabulum | IUCN, 2024 |
| Amphibia | Anura | Dendropsophus triangulum | IUCN, 2024; Present study |
| Amphibia | Anura | Dendropsophus tritaeniatus | IUCN, 2024 |
| Amphibia | Anura | Dendropsophus vraemi | IUCN, 2024 |
| Amphibia | Anura | Dendropsophus walfordi | IUCN, 2024 |
| Amphibia | Anura | Dendropsophus xapuriensis | IUCN, 2024 |
| Amphibia | Anura | Dermatonotus muelleri | IUCN, 2024 |
| Amphibia | Anura | Dryaderces inframaculata | IUCN, 2024 |
| Amphibia | Anura | Dryaderces pearsoni | IUCN, 2024 |
| Amphibia | Anura | Edalorhina nasuta | IUCN, 2024 |
| Amphibia | Anura | Edalorhina perezi | IUCN, 2024; Present study |
| Amphibia | Anura | Elachistocleis bicolor | IUCN, 2024; Present study |
| Amphibia | Anura | Elachistocleis helianneae | IUCN, 2024 |
| Amphibia | Anura | Elachistocleis matogrosso | IUCN, 2024 |
| Amphibia | Anura | Elachistocleis ovalis | IUCN, 2024 |
| Amphibia | Anura | Elachistocleis surumu | IUCN, 2024 |
| Amphibia | Anura | Engystomops freibergi | IUCN, 2024 |
| Amphibia | Anura | Engystomops petersi | IUCN, 2024; Present study |
| Amphibia | Anura | Epipedobates anthonyi | IUCN, 2024 |
| Amphibia | Anura | Espadarana audax | IUCN, 2024 |
| Amphibia | Anura | Espadarana durrellorum | IUCN, 2024 |
| Amphibia | Anura | Espadarana prosoblepon | IUCN, 2024 |
| Amphibia | Anura | Excidobates captivus | IUCN, 2024 |
| Amphibia | Anura | Excidobates condor | IUCN, 2024 |
| Amphibia | Anura | Excidobates mysteriosus | IUCN, 2024 |
| Amphibia | Anura | Gastrotheca abdita | IUCN, 2024 |
| Amphibia | Anura | Gastrotheca aguaruna | IUCN, 2024 |
| Amphibia | Anura | Gastrotheca andaquiensis | IUCN, 2024 |
| Amphibia | Anura | Gastrotheca antoniiochoai | IUCN, 2024 |
| Amphibia | Anura | Gastrotheca aratia | IUCN, 2024 |
| Amphibia | Anura | Gastrotheca argenteovirens | IUCN, 2024 |
| Amphibia | Anura | Gastrotheca atympana | IUCN, 2024 |
| Amphibia | Anura | Gastrotheca carinaceps | IUCN, 2024 |
| Amphibia | Anura | Gastrotheca dysprosita | IUCN, 2024 |
| Amphibia | Anura | Gastrotheca espeletia | IUCN, 2024 |
| Amphibia | Anura | Gastrotheca excubitor | IUCN, 2024 |
| Amphibia | Anura | Gastrotheca galeata | IUCN, 2024 |
| Amphibia | Anura | Gastrotheca griswoldi | IUCN, 2024 |
| Amphibia | Anura | Gastrotheca lateonota | IUCN, 2024 |
| Amphibia | Anura | Gastrotheca lauzuricae | IUCN, 2024 |
| Amphibia | Anura | Gastrotheca litonedis | IUCN, 2024 |
| Amphibia | Anura | Gastrotheca lojana | IUCN, 2024 |
| Amphibia | Anura | Gastrotheca longipes | IUCN, 2024 |
| Amphibia | Anura | Gastrotheca marsupiata | IUCN, 2024 |
| Amphibia | Anura | Gastrotheca monticola | IUCN, 2024 |
| Amphibia | Anura | Gastrotheca nebulanastes | IUCN, 2024 |
| Amphibia | Anura | Gastrotheca nicefori | IUCN, 2024 |
| Amphibia | Anura | Gastrotheca ochoai | IUCN, 2024 |
| Amphibia | Anura | Gastrotheca oresbios | IUCN, 2024 |
| Amphibia | Anura | Gastrotheca orophylax | IUCN, 2024 |
| Amphibia | Anura | Gastrotheca ossilaginis | IUCN, 2024 |
| Amphibia | Anura | Gastrotheca pacchamama | IUCN, 2024 |
| Amphibia | Anura | Gastrotheca pachachacae | IUCN, 2024 |
| Amphibia | Anura | Gastrotheca peruana | IUCN, 2024 |
| Amphibia | Anura | Gastrotheca phalarosa | IUCN, 2024 |
| Amphibia | Anura | Gastrotheca phelloderma | IUCN, 2024 |
| Amphibia | Anura | Gastrotheca piperata | IUCN, 2024 |
| Amphibia | Anura | Gastrotheca plumbea | IUCN, 2024 |
| Amphibia | Anura | Gastrotheca pseustes | IUCN, 2024 |
| Amphibia | Anura | Gastrotheca psychrophila | IUCN, 2024 |
| Amphibia | Anura | Gastrotheca rebeccae | IUCN, 2024 |
| Amphibia | Anura | Gastrotheca riobambae | IUCN, 2024 |
| Amphibia | Anura | Gastrotheca ruizi | IUCN, 2024 |
| Amphibia | Anura | Gastrotheca spectabilis | IUCN, 2024 |
| Amphibia | Anura | Gastrotheca splendens | IUCN, 2024 |
| Amphibia | Anura | Gastrotheca stictopleura | IUCN, 2024 |
| Amphibia | Anura | Gastrotheca testudinea | IUCN, 2024 |
| Amphibia | Anura | Gastrotheca weinlandii | IUCN, 2024 |
| Amphibia | Anura | Gastrotheca zeugocystis | IUCN, 2024 |
| Amphibia | Anura | Hamptophryne alios | IUCN, 2024 |
| Amphibia | Anura | Hamptophryne boliviana | IUCN, 2024; Present study |
| Amphibia | Anura | Hemiphractus bubalus | IUCN, 2024 |
| Amphibia | Anura | Hemiphractus helioi | IUCN, 2024; Present study |
| Amphibia | Anura | Hemiphractus proboscideus | IUCN, 2024 |
| Amphibia | Anura | Hemiphractus scutatus | IUCN, 2024; Present study |
| Amphibia | Anura | Hyalinobatrachium anachoretus | IUCN, 2024 |
| Amphibia | Anura | Hyalinobatrachium bergeri | IUCN, 2024 |
| Amphibia | Anura | Hyalinobatrachium crurifasciatum | IUCN, 2024 |
| Amphibia | Anura | Hyalinobatrachium kawense | IUCN, 2024 |
| Amphibia | Anura | Hyalinobatrachium munozorum | IUCN, 2024 |
| Amphibia | Anura | Hyalinobatrachium pellucidum | IUCN, 2024 |
| Amphibia | Anura | Hyalinobatrachium ruedai | IUCN, 2024 |
| Amphibia | Anura | Hyalinobatrachium taylori | IUCN, 2024 |
| Amphibia | Anura | Hydrolaetare caparu | IUCN, 2024 |
| Amphibia | Anura | Hydrolaetare dantasi | IUCN, 2024 |
| Amphibia | Anura | Hydrolaetare schmidti | IUCN, 2024 |
| Amphibia | Anura | Hyla imitator | IUCN, 2024 |
| Amphibia | Anura | Hyloscirtus albopunctulatus | IUCN, 2024 |
| Amphibia | Anura | Hyloscirtus alytolylax | IUCN, 2024 |
| Amphibia | Anura | Hyloscirtus armatus | IUCN, 2024 |
| Amphibia | Anura | Hyloscirtus charazani | IUCN, 2024 |
| Amphibia | Anura | Hyloscirtus chlorosteus | IUCN, 2024 |
| Amphibia | Anura | Hyloscirtus diabolus | IUCN, 2024 |
| Amphibia | Anura | Hyloscirtus larinopygion | IUCN, 2024 |
| Amphibia | Anura | Hyloscirtus lindae | IUCN, 2024 |
| Amphibia | Anura | Hyloscirtus pacha | IUCN, 2024 |
| Amphibia | Anura | Hyloscirtus pantostictus | IUCN, 2024 |
| Amphibia | Anura | Hyloscirtus phyllognathus | IUCN, 2024 |
| Amphibia | Anura | Hyloscirtus psarolaimus | IUCN, 2024 |
| Amphibia | Anura | Hyloscirtus staufferorum | IUCN, 2024 |
| Amphibia | Anura | Hyloscirtus tapichalaca | IUCN, 2024 |
| Amphibia | Anura | Hyloscirtus tigrinus | IUCN, 2024 |
| Amphibia | Anura | Hyloscirtus torrenticola | IUCN, 2024 |
| Amphibia | Anura | Hyloxalus aeruginosus | IUCN, 2024 |
| Amphibia | Anura | Hyloxalus anthracinus | IUCN, 2024 |
| Amphibia | Anura | Hyloxalus azureiventris | IUCN, 2024 |
| Amphibia | Anura | Hyloxalus bocagei | IUCN, 2024 |
| Amphibia | Anura | Hyloxalus breviquartus | IUCN, 2024 |
| Amphibia | Anura | Hyloxalus cevallosi | IUCN, 2024 |
| Amphibia | Anura | Hyloxalus chlorocraspedus | IUCN, 2024 |
| Amphibia | Anura | Hyloxalus craspedoceps | IUCN, 2024 |
| Amphibia | Anura | Hyloxalus elachyhistus | IUCN, 2024 |
| Amphibia | Anura | Hyloxalus eleutherodactylus | IUCN, 2024 |
| Amphibia | Anura | Hyloxalus exasperatus | IUCN, 2024 |
| Amphibia | Anura | Hyloxalus faciopunctulatus | IUCN, 2024 |
| Amphibia | Anura | Hyloxalus fuliginosus | IUCN, 2024 |
| Amphibia | Anura | Hyloxalus idiomelus | IUCN, 2024 |
| Amphibia | Anura | Hyloxalus insulatus | IUCN, 2024 |
| Amphibia | Anura | Hyloxalus italoi | IUCN, 2024 |
| Amphibia | Anura | Hyloxalus leucophaeus | IUCN, 2024 |
| Amphibia | Anura | Hyloxalus littoralis | IUCN, 2024 |
| Amphibia | Anura | Hyloxalus maculosus | IUCN, 2024 |
| Amphibia | Anura | Hyloxalus marmoreoventris | IUCN, 2024 |
| Amphibia | Anura | Hyloxalus mittermeieri | IUCN, 2024 |
| Amphibia | Anura | Hyloxalus mystax | IUCN, 2024 |
| Amphibia | Anura | Hyloxalus nexipus | IUCN, 2024 |
| Amphibia | Anura | Hyloxalus parcus | IUCN, 2024 |
| Amphibia | Anura | Hyloxalus patitae | IUCN, 2024 |
| Amphibia | Anura | Hyloxalus peculiaris | IUCN, 2024 |
| Amphibia | Anura | Hyloxalus peruvianus | IUCN, 2024 |
| Amphibia | Anura | Hyloxalus pulchellus | IUCN, 2024 |
| Amphibia | Anura | Hyloxalus pulcherrimus | IUCN, 2024 |
| Amphibia | Anura | Hyloxalus pumilus | IUCN, 2024 |
| Amphibia | Anura | Hyloxalus saltuarius | IUCN, 2024 |
| Amphibia | Anura | Hyloxalus sauli | IUCN, 2024 |
| Amphibia | Anura | Hyloxalus shuar | IUCN, 2024 |
| Amphibia | Anura | Hyloxalus sordidatus | IUCN, 2024 |
| Amphibia | Anura | Hyloxalus spilotogaster | IUCN, 2024 |
| Amphibia | Anura | Hyloxalus sylvaticus | IUCN, 2024 |
| Amphibia | Anura | Hyloxalus utcubambensis | IUCN, 2024 |
| Amphibia | Anura | Hyloxalus vertebralis | IUCN, 2024 |
| Amphibia | Anura | Leptodactylus bolivianus | IUCN, 2024; Present study |
| Amphibia | Anura | Leptodactylus bufonius | IUCN, 2024 |
| Amphibia | Anura | Leptodactylus chaquensis | IUCN, 2024 |
| Amphibia | Anura | Leptodactylus colombiensis | IUCN, 2024 |
| Amphibia | Anura | Leptodactylus didymus | IUCN, 2024; Present study |
| Amphibia | Anura | Leptodactylus diedrus | IUCN, 2024; Present study |
| Amphibia | Anura | Leptodactylus discodactylus | IUCN, 2024; Present study |
| Amphibia | Anura | Leptodactylus elenae | IUCN, 2024 |
| Amphibia | Anura | Leptodactylus furnarius | IUCN, 2024 |
| Amphibia | Anura | Leptodactylus fuscus | IUCN, 2024; Present study |
| Amphibia | Anura | Leptodactylus gracilis | IUCN, 2024 |
| Amphibia | Anura | Leptodactylus griseigularis | IUCN, 2024 |
| Amphibia | Anura | Leptodactylus insularum | IUCN, 2024 |
| Amphibia | Anura | Leptodactylus knudseni | IUCN, 2024; Present study |
| Amphibia | Anura | Leptodactylus labrosus | IUCN, 2024 |
| Amphibia | Anura | Leptodactylus labyrinthicus | IUCN, 2024 |
| Amphibia | Anura | Leptodactylus laticeps | IUCN, 2024 |
| Amphibia | Anura | Leptodactylus latinasus | IUCN, 2024 |
| Amphibia | Anura | Leptodactylus latrans | IUCN, 2024 |
| Amphibia | Anura | Leptodactylus lauramiriamae | IUCN, 2024 |
| Amphibia | Anura | Leptodactylus leptodactyloides | IUCN, 2024; Present study |
| Amphibia | Anura | Leptodactylus lithonaetes | IUCN, 2024 |
| Amphibia | Anura | Leptodactylus longirostris | IUCN, 2024 |
| Amphibia | Anura | Leptodactylus myersi | IUCN, 2024 |
| Amphibia | Anura | Leptodactylus mystaceus | IUCN, 2024; Present study |
| Amphibia | Anura | Leptodactylus mystacinus | IUCN, 2024 |
| Amphibia | Anura | Leptodactylus pallidirostris | IUCN, 2024 |
| Amphibia | Anura | Leptodactylus paraensis | IUCN, 2024 |
| Amphibia | Anura | Leptodactylus pascoensis | IUCN, 2024 |
| Amphibia | Anura | Leptodactylus pentadactylus | IUCN, 2024; Present study |
| Amphibia | Anura | Leptodactylus petersii | IUCN, 2024; Present study |
| Amphibia | Anura | Leptodactylus podicipinus | IUCN, 2024 |
| Amphibia | Anura | Leptodactylus pustulatus | IUCN, 2024 |
| Amphibia | Anura | Leptodactylus rhodomystax | IUCN, 2024; Present study |
| Amphibia | Anura | Leptodactylus rhodonotus | IUCN, 2024; Present study |
| Amphibia | Anura | Leptodactylus riveroi | IUCN, 2024 |
| Amphibia | Anura | Leptodactylus rugosus | IUCN, 2024 |
| Amphibia | Anura | Leptodactylus sabanensis | IUCN, 2024 |
| Amphibia | Anura | Leptodactylus stenodema | IUCN, 2024; Present study |
| Amphibia | Anura | Leptodactylus syphax | IUCN, 2024 |
| Amphibia | Anura | Leptodactylus wagneri | IUCN, 2024; Present study |
| Amphibia | Anura | Leucostethus argyrogaster | IUCN, 2024 |
| Amphibia | Anura | Leucostethus fugax | IUCN, 2024 |
| Amphibia | Anura | Lithobates catesbeianus | IUCN, 2024 |
| Amphibia | Anura | Lithobates palmipes | IUCN, 2024; Present study |
| Amphibia | Anura | Lithodytes lineatus | IUCN, 2024; Present study |
| Amphibia | Anura | Lynchius flavomaculatus | IUCN, 2024 |
| Amphibia | Anura | Lynchius nebulanastes | IUCN, 2024 |
| Amphibia | Anura | Lynchius oblitus | IUCN, 2024 |
| Amphibia | Anura | Lynchius parkeri | IUCN, 2024 |
| Amphibia | Anura | Lynchius simmonsi | IUCN, 2024 |
| Amphibia | Anura | Lynchius tabaconas | IUCN, 2024 |
| Amphibia | Anura | Lysapsus bolivianus | IUCN, 2024 |
| Amphibia | Anura | Lysapsus caraya | IUCN, 2024 |
| Amphibia | Anura | Lysapsus laevis | IUCN, 2024 |
| Amphibia | Anura | Lysapsus limellum | IUCN, 2024 |
| Amphibia | Anura | Melanophryniscus rubriventris | IUCN, 2024 |
| Amphibia | Anura | Microkayla adenopleura | IUCN, 2024 |
| Amphibia | Anura | Microkayla ankohuma | IUCN, 2024 |
| Amphibia | Anura | Microkayla boettgeri | IUCN, 2024 |
| Amphibia | Anura | Microkayla chacaltaya | IUCN, 2024 |
| Amphibia | Anura | Microkayla chaupi | IUCN, 2024 |
| Amphibia | Anura | Microkayla colla | IUCN, 2024 |
| Amphibia | Anura | Microkayla condoriri | IUCN, 2024 |
| Amphibia | Anura | Microkayla guillei | IUCN, 2024 |
| Amphibia | Anura | Microkayla harveyi | IUCN, 2024 |
| Amphibia | Anura | Microkayla huayna | IUCN, 2024 |
| Amphibia | Anura | Microkayla iani | IUCN, 2024 |
| Amphibia | Anura | Microkayla iatamasi | IUCN, 2024 |
| Amphibia | Anura | Microkayla illampu | IUCN, 2024 |
| Amphibia | Anura | Microkayla illimani | IUCN, 2024 |
| Amphibia | Anura | Microkayla kallawaya | IUCN, 2024 |
| Amphibia | Anura | Microkayla katantika | IUCN, 2024 |
| Amphibia | Anura | Microkayla kempffi | IUCN, 2024 |
| Amphibia | Anura | Microkayla melanocheira | IUCN, 2024 |
| Amphibia | Anura | Microkayla pinguis | IUCN, 2024 |
| Amphibia | Anura | Microkayla quimsacruzis | IUCN, 2024 |
| Amphibia | Anura | Microkayla saltator | IUCN, 2024 |
| Amphibia | Anura | Microkayla teqta | IUCN, 2024 |
| Amphibia | Anura | Microkayla wettsteini | IUCN, 2024 |
| Amphibia | Anura | Myersiohyla neblinaria | IUCN, 2024 |
| Amphibia | Anura | Nannophryne apolobambica | IUCN, 2024 |
| Amphibia | Anura | Nannophryne cophotis | IUCN, 2024 |
| Amphibia | Anura | Nannophryne corynetes | IUCN, 2024 |
| Amphibia | Anura | Nesorohyla kanaima | IUCN, 2024 |
| Amphibia | Anura | Niceforonia araiodactyla | IUCN, 2024 |
| Amphibia | Anura | Niceforonia brunnea | IUCN, 2024 |
| Amphibia | Anura | Niceforonia dolops | IUCN, 2024 |
| Amphibia | Anura | Niceforonia elassodiscus | IUCN, 2024 |
| Amphibia | Anura | Niceforonia fallaciosa | IUCN, 2024 |
| Amphibia | Anura | Niceforonia lucida | IUCN, 2024 |
| Amphibia | Anura | Niceforonia nigrovittata | IUCN, 2024; Present study |
| Amphibia | Anura | Niceforonia peraccai | IUCN, 2024 |
| Amphibia | Anura | Noblella carrascoicola | IUCN, 2024 |
| Amphibia | Anura | Noblella duellmani | IUCN, 2024 |
| Amphibia | Anura | Noblella heyeri | IUCN, 2024 |
| Amphibia | Anura | Noblella lochites | IUCN, 2024 |
| Amphibia | Anura | Noblella lynchi | IUCN, 2024 |
| Amphibia | Anura | Noblella madreselva | IUCN, 2024 |
| Amphibia | Anura | Noblella myrmecoides | IUCN, 2024; Present study |
| Amphibia | Anura | Noblella personina | IUCN, 2024 |
| Amphibia | Anura | Noblella peruviana | IUCN, 2024 |
| Amphibia | Anura | Noblella pygmaea | IUCN, 2024 |
| Amphibia | Anura | Noblella ritarasquinae | IUCN, 2024 |
| Amphibia | Anura | Nyctimantis rugiceps | IUCN, 2024; Present study |
| Amphibia | Anura | Nymphargus anomalus | IUCN, 2024 |
| Amphibia | Anura | Nymphargus bejaranoi | IUCN, 2024 |
| Amphibia | Anura | Nymphargus cariticommatus | IUCN, 2024 |
| Amphibia | Anura | Nymphargus chancas | IUCN, 2024 |
| Amphibia | Anura | Nymphargus cochranae | IUCN, 2024 |
| Amphibia | Anura | Nymphargus garciae | IUCN, 2024 |
| Amphibia | Anura | Nymphargus laurae | IUCN, 2024 |
| Amphibia | Anura | Nymphargus mariae | IUCN, 2024 |
| Amphibia | Anura | Nymphargus megacheirus | IUCN, 2024 |
| Amphibia | Anura | Nymphargus mixomaculatus | IUCN, 2024 |
| Amphibia | Anura | Nymphargus nephelophila | IUCN, 2024 |
| Amphibia | Anura | Nymphargus ocellatus | IUCN, 2024 |
| Amphibia | Anura | Nymphargus oreonympha | IUCN, 2024 |
| Amphibia | Anura | Nymphargus phenax | IUCN, 2024 |
| Amphibia | Anura | Nymphargus pluvialis | IUCN, 2024 |
| Amphibia | Anura | Nymphargus posadae | IUCN, 2024 |
| Amphibia | Anura | Nymphargus siren | IUCN, 2024 |
| Amphibia | Anura | Nymphargus truebae | IUCN, 2024 |
| Amphibia | Anura | Nymphargus wileyi | IUCN, 2024 |
| Amphibia | Anura | Odontophrynus lavillai | IUCN, 2024 |
| Amphibia | Anura | Oreobates amarakaeri | IUCN, 2024 |
| Amphibia | Anura | Oreobates ayacucho | IUCN, 2024 |
| Amphibia | Anura | Oreobates choristolemma | IUCN, 2024 |
| Amphibia | Anura | Oreobates cruralis | IUCN, 2024 |
| Amphibia | Anura | Oreobates discoidalis | IUCN, 2024 |
| Amphibia | Anura | Oreobates gemcare | IUCN, 2024 |
| Amphibia | Anura | Oreobates granulosus | IUCN, 2024 |
| Amphibia | Anura | Oreobates ibischi | IUCN, 2024 |
| Amphibia | Anura | Oreobates lehri | IUCN, 2024 |
| Amphibia | Anura | Oreobates lundbergi | IUCN, 2024 |
| Amphibia | Anura | Oreobates machiguenga | IUCN, 2024; Present study |
| Amphibia | Anura | Oreobates madidi | IUCN, 2024 |
| Amphibia | Anura | Oreobates pereger | IUCN, 2024 |
| Amphibia | Anura | Oreobates quixensis | IUCN, 2024; Present study |
| Amphibia | Anura | Oreobates sanctaecrucis | IUCN, 2024 |
| Amphibia | Anura | Oreobates sanderi | IUCN, 2024 |
| Amphibia | Anura | Oreobates saxatilis | IUCN, 2024 |
| Amphibia | Anura | Oreobates yanucu | IUCN, 2024 |
| Amphibia | Anura | Oreobates zongoensis | IUCN, 2024 |
| Amphibia | Anura | Oreophrynella macconnelli | IUCN, 2024 |
| Amphibia | Anura | Osornophryne antisana | IUCN, 2024 |
| Amphibia | Anura | Osornophryne bufoniformis | IUCN, 2024 |
| Amphibia | Anura | Osornophryne cofanorum | IUCN, 2024 |
| Amphibia | Anura | Osornophryne guacamayo | IUCN, 2024 |
| Amphibia | Anura | Osornophryne puruanta | IUCN, 2024 |
| Amphibia | Anura | Osornophryne sumacoensis | IUCN, 2024 |
| Amphibia | Anura | Osornophryne talipes | IUCN, 2024 |
| Amphibia | Anura | Osteocephalus alboguttatus | IUCN, 2024 |
| Amphibia | Anura | Osteocephalus buckleyi | IUCN, 2024; Present study |
| Amphibia | Anura | Osteocephalus cabrerai | IUCN, 2024; Present study |
| Amphibia | Anura | Osteocephalus castaneicola | IUCN, 2024; Present study |
| Amphibia | Anura | Osteocephalus deridens | IUCN, 2024; Present study |
| Amphibia | Anura | Osteocephalus elkejungingerae | IUCN, 2024 |
| Amphibia | Anura | Osteocephalus fuscifacies | IUCN, 2024 |
| Amphibia | Anura | Osteocephalus heyeri | IUCN, 2024 |
| Amphibia | Anura | Osteocephalus leoniae | IUCN, 2024 |
| Amphibia | Anura | Osteocephalus leprieurii | IUCN, 2024 |
| Amphibia | Anura | Osteocephalus mutabor | IUCN, 2024; Present study |
| Amphibia | Anura | Osteocephalus oophagus | IUCN, 2024 |
| Amphibia | Anura | Osteocephalus planiceps | IUCN, 2024; Present study |
| Amphibia | Anura | Osteocephalus subtilis | IUCN, 2024; Present study |
| Amphibia | Anura | Osteocephalus taurinus | IUCN, 2024; Present study |
| Amphibia | Anura | Osteocephalus verruciger | IUCN, 2024 |
| Amphibia | Anura | Osteocephalus yasuni | IUCN, 2024; Present study |
| Amphibia | Anura | Otophryne pyburni | IUCN, 2024 |
| Amphibia | Anura | Otophryne robusta | IUCN, 2024 |
| Amphibia | Anura | Otophryne steyermarki | IUCN, 2024 |
| Amphibia | Anura | Phrynopus anancites | IUCN, 2024 |
| Amphibia | Anura | Phrynopus auriculatus | IUCN, 2024 |
| Amphibia | Anura | Phrynopus badius | IUCN, 2024 |
| Amphibia | Anura | Phrynopus barthlenae | IUCN, 2024 |
| Amphibia | Anura | Phrynopus bracki | IUCN, 2024 |
| Amphibia | Anura | Phrynopus bufoides | IUCN, 2024 |
| Amphibia | Anura | Phrynopus capitalis | IUCN, 2024 |
| Amphibia | Anura | Phrynopus chaparroi | IUCN, 2024 |
| Amphibia | Anura | Phrynopus curator | IUCN, 2024 |
| Amphibia | Anura | Phrynopus daemon | IUCN, 2024 |
| Amphibia | Anura | Phrynopus dagmarae | IUCN, 2024 |
| Amphibia | Anura | Phrynopus dumicola | IUCN, 2024 |
| Amphibia | Anura | Phrynopus heimorum | IUCN, 2024 |
| Amphibia | Anura | Phrynopus horstpauli | IUCN, 2024 |
| Amphibia | Anura | Phrynopus interstinctus | IUCN, 2024 |
| Amphibia | Anura | Phrynopus inti | IUCN, 2024 |
| Amphibia | Anura | Phrynopus juninensis | IUCN, 2024 |
| Amphibia | Anura | Phrynopus kauneorum | IUCN, 2024 |
| Amphibia | Anura | Phrynopus kotosh | IUCN, 2024 |
| Amphibia | Anura | Phrynopus lapidoides | IUCN, 2024 |
| Amphibia | Anura | Phrynopus lechriorhynchus | IUCN, 2024 |
| Amphibia | Anura | Phrynopus miroslawae | IUCN, 2024 |
| Amphibia | Anura | Phrynopus montium | IUCN, 2024 |
| Amphibia | Anura | Phrynopus nicoleae | IUCN, 2024 |
| Amphibia | Anura | Phrynopus oblivius | IUCN, 2024 |
| Amphibia | Anura | Phrynopus paucari | IUCN, 2024 |
| Amphibia | Anura | Phrynopus personatus | IUCN, 2024 |
| Amphibia | Anura | Phrynopus peruanus | IUCN, 2024 |
| Amphibia | Anura | Phrynopus pesantesi | IUCN, 2024 |
| Amphibia | Anura | Phrynopus tautzorum | IUCN, 2024 |
| Amphibia | Anura | Phrynopus tribulosus | IUCN, 2024 |
| Amphibia | Anura | Phrynopus unchog | IUCN, 2024 |
| Amphibia | Anura | Phrynopus valquii | IUCN, 2024 |
| Amphibia | Anura | Phrynopus vestigiatus | IUCN, 2024 |
| Amphibia | Anura | Phyllomedusa bicolor | IUCN, 2024; Present study |
| Amphibia | Anura | Phyllomedusa boliviana | IUCN, 2024 |
| Amphibia | Anura | Phyllomedusa camba | IUCN, 2024; Present study |
| Amphibia | Anura | Phyllomedusa coelestis | IUCN, 2024 |
| Amphibia | Anura | Phyllomedusa sauvagii | IUCN, 2024 |
| Amphibia | Anura | Phyllomedusa tarsius | IUCN, 2024; Present study |
| Amphibia | Anura | Phyllomedusa vaillantii | IUCN, 2024; Present study |
| Amphibia | Anura | Physalaemus albonotatus | IUCN, 2024 |
| Amphibia | Anura | Physalaemus biligonigerus | IUCN, 2024 |
| Amphibia | Anura | Physalaemus centralis | IUCN, 2024 |
| Amphibia | Anura | Physalaemus cuvieri | IUCN, 2024; Present study |
| Amphibia | Anura | Physalaemus ephippifer | IUCN, 2024 |
| Amphibia | Anura | Physalaemus marmoratus | IUCN, 2024 |
| Amphibia | Anura | Physalaemus nattereri | IUCN, 2024 |
| Amphibia | Anura | Phyzelaphryne miriamae | IUCN, 2024 |
| Amphibia | Anura | Pipa arrabali | IUCN, 2024 |
| Amphibia | Anura | Pipa aspera | IUCN, 2024 |
| Amphibia | Anura | Pipa pipa | IUCN, 2024; Present study |
| Amphibia | Anura | Pipa snethlageae | IUCN, 2024 |
| Amphibia | Anura | Pithecopus azureus | IUCN, 2024 |
| Amphibia | Anura | Pithecopus hypochondrialis | IUCN, 2024; Present study |
| Amphibia | Anura | Pithecopus palliatus | IUCN, 2024 |
| Amphibia | Anura | Pleurodema brachyops | IUCN, 2024 |
| Amphibia | Anura | Pleurodema cinereum | IUCN, 2024 |
| Amphibia | Anura | Pleurodema marmoratum | IUCN, 2024 |
| Amphibia | Anura | Pristimantis aaptus | IUCN, 2024 |
| Amphibia | Anura | Pristimantis acerus | IUCN, 2024 |
| Amphibia | Anura | Pristimantis achuar | IUCN, 2024 |
| Amphibia | Anura | Pristimantis acuminatus | IUCN, 2024; Present study |
| Amphibia | Anura | Pristimantis adiastolus | IUCN, 2024 |
| Amphibia | Anura | Pristimantis albertus | IUCN, 2024 |
| Amphibia | Anura | Pristimantis altamazonicus | IUCN, 2024; Present study |
| Amphibia | Anura | Pristimantis altamnis | IUCN, 2024 |
| Amphibia | Anura | Pristimantis anemerus | IUCN, 2024 |
| Amphibia | Anura | Pristimantis aniptopalmatus | IUCN, 2024 |
| Amphibia | Anura | Pristimantis aquilonaris | IUCN, 2024 |
| Amphibia | Anura | Pristimantis ardalonychus | IUCN, 2024 |
| Amphibia | Anura | Pristimantis ashaninka | IUCN, 2024 |
| Amphibia | Anura | Pristimantis atrabracus | IUCN, 2024 |
| Amphibia | Anura | Pristimantis atratus | IUCN, 2024 |
| Amphibia | Anura | Pristimantis attenboroughi | IUCN, 2024 |
| Amphibia | Anura | Pristimantis aureolineatus | IUCN, 2024 |
| Amphibia | Anura | Pristimantis avicuporum | IUCN, 2024 |
| Amphibia | Anura | Pristimantis avius | IUCN, 2024 |
| Amphibia | Anura | Pristimantis balionotus | IUCN, 2024 |
| Amphibia | Anura | Pristimantis bambu | IUCN, 2024 |
| Amphibia | Anura | Pristimantis baryecuus | IUCN, 2024 |
| Amphibia | Anura | Pristimantis bearsei | IUCN, 2024 |
| Amphibia | Anura | Pristimantis bellator | IUCN, 2024 |
| Amphibia | Anura | Pristimantis bipunctatus | IUCN, 2024 |
| Amphibia | Anura | Pristimantis boucephalus | IUCN, 2024 |
| Amphibia | Anura | Pristimantis bounides | IUCN, 2024 |
| Amphibia | Anura | Pristimantis bromeliaceus | IUCN, 2024 |
| Amphibia | Anura | Pristimantis buccinator | IUCN, 2024; Present study |
| Amphibia | Anura | Pristimantis buckleyi | IUCN, 2024 |
| Amphibia | Anura | Pristimantis bustamante | IUCN, 2024 |
| Amphibia | Anura | Pristimantis caeruleonotus | IUCN, 2024 |
| Amphibia | Anura | Pristimantis cajamarcensis | IUCN, 2024 |
| Amphibia | Anura | Pristimantis carvalhoi | IUCN, 2024; Present study |
| Amphibia | Anura | Pristimantis ceuthospilus | IUCN, 2024 |
| Amphibia | Anura | Pristimantis chiastonotus | IUCN, 2024 |
| Amphibia | Anura | Pristimantis chloronotus | IUCN, 2024 |
| Amphibia | Anura | Pristimantis citriogaster | IUCN, 2024 |
| Amphibia | Anura | Pristimantis colodactylus | IUCN, 2024 |
| Amphibia | Anura | Pristimantis colonensis | IUCN, 2024 |
| Amphibia | Anura | Pristimantis condor | IUCN, 2024 |
| Amphibia | Anura | Pristimantis conspicillatus | IUCN, 2024; Present study |
| Amphibia | Anura | Pristimantis cordovae | IUCN, 2024 |
| Amphibia | Anura | Pristimantis corniger | IUCN, 2024 |
| Amphibia | Anura | Pristimantis coronatus | IUCN, 2024 |
| Amphibia | Anura | Pristimantis corrugatus | IUCN, 2024 |
| Amphibia | Anura | Pristimantis cosnipatae | IUCN, 2024 |
| Amphibia | Anura | Pristimantis cremnobates | IUCN, 2024 |
| Amphibia | Anura | Pristimantis croceoinguinis | IUCN, 2024; Present study |
| Amphibia | Anura | Pristimantis cruciocularis | IUCN, 2024 |
| Amphibia | Anura | Pristimantis cryophilius | IUCN, 2024 |
| Amphibia | Anura | Pristimantis cryptomelas | IUCN, 2024 |
| Amphibia | Anura | Pristimantis cuneirostris | IUCN, 2024 |
| Amphibia | Anura | Pristimantis curtipes | IUCN, 2024 |
| Amphibia | Anura | Pristimantis danae | IUCN, 2024 |
| Amphibia | Anura | Pristimantis delius | IUCN, 2024; Present study |
| Amphibia | Anura | Pristimantis dendrobatoides | IUCN, 2024 |
| Amphibia | Anura | Pristimantis devillei | IUCN, 2024 |
| Amphibia | Anura | Pristimantis deyi | IUCN, 2024 |
| Amphibia | Anura | Pristimantis diadematus | IUCN, 2024; Present study |
| Amphibia | Anura | Pristimantis divnae | IUCN, 2024 |
| Amphibia | Anura | Pristimantis dundeei | IUCN, 2024 |
| Amphibia | Anura | Pristimantis enigmaticus | IUCN, 2024 |
| Amphibia | Anura | Pristimantis epacrus | IUCN, 2024 |
| Amphibia | Anura | Pristimantis eriphus | IUCN, 2024 |
| Amphibia | Anura | Pristimantis ernesti | IUCN, 2024 |
| Amphibia | Anura | Pristimantis eurydactylus | IUCN, 2024 |
| Amphibia | Anura | Pristimantis exoristus | IUCN, 2024 |
| Amphibia | Anura | Pristimantis farisorum | IUCN, 2024 |
| Amphibia | Anura | Pristimantis fenestratus | IUCN, 2024; Present study |
| Amphibia | Anura | Pristimantis festae | IUCN, 2024 |
| Amphibia | Anura | Pristimantis galdi | IUCN, 2024 |
| Amphibia | Anura | Pristimantis ganonotus | IUCN, 2024 |
| Amphibia | Anura | Pristimantis gladiator | IUCN, 2024 |
| Amphibia | Anura | Pristimantis glandulosus | IUCN, 2024 |
| Amphibia | Anura | Pristimantis gutturalis | IUCN, 2024 |
| Amphibia | Anura | Pristimantis hernandezi | IUCN, 2024 |
| Amphibia | Anura | Pristimantis huicundo | IUCN, 2024 |
| Amphibia | Anura | Pristimantis humboldti | IUCN, 2024 |
| Amphibia | Anura | Pristimantis ignicolor | IUCN, 2024 |
| Amphibia | Anura | Pristimantis iiap | IUCN, 2024 |
| Amphibia | Anura | Pristimantis imitatrix | IUCN, 2024 |
| Amphibia | Anura | Pristimantis incanus | IUCN, 2024 |
| Amphibia | Anura | Pristimantis incomptus | IUCN, 2024 |
| Amphibia | Anura | Pristimantis infraguttatus | IUCN, 2024 |
| Amphibia | Anura | Pristimantis inguinalis | IUCN, 2024 |
| Amphibia | Anura | Pristimantis inusitatus | IUCN, 2024 |
| Amphibia | Anura | Pristimantis karcharias | IUCN, 2024 |
| Amphibia | Anura | Pristimantis katoptroides | IUCN, 2024 |
| Amphibia | Anura | Pristimantis kichwarum | IUCN, 2024 |
| Amphibia | Anura | Pristimantis kirklandi | IUCN, 2024 |
| Amphibia | Anura | Pristimantis lacrimosus | IUCN, 2024 |
| Amphibia | Anura | Pristimantis lanthanites | IUCN, 2024; Present study |
| Amphibia | Anura | Pristimantis leoni | IUCN, 2024 |
| Amphibia | Anura | Pristimantis leucopus | IUCN, 2024 |
| Amphibia | Anura | Pristimantis leucorrhinus | IUCN, 2024 |
| Amphibia | Anura | Pristimantis librarius | IUCN, 2024 |
| Amphibia | Anura | Pristimantis limoncochensis | IUCN, 2024 |
| Amphibia | Anura | Pristimantis lindae | IUCN, 2024 |
| Amphibia | Anura | Pristimantis lirellus | IUCN, 2024 |
| Amphibia | Anura | Pristimantis lividus | IUCN, 2024 |
| Amphibia | Anura | Pristimantis llojsintuta | IUCN, 2024 |
| Amphibia | Anura | Pristimantis lucasi | IUCN, 2024 |
| Amphibia | Anura | Pristimantis luscombei | IUCN, 2024; Present study |
| Amphibia | Anura | Pristimantis lymani | IUCN, 2024 |
| Amphibia | Anura | Pristimantis lythrodes | IUCN, 2024 |
| Amphibia | Anura | Pristimantis malkini | IUCN, 2024; Present study |
| Amphibia | Anura | Pristimantis mariaelenae | IUCN, 2024 |
| Amphibia | Anura | Pristimantis marmoratus | IUCN, 2024 |
| Amphibia | Anura | Pristimantis martiae | IUCN, 2024; Present study |
| Amphibia | Anura | Pristimantis medemi | IUCN, 2024 |
| Amphibia | Anura | Pristimantis melanogaster | IUCN, 2024 |
| Amphibia | Anura | Pristimantis memorans | IUCN, 2024 |
| Amphibia | Anura | Pristimantis mendax | IUCN, 2024 |
| Amphibia | Anura | Pristimantis metabates | IUCN, 2024 |
| Amphibia | Anura | Pristimantis minutulus | IUCN, 2024 |
| Amphibia | Anura | Pristimantis modipeplus | IUCN, 2024 |
| Amphibia | Anura | Pristimantis muscosus | IUCN, 2024 |
| Amphibia | Anura | Pristimantis myersi | IUCN, 2024 |
| Amphibia | Anura | Pristimantis nebulosus | IUCN, 2024 |
| Amphibia | Anura | Pristimantis nephophilus | IUCN, 2024 |
| Amphibia | Anura | Pristimantis nigrogriseus | IUCN, 2024 |
| Amphibia | Anura | Pristimantis ockendeni | IUCN, 2024; Present study |
| Amphibia | Anura | Pristimantis olivaceus | IUCN, 2024 |
| Amphibia | Anura | Pristimantis omeviridis | IUCN, 2024 |
| Amphibia | Anura | Pristimantis orcesi | IUCN, 2024 |
| Amphibia | Anura | Pristimantis orcus | IUCN, 2024; Present study |
| Amphibia | Anura | Pristimantis orestes | IUCN, 2024 |
| Amphibia | Anura | Pristimantis ornatus | IUCN, 2024 |
| Amphibia | Anura | Pristimantis orphnolaimus | IUCN, 2024 |
| Amphibia | Anura | Pristimantis padiali | IUCN, 2024; Present study |
| Amphibia | Anura | Pristimantis pardalinus | IUCN, 2024 |
| Amphibia | Anura | Pristimantis pastazensis | IUCN, 2024 |
| Amphibia | Anura | Pristimantis pataikos | IUCN, 2024 |
| Amphibia | Anura | Pristimantis paululus | IUCN, 2024 |
| Amphibia | Anura | Pristimantis pecki | IUCN, 2024 |
| Amphibia | Anura | Pristimantis percnopterus | IUCN, 2024 |
| Amphibia | Anura | Pristimantis percultus | IUCN, 2024 |
| Amphibia | Anura | Pristimantis peruvianus | IUCN, 2024; Present study |
| Amphibia | Anura | Pristimantis petersi | IUCN, 2024 |
| Amphibia | Anura | Pristimantis petrobardus | IUCN, 2024 |
| Amphibia | Anura | Pristimantis pharangobates | IUCN, 2024 |
| Amphibia | Anura | Pristimantis philipi | IUCN, 2024 |
| Amphibia | Anura | Pristimantis phoxocephalus | IUCN, 2024 |
| Amphibia | Anura | Pristimantis pinguis | IUCN, 2024 |
| Amphibia | Anura | Pristimantis platydactylus | IUCN, 2024 |
| Amphibia | Anura | Pristimantis pluvialis | IUCN, 2024 |
| Amphibia | Anura | Pristimantis prolatus | IUCN, 2024 |
| Amphibia | Anura | Pristimantis proserpens | IUCN, 2024 |
| Amphibia | Anura | Pristimantis pseudoacuminatus | IUCN, 2024 |
| Amphibia | Anura | Pristimantis pugnax | IUCN, 2024 |
| Amphibia | Anura | Pristimantis puipui | IUCN, 2024 |
| Amphibia | Anura | Pristimantis pulchridormientes | IUCN, 2024 |
| Amphibia | Anura | Pristimantis pulvinatus | IUCN, 2024 |
| Amphibia | Anura | Pristimantis pycnodermis | IUCN, 2024 |
| Amphibia | Anura | Pristimantis quaquaversus | IUCN, 2024 |
| Amphibia | Anura | Pristimantis repens | IUCN, 2024 |
| Amphibia | Anura | Pristimantis rhabdocnemus | IUCN, 2024 |
| Amphibia | Anura | Pristimantis rhabdolaemus | IUCN, 2024 |
| Amphibia | Anura | Pristimantis rhodoplichus | IUCN, 2024 |
| Amphibia | Anura | Pristimantis rhodostichus | IUCN, 2024 |
| Amphibia | Anura | Pristimantis riveti | IUCN, 2024 |
| Amphibia | Anura | Pristimantis royi | IUCN, 2024 |
| Amphibia | Anura | Pristimantis rubicundus | IUCN, 2024 |
| Amphibia | Anura | Pristimantis rufioculis | IUCN, 2024 |
| Amphibia | Anura | Pristimantis sagittulus | IUCN, 2024 |
| Amphibia | Anura | Pristimantis salaputium | IUCN, 2024 |
| Amphibia | Anura | Pristimantis samaipatae | IUCN, 2024 |
| Amphibia | Anura | Pristimantis schultei | IUCN, 2024 |
| Amphibia | Anura | Pristimantis scitulus | IUCN, 2024 |
| Amphibia | Anura | Pristimantis seorsus | IUCN, 2024 |
| Amphibia | Anura | Pristimantis serendipitus | IUCN, 2024 |
| Amphibia | Anura | Pristimantis simonsii | IUCN, 2024 |
| Amphibia | Anura | Pristimantis skydmainos | IUCN, 2024; Present study |
| Amphibia | Anura | Pristimantis spectabilis | IUCN, 2024 |
| Amphibia | Anura | Pristimantis spinosus | IUCN, 2024 |
| Amphibia | Anura | Pristimantis sternothylax | IUCN, 2024 |
| Amphibia | Anura | Pristimantis stictoboubonus | IUCN, 2024 |
| Amphibia | Anura | Pristimantis stictogaster | IUCN, 2024 |
| Amphibia | Anura | Pristimantis stipa | IUCN, 2024 |
| Amphibia | Anura | Pristimantis supernatis | IUCN, 2024 |
| Amphibia | Anura | Pristimantis tamsitti | IUCN, 2024 |
| Amphibia | Anura | Pristimantis tantanti | IUCN, 2024 |
| Amphibia | Anura | Pristimantis tanyrhynchus | IUCN, 2024 |
| Amphibia | Anura | Pristimantis thymelensis | IUCN, 2024 |
| Amphibia | Anura | Pristimantis toftae | IUCN, 2024 |
| Amphibia | Anura | Pristimantis trachyblepharis | IUCN, 2024 |
| Amphibia | Anura | Pristimantis truebae | IUCN, 2024 |
| Amphibia | Anura | Pristimantis unistrigatus | IUCN, 2024 |
| Amphibia | Anura | Pristimantis variabilis | IUCN, 2024 |
| Amphibia | Anura | Pristimantis ventrimarmoratus | IUCN, 2024; Present study |
| Amphibia | Anura | Pristimantis versicolor | IUCN, 2024 |
| Amphibia | Anura | Pristimantis vicarius | IUCN, 2024 |
| Amphibia | Anura | Pristimantis vidua | IUCN, 2024 |
| Amphibia | Anura | Pristimantis vilarsi | IUCN, 2024 |
| Amphibia | Anura | Pristimantis vilcabambae | IUCN, 2024 |
| Amphibia | Anura | Pristimantis wagteri | IUCN, 2024 |
| Amphibia | Anura | Pristimantis waoranii | IUCN, 2024 |
| Amphibia | Anura | Pristimantis w-nigrum | IUCN, 2024 |
| Amphibia | Anura | Pristimantis zeuctotylus | IUCN, 2024 |
| Amphibia | Anura | Pristimantis zimmermanae | IUCN, 2024; Present study |
| Amphibia | Anura | Pristimantis zoilae | IUCN, 2024 |
| Amphibia | Anura | Proceratophrys concavitympanum | IUCN, 2024 |
| Amphibia | Anura | Pseudis paradoxa | IUCN, 2024; Present study |
| Amphibia | Anura | Pseudopaludicola boliviana | IUCN, 2024 |
| Amphibia | Anura | Pseudopaludicola canga | IUCN, 2024 |
| Amphibia | Anura | Pseudopaludicola ceratophyes | IUCN, 2024; Present study |
| Amphibia | Anura | Pseudopaludicola mystacalis | IUCN, 2024 |
| Amphibia | Anura | Psychrophrynella bagrecito | IUCN, 2024 |
| Amphibia | Anura | Psychrophrynella chirihampatu | IUCN, 2024 |
| Amphibia | Anura | Psychrophrynella usurpator | IUCN, 2024 |
| Amphibia | Anura | Ranitomeya amazonica | IUCN, 2024; Present study |
| Amphibia | Anura | Ranitomeya benedicta | IUCN, 2024 |
| Amphibia | Anura | Ranitomeya defleri | IUCN, 2024 |
| Amphibia | Anura | Ranitomeya fantastica | IUCN, 2024 |
| Amphibia | Anura | Ranitomeya flavovittata | IUCN, 2024 |
| Amphibia | Anura | Ranitomeya imitator | IUCN, 2024 |
| Amphibia | Anura | Ranitomeya reticulata | IUCN, 2024; Present study |
| Amphibia | Anura | Ranitomeya sirensis | IUCN, 2024 |
| Amphibia | Anura | Ranitomeya summersi | IUCN, 2024 |
| Amphibia | Anura | Ranitomeya uakarii | IUCN, 2024; Present study |
| Amphibia | Anura | Ranitomeya vanzolinii | IUCN, 2024 |
| Amphibia | Anura | Ranitomeya variabilis | IUCN, 2024; Present study |
| Amphibia | Anura | Ranitomeya ventrimaculata | IUCN, 2024; Present study |
| Amphibia | Anura | Ranitomeya yavaricola | IUCN, 2024 |
| Amphibia | Anura | Rhaebo glaberrimus | IUCN, 2024; Present study |
| Amphibia | Anura | Rhaebo guttatus | IUCN, 2024; Present study |
| Amphibia | Anura | Rhaebo nasicus | IUCN, 2024 |
| Amphibia | Anura | Rhinella acutirostris | IUCN, 2024 |
| Amphibia | Anura | Rhinella amabilis | IUCN, 2024 |
| Amphibia | Anura | Rhinella amboroensis | IUCN, 2024 |
| Amphibia | Anura | Rhinella arborescandens | IUCN, 2024 |
| Amphibia | Anura | Rhinella arenarum | IUCN, 2024 |
| Amphibia | Anura | Rhinella castaneotica | IUCN, 2024; Present study |
| Amphibia | Anura | Rhinella ceratophrys | IUCN, 2024; Present study |
| Amphibia | Anura | Rhinella chavin | IUCN, 2024 |
| Amphibia | Anura | Rhinella cristinae | IUCN, 2024 |
| Amphibia | Anura | Rhinella dapsilis | IUCN, 2024; Present study |
| Amphibia | Anura | Rhinella festae | IUCN, 2024 |
| Amphibia | Anura | Rhinella fissipes | IUCN, 2024 |
| Amphibia | Anura | Rhinella granulosa | IUCN, 2024 |
| Amphibia | Anura | Rhinella inca | IUCN, 2024 |
| Amphibia | Anura | Rhinella iserni | IUCN, 2024 |
| Amphibia | Anura | Rhinella justinianoi | IUCN, 2024 |
| Amphibia | Anura | Rhinella leptoscelis | IUCN, 2024 |
| Amphibia | Anura | Rhinella lescurei | IUCN, 2024 |
| Amphibia | Anura | Rhinella magnussoni | IUCN, 2024 |
| Amphibia | Anura | Rhinella manu | IUCN, 2024 |
| Amphibia | Anura | Rhinella margaritifera | IUCN, 2024; Present study |
| Amphibia | Anura | Rhinella marina | IUCN, 2024; Present study |
| Amphibia | Anura | Rhinella martyi | IUCN, 2024 |
| Amphibia | Anura | Rhinella multiverrucosa | IUCN, 2024 |
| Amphibia | Anura | Rhinella nesiotes | IUCN, 2024 |
| Amphibia | Anura | Rhinella ocellata | IUCN, 2024 |
| Amphibia | Anura | Rhinella poeppigii | IUCN, 2024; Present study |
| Amphibia | Anura | Rhinella proboscidea | IUCN, 2024 |
| Amphibia | Anura | Rhinella quechua | IUCN, 2024 |
| Amphibia | Anura | Rhinella roqueana | IUCN, 2024 |
| Amphibia | Anura | Rhinella rubescens | IUCN, 2024 |
| Amphibia | Anura | Rhinella spinulosa | IUCN, 2024 |
| Amphibia | Anura | Rhinella stanlaii | IUCN, 2024 |
| Amphibia | Anura | Rhinella tacana | IUCN, 2024 |
| Amphibia | Anura | Rhinella vellardi | IUCN, 2024 |
| Amphibia | Anura | Rhinella veraguensis | IUCN, 2024 |
| Amphibia | Anura | Rhinella yanachaga | IUCN, 2024 |
| Amphibia | Anura | Rulyrana flavopunctata | IUCN, 2024 |
| Amphibia | Anura | Rulyrana mcdiarmidi | IUCN, 2024 |
| Amphibia | Anura | Rulyrana saxiscandens | IUCN, 2024 |
| Amphibia | Anura | Rulyrana spiculata | IUCN, 2024 |
| Amphibia | Anura | Scarthyla goinorum | IUCN, 2024; Present study |
| Amphibia | Anura | Scinax baumgardneri | IUCN, 2024 |
| Amphibia | Anura | Scinax blairi | IUCN, 2024 |
| Amphibia | Anura | Scinax boesemani | IUCN, 2024 |
| Amphibia | Anura | Scinax castroviejoi | IUCN, 2024 |
| Amphibia | Anura | Scinax chiquitanus | IUCN, 2024 |
| Amphibia | Anura | Scinax cruentommus | IUCN, 2024; Present study |
| Amphibia | Anura | Scinax funereus | IUCN, 2024; Present study |
| Amphibia | Anura | Scinax fuscomarginatus | IUCN, 2024 |
| Amphibia | Anura | Scinax fuscovarius | IUCN, 2024 |
| Amphibia | Anura | Scinax garbei | IUCN, 2024; Present study |
| Amphibia | Anura | Scinax ictericus | IUCN, 2024 |
| Amphibia | Anura | Scinax iquitorum | IUCN, 2024 |
| Amphibia | Anura | Scinax karenanneae | IUCN, 2024 |
| Amphibia | Anura | Scinax lindsayi | IUCN, 2024 |
| Amphibia | Anura | Scinax nasicus | IUCN, 2024 |
| Amphibia | Anura | Scinax nebulosus | IUCN, 2024 |
| Amphibia | Anura | Scinax oreites | IUCN, 2024 |
| Amphibia | Anura | Scinax parkeri | IUCN, 2024 |
| Amphibia | Anura | Scinax pedromedinae | IUCN, 2024 |
| Amphibia | Anura | Scinax proboscideus | IUCN, 2024 |
| Amphibia | Anura | Scinax rostratus | IUCN, 2024 |
| Amphibia | Anura | Scinax ruber | IUCN, 2024; Present study |
| Amphibia | Anura | Scinax squalirostris | IUCN, 2024 |
| Amphibia | Anura | Scinax trilineatus | IUCN, 2024 |
| Amphibia | Anura | Scinax x-signatus | IUCN, 2024 |
| Amphibia | Anura | Sphaenorhynchus carneus | IUCN, 2024; Present study |
| Amphibia | Anura | Sphaenorhynchus dorisae | IUCN, 2024; Present study |
| Amphibia | Anura | Sphaenorhynchus lacteus | IUCN, 2024; Present study |
| Amphibia | Anura | Stefania evansi | IUCN, 2024 |
| Amphibia | Anura | Stefania roraimae | IUCN, 2024 |
| Amphibia | Anura | Stefania scalae | IUCN, 2024 |
| Amphibia | Anura | Stefania tamacuarina | IUCN, 2024 |
| Amphibia | Anura | Stefania woodleyi | IUCN, 2024 |
| Amphibia | Anura | Strabomantis cornutus | IUCN, 2024 |
| Amphibia | Anura | Strabomantis sulcatus | IUCN, 2024; Present study |
| Amphibia | Anura | Synapturanus mirandaribeiroi | IUCN, 2024 |
| Amphibia | Anura | Synapturanus rabus | IUCN, 2024; Present study |
| Amphibia | Anura | Synapturanus salseri | IUCN, 2024 |
| Amphibia | Anura | Telmatobius atahualpai | IUCN, 2024 |
| Amphibia | Anura | Telmatobius bolivianus | IUCN, 2024 |
| Amphibia | Anura | Telmatobius brachydactylus | IUCN, 2024 |
| Amphibia | Anura | Telmatobius brevipes | IUCN, 2024 |
| Amphibia | Anura | Telmatobius brevirostris | IUCN, 2024 |
| Amphibia | Anura | Telmatobius carrillae | IUCN, 2024 |
| Amphibia | Anura | Telmatobius cirrhacelis | IUCN, 2024 |
| Amphibia | Anura | Telmatobius colanensis | IUCN, 2024 |
| Amphibia | Anura | Telmatobius edaphonastes | IUCN, 2024 |
| Amphibia | Anura | Telmatobius espadai | IUCN, 2024 |
| Amphibia | Anura | Telmatobius hintoni | IUCN, 2024 |
| Amphibia | Anura | Telmatobius hockingi | IUCN, 2024 |
| Amphibia | Anura | Telmatobius ignavus | IUCN, 2024 |
| Amphibia | Anura | Telmatobius jelskii | IUCN, 2024 |
| Amphibia | Anura | Telmatobius latirostris | IUCN, 2024 |
| Amphibia | Anura | Telmatobius macrostomus | IUCN, 2024 |
| Amphibia | Anura | Telmatobius marmoratus | IUCN, 2024 |
| Amphibia | Anura | Telmatobius mendelsoni | IUCN, 2024 |
| Amphibia | Anura | Telmatobius necopinus | IUCN, 2024 |
| Amphibia | Anura | Telmatobius niger | IUCN, 2024 |
| Amphibia | Anura | Telmatobius punctatus | IUCN, 2024 |
| Amphibia | Anura | Telmatobius sanborni | IUCN, 2024 |
| Amphibia | Anura | Telmatobius sibiricus | IUCN, 2024 |
| Amphibia | Anura | Telmatobius simonsi | IUCN, 2024 |
| Amphibia | Anura | Telmatobius timens | IUCN, 2024 |
| Amphibia | Anura | Telmatobius truebae | IUCN, 2024 |
| Amphibia | Anura | Telmatobius vellardi | IUCN, 2024 |
| Amphibia | Anura | Telmatobius verrucosus | IUCN, 2024 |
| Amphibia | Anura | Telmatobius yuracare | IUCN, 2024 |
| Amphibia | Anura | Tepuihyla exophthalma | IUCN, 2024 |
| Amphibia | Anura | Tepuihyla rodriguezi | IUCN, 2024 |
| Amphibia | Anura | Tepuihyla shushupe | IUCN, 2024 |
| Amphibia | Anura | Tepuihyla tuberculosa | IUCN, 2024 |
| Amphibia | Anura | Tepuihyla warreni | IUCN, 2024 |
| Amphibia | Anura | Teratohyla adenocheira | IUCN, 2024 |
| Amphibia | Anura | Teratohyla amelie | IUCN, 2024 |
| Amphibia | Anura | Teratohyla midas | IUCN, 2024; Present study |
| Amphibia | Anura | Trachycephalus coriaceus | IUCN, 2024 |
| Amphibia | Anura | Trachycephalus hadroceps | IUCN, 2024 |
| Amphibia | Anura | Trachycephalus resinifictrix | IUCN, 2024; Present study |
| Amphibia | Anura | Trachycephalus typhonius | IUCN, 2024; Present study |
| Amphibia | Anura | Truebella skoptes | IUCN, 2024 |
| Amphibia | Anura | Truebella tothastes | IUCN, 2024 |
| Amphibia | Anura | Vitreorana gorzulae | IUCN, 2024 |
| Amphibia | Anura | Vitreorana helenae | IUCN, 2024 |
| Amphibia | Anura | Vitreorana ritae | IUCN, 2024; Present study |
| Amphibia | Anura | Yunganastes ashkapara | IUCN, 2024 |
| Amphibia | Anura | Yunganastes bisignatus | IUCN, 2024 |
| Amphibia | Anura | Yunganastes fraudator | IUCN, 2024 |
| Amphibia | Anura | Yunganastes mercedesae | IUCN, 2024 |
| Amphibia | Anura | Yunganastes pluvicanorus | IUCN, 2024 |
| Amphibia | Caudata | Bolitoglossa altamazonica | IUCN, 2024; Present study |
| Amphibia | Caudata | Bolitoglossa digitigrada | IUCN, 2024 |
| Amphibia | Caudata | Bolitoglossa equatoriana | IUCN, 2024 |
| Amphibia | Caudata | Bolitoglossa palmata | IUCN, 2024 |
| Amphibia | Caudata | Bolitoglossa peruviana | IUCN, 2024; Present study |
| Amphibia | Gymnophiona | Brasilotyphlus braziliensis | IUCN, 2024 |
| Amphibia | Gymnophiona | Caecilia abitaguae | IUCN, 2024 |
| Amphibia | Gymnophiona | Caecilia attenuata | IUCN, 2024 |
| Amphibia | Gymnophiona | Caecilia bokermanni | IUCN, 2024 |
| Amphibia | Gymnophiona | Caecilia crassisquama | IUCN, 2024 |
| Amphibia | Gymnophiona | Caecilia disossea | IUCN, 2024 |
| Amphibia | Gymnophiona | Caecilia dunni | IUCN, 2024 |
| Amphibia | Gymnophiona | Caecilia gracilis | IUCN, 2024 |
| Amphibia | Gymnophiona | Caecilia inca | IUCN, 2024 |
| Amphibia | Gymnophiona | Caecilia marcusi | IUCN, 2024 |
| Amphibia | Gymnophiona | Caecilia orientalis | IUCN, 2024 |
| Amphibia | Gymnophiona | Caecilia pachynema | IUCN, 2024 |
| Amphibia | Gymnophiona | Caecilia subdermalis | IUCN, 2024 |
| Amphibia | Gymnophiona | Caecilia tentaculata | IUCN, 2024 |
| Amphibia | Gymnophiona | Chthonerpeton onorei | IUCN, 2024 |
| Amphibia | Gymnophiona | Epicrionops bicolor | IUCN, 2024 |
| Amphibia | Gymnophiona | Epicrionops peruvianus | IUCN, 2024 |
| Amphibia | Gymnophiona | Epicrionops petersi | IUCN, 2024 |
| Amphibia | Gymnophiona | Microcaecilia albiceps | IUCN, 2024; Present study |
| Amphibia | Gymnophiona | Nectocaecilia petersii | IUCN, 2024 |
| Amphibia | Gymnophiona | Oscaecilia bassleri | IUCN, 2024 |
| Amphibia | Gymnophiona | Oscaecilia koepckeorum | IUCN, 2024 |
| Amphibia | Gymnophiona | Potamotyphlus kaupii | IUCN, 2024 |
| Amphibia | Gymnophiona | Rhinatrema nigrum | IUCN, 2024 |
| Amphibia | Gymnophiona | Siphonops annulatus | IUCN, 2024; Present study |
| Amphibia | Gymnophiona | Siphonops paulensis | IUCN, 2024 |
| Amphibia | Gymnophiona | Typhlonectes compressicauda | IUCN, 2024 |
| Reptilia | Crocodilians | Caiman crocodilus | IUCN, 2024; Present study |
| Reptilia | Crocodilians | Caiman yacare | IUCN, 2024 |
| Reptilia | Crocodilians | Crocodylus acutus | IUCN, 2024 |
| Reptilia | Crocodilians | Crocodylus intermedius | IUCN, 2024 |
| Reptilia | Crocodilians | Melanosuchus niger | IUCN, 2024; Present study |
| Reptilia | Crocodilians | Paleosuchus palpebrosus | IUCN, 2024 |
| Reptilia | Crocodilians | Paleosuchus trigonatus | IUCN, 2024; Present study |
| Reptilia | Lizard | Alopoglossus angulatus | Roll et al., 2017; Present study |
| Reptilia | Lizard | Alopoglossus atriventris | Roll et al., 2017; Present study |
| Reptilia | Lizard | Alopoglossus buckleyi | Roll et al., 2017; Present study |
| Reptilia | Lizard | Alopoglossus copii | Roll et al., 2017 |
| Reptilia | Lizard | Alopoglossus festae | Roll et al., 2017 |
| Reptilia | Lizard | Alopoglossus meloi | Roll et al., 2017 |
| Reptilia | Lizard | Amapasaurus tetradactylus | Roll et al., 2017 |
| Reptilia | Lizard | Ameiva aggerecusans | Roll et al., 2017 |
| Reptilia | Lizard | Ameiva ameiva | Roll et al., 2017; Present study |
| Reptilia | Lizard | Ameiva bifrontata | Roll et al., 2017 |
| Reptilia | Lizard | Ameiva concolor | Roll et al., 2017 |
| Reptilia | Lizard | Ameiva nodam | Roll et al., 2017 |
| Reptilia | Lizard | Ameiva parecis | Roll et al., 2017 |
| Reptilia | Lizard | Ameiva reticulata | Roll et al., 2017 |
| Reptilia | Lizard | Ameivula ocellifera | Roll et al., 2017 |
| Reptilia | Lizard | Amphisbaena alba | Roll et al., 2017; Present study |
| Reptilia | Lizard | Amphisbaena angustifrons | Roll et al., 2017 |
| Reptilia | Lizard | Amphisbaena bilabialata | Roll et al., 2017 |
| Reptilia | Lizard | Amphisbaena bolivica | Roll et al., 2017 |
| Reptilia | Lizard | Amphisbaena borelli | Roll et al., 2017 |
| Reptilia | Lizard | Amphisbaena brasiliana | Roll et al., 2017 |
| Reptilia | Lizard | Amphisbaena caiari | Roll et al., 2017 |
| Reptilia | Lizard | Amphisbaena camura | Roll et al., 2017 |
| Reptilia | Lizard | Amphisbaena cegei | Roll et al., 2017 |
| Reptilia | Lizard | Amphisbaena crisae | Roll et al., 2017 |
| Reptilia | Lizard | Amphisbaena cuiabana | Roll et al., 2017 |
| Reptilia | Lizard | Amphisbaena cunhai | Roll et al., 2017 |
| Reptilia | Lizard | Amphisbaena darwinii | Roll et al., 2017 |
| Reptilia | Lizard | Amphisbaena fuliginosa | Roll et al., 2017; Present study |
| Reptilia | Lizard | Amphisbaena hoogmoedi | Roll et al., 2017 |
| Reptilia | Lizard | Amphisbaena leeseri | Roll et al., 2017 |
| Reptilia | Lizard | Amphisbaena mitchelli | Roll et al., 2017 |
| Reptilia | Lizard | Amphisbaena neglecta | Roll et al., 2017 |
| Reptilia | Lizard | Amphisbaena occidentalis | Roll et al., 2017 |
| Reptilia | Lizard | Amphisbaena pericensis | Roll et al., 2017 |
| Reptilia | Lizard | Amphisbaena polygrammica | Roll et al., 2017 |
| Reptilia | Lizard | Amphisbaena silvestrii | Roll et al., 2017 |
| Reptilia | Lizard | Amphisbaena slateri | Roll et al., 2017 |
| Reptilia | Lizard | Amphisbaena slevini | Roll et al., 2017 |
| Reptilia | Lizard | Amphisbaena steindachneri | Roll et al., 2017 |
| Reptilia | Lizard | Amphisbaena tragorrhectes | Roll et al., 2017 |
| Reptilia | Lizard | Amphisbaena vanzolinii | Roll et al., 2017 |
| Reptilia | Lizard | Amphisbaena vermicularis | Roll et al., 2017 |
| Reptilia | Lizard | Anadia escalerae | Roll et al., 2017 |
| Reptilia | Lizard | Anadia petersi | Roll et al., 2017 |
| Reptilia | Lizard | Anadia rhombifera | Roll et al., 2017 |
| Reptilia | Lizard | Andinosaura hyposticta | Roll et al., 2017 |
| Reptilia | Lizard | Andinosaura kiziriani | Roll et al., 2017 |
| Reptilia | Lizard | Andinosaura petrorum | Roll et al., 2017 |
| Reptilia | Lizard | Andinosaura vespertina | Roll et al., 2017 |
| Reptilia | Lizard | Anolis auratus | Roll et al., 2017 |
| Reptilia | Lizard | Anolis boettgeri | Roll et al., 2017 |
| Reptilia | Lizard | Anolis bombiceps | Roll et al., 2017; Present study |
| Reptilia | Lizard | Anolis brasiliensis | Roll et al., 2017 |
| Reptilia | Lizard | Anolis caquetae | Roll et al., 2017 |
| Reptilia | Lizard | Anolis chrysolepis | Roll et al., 2017 |
| Reptilia | Lizard | Anolis cuscoensis | Roll et al., 2017 |
| Reptilia | Lizard | Anolis dissimilis | Roll et al., 2017; Present study |
| Reptilia | Lizard | Anolis fasciatus | Roll et al., 2017 |
| Reptilia | Lizard | Anolis fitchi | Roll et al., 2017 |
| Reptilia | Lizard | Anolis fraseri | Roll et al., 2017 |
| Reptilia | Lizard | Anolis fuscoauratus | Roll et al., 2017; Present study |
| Reptilia | Lizard | Anolis gemmosus | Roll et al., 2017 |
| Reptilia | Lizard | Anolis heterodermus | Roll et al., 2017 |
| Reptilia | Lizard | Anolis hyacinthogularis | Roll et al., 2017 |
| Reptilia | Lizard | Anolis laevis | Roll et al., 2017 |
| Reptilia | Lizard | Anolis lososi | Roll et al., 2017 |
| Reptilia | Lizard | Anolis meridionalis | Roll et al., 2017 |
| Reptilia | Lizard | Anolis neblininus | Roll et al., 2017 |
| Reptilia | Lizard | Anolis orcesi | Roll et al., 2017 |
| Reptilia | Lizard | Anolis ortonii | Roll et al., 2017; Present study |
| Reptilia | Lizard | Anolis peruensis | Roll et al., 2017 |
| Reptilia | Lizard | Anolis phyllorhinus | Roll et al., 2017 |
| Reptilia | Lizard | Anolis planiceps | Roll et al., 2017 |
| Reptilia | Lizard | Anolis podocarpus | Roll et al., 2017 |
| Reptilia | Lizard | Anolis punctatus | Roll et al., 2017; Present study |
| Reptilia | Lizard | Anolis scypheus | Roll et al., 2017; Present study |
| Reptilia | Lizard | Anolis soinii | Roll et al., 2017 |
| Reptilia | Lizard | Anolis tandai | Roll et al., 2017; Present study |
| Reptilia | Lizard | Anolis trachyderma | Roll et al., 2017; Present study |
| Reptilia | Lizard | Anolis transversalis | Roll et al., 2017; Present study |
| Reptilia | Lizard | Anolis vanzolinii | Roll et al., 2017 |
| Reptilia | Lizard | Anolis vaupesianus | Roll et al., 2017 |
| Reptilia | Lizard | Anolis williamsmittermeierorum | Roll et al., 2017 |
| Reptilia | Lizard | Arthrosaura kockii | Roll et al., 2017 |
| Reptilia | Lizard | Arthrosaura reticulata | Roll et al., 2017; Present study |
| Reptilia | Lizard | Arthrosaura synaptolepis | Roll et al., 2017 |
| Reptilia | Lizard | Arthrosaura versteegii | Roll et al., 2017 |
| Reptilia | Lizard | Aspronema cochabambae | Roll et al., 2017 |
| Reptilia | Lizard | Aspronema dorsivittatum | Roll et al., 2017 |
| Reptilia | Lizard | Bachia barbouri | Roll et al., 2017 |
| Reptilia | Lizard | Bachia bresslaui | Roll et al., 2017 |
| Reptilia | Lizard | Bachia didactyla | Roll et al., 2017 |
| Reptilia | Lizard | Bachia dorbignyi | Roll et al., 2017 |
| Reptilia | Lizard | Bachia flavescens | Roll et al., 2017 |
| Reptilia | Lizard | Bachia guianensis | Roll et al., 2017 |
| Reptilia | Lizard | Bachia heteropa | Roll et al., 2017 |
| Reptilia | Lizard | Bachia huallagana | Roll et al., 2017 |
| Reptilia | Lizard | Bachia intermedia | Roll et al., 2017 |
| Reptilia | Lizard | Bachia panoplia | Roll et al., 2017 |
| Reptilia | Lizard | Bachia peruana | Roll et al., 2017 |
| Reptilia | Lizard | Bachia pyburni | Roll et al., 2017 |
| Reptilia | Lizard | Bachia remota | Roll et al., 2017 |
| Reptilia | Lizard | Bachia scaea | Roll et al., 2017 |
| Reptilia | Lizard | Bachia scolecoides | Roll et al., 2017 |
| Reptilia | Lizard | Bachia trisanale | Roll et al., 2017 |
| Reptilia | Lizard | Basiliscus galeritus | Roll et al., 2017 |
| Reptilia | Lizard | Callopistes flavipunctatus | Roll et al., 2017 |
| Reptilia | Lizard | Cercosaura anordosquama | Roll et al., 2017 |
| Reptilia | Lizard | Cercosaura argulus | Roll et al., 2017; Present study |
| Reptilia | Lizard | Cercosaura bassleri | Roll et al., 2017 |
| Reptilia | Lizard | Cercosaura doanae | Roll et al., 2017 |
| Reptilia | Lizard | Cercosaura eigenmanni | Roll et al., 2017 |
| Reptilia | Lizard | Cercosaura manicata | Roll et al., 2017 |
| Reptilia | Lizard | Cercosaura ocellata | Roll et al., 2017; Present study |
| Reptilia | Lizard | Cercosaura olivacea | Roll et al., 2017 |
| Reptilia | Lizard | Cercosaura oshaughnessyi | Roll et al., 2017; Present study |
| Reptilia | Lizard | Cercosaura parkeri | Roll et al., 2017 |
| Reptilia | Lizard | Cercosaura schreibersii | Roll et al., 2017 |
| Reptilia | Lizard | Chatogekko amazonicus | Roll et al., 2017; Present study |
| Reptilia | Lizard | Cnemidophorus cryptus | Roll et al., 2017 |
| Reptilia | Lizard | Cnemidophorus gramivagus | Roll et al., 2017 |
| Reptilia | Lizard | Cnemidophorus lemniscatus | Roll et al., 2017 |
| Reptilia | Lizard | Coleodactylus brachystoma | Roll et al., 2017 |
| Reptilia | Lizard | Coleodactylus septentrionalis | Roll et al., 2017 |
| Reptilia | Lizard | Colobosaura modesta | Roll et al., 2017 |
| Reptilia | Lizard | Contomastix lacertoides | Roll et al., 2017 |
| Reptilia | Lizard | Contomastix vittata | Roll et al., 2017 |
| Reptilia | Lizard | Copeoglossum nigropunctatum | Roll et al., 2017; Present study |
| Reptilia | Lizard | Crocodilurus amazonicus | Roll et al., 2017; Present study |
| Reptilia | Lizard | Dendrosauridion yanesha | Roll et al., 2017 |
| Reptilia | Lizard | Dicrodon guttulatum | Roll et al., 2017 |
| Reptilia | Lizard | Dicrodon holmbergi | Roll et al., 2017 |
| Reptilia | Lizard | Diploglossus fasciatus | Roll et al., 2017 |
| Reptilia | Lizard | Diploglossus monotropis | Roll et al., 2017 |
| Reptilia | Lizard | Dracaena guianensis | Roll et al., 2017; Present study |
| Reptilia | Lizard | Dracaena paraguayensis | Roll et al., 2017 |
| Reptilia | Lizard | Enyalioides anisolepis | Roll et al., 2017 |
| Reptilia | Lizard | Enyalioides azulae | Roll et al., 2017 |
| Reptilia | Lizard | Enyalioides binzayedi | Roll et al., 2017 |
| Reptilia | Lizard | Enyalioides cofanorum | Roll et al., 2017 |
| Reptilia | Lizard | Enyalioides laticeps | Roll et al., 2017; Present study |
| Reptilia | Lizard | Enyalioides microlepis | Roll et al., 2017 |
| Reptilia | Lizard | Enyalioides palpebralis | Roll et al., 2017; Present study |
| Reptilia | Lizard | Enyalioides praestabilis | Roll et al., 2017 |
| Reptilia | Lizard | Enyalioides rubrigularis | Roll et al., 2017 |
| Reptilia | Lizard | Enyalioides rudolfarndti | Roll et al., 2017 |
| Reptilia | Lizard | Enyalioides sophiarothschildae | Roll et al., 2017 |
| Reptilia | Lizard | Enyalius leechii | Roll et al., 2017 |
| Reptilia | Lizard | Euspondylus caideni | Roll et al., 2017 |
| Reptilia | Lizard | Euspondylus excelsum | Roll et al., 2017 |
| Reptilia | Lizard | Euspondylus guentheri | Roll et al., 2017 |
| Reptilia | Lizard | Euspondylus josyi | Roll et al., 2017 |
| Reptilia | Lizard | Euspondylus maculatus | Roll et al., 2017 |
| Reptilia | Lizard | Euspondylus nellycarrillae | Roll et al., 2017 |
| Reptilia | Lizard | Euspondylus paxcorpus | Roll et al., 2017 |
| Reptilia | Lizard | Euspondylus simonsii | Roll et al., 2017 |
| Reptilia | Lizard | Exila nigropalmata | Roll et al., 2017 |
| Reptilia | Lizard | Gelanesaurus cochranae | Roll et al., 2017 |
| Reptilia | Lizard | Gelanesaurus flavogularis | Roll et al., 2017 |
| Reptilia | Lizard | Gonatodes albogularis | Roll et al., 2017 |
| Reptilia | Lizard | Gonatodes alexandermendesi | Roll et al., 2017 |
| Reptilia | Lizard | Gonatodes annularis | Roll et al., 2017 |
| Reptilia | Lizard | Gonatodes atricucullaris | Roll et al., 2017 |
| Reptilia | Lizard | Gonatodes caudiscutatus | Roll et al., 2017 |
| Reptilia | Lizard | Gonatodes concinnatus | Roll et al., 2017; Present study |
| Reptilia | Lizard | Gonatodes eladioi | Roll et al., 2017 |
| Reptilia | Lizard | Gonatodes hasemani | Roll et al., 2017 |
| Reptilia | Lizard | Gonatodes humeralis | Roll et al., 2017; Present study |
| Reptilia | Lizard | Gonatodes nascimentoi | Roll et al., 2017 |
| Reptilia | Lizard | Gonatodes riveroi | Roll et al., 2017 |
| Reptilia | Lizard | Gonatodes tapajonicus | Roll et al., 2017 |
| Reptilia | Lizard | Gymnodactylus amarali | Roll et al., 2017 |
| Reptilia | Lizard | Gymnophthalmus cryptus | Roll et al., 2017 |
| Reptilia | Lizard | Gymnophthalmus leucomystax | Roll et al., 2017 |
| Reptilia | Lizard | Gymnophthalmus lineatus | Roll et al., 2017 |
| Reptilia | Lizard | Gymnophthalmus underwoodi | Roll et al., 2017 |
| Reptilia | Lizard | Gymnophthalmus vanzoi | Roll et al., 2017 |
| Reptilia | Lizard | Hemidactylus palaichthus | Roll et al., 2017 |
| Reptilia | Lizard | Holcosus septemlineatus | Roll et al., 2017 |
| Reptilia | Lizard | Homonota horrida | Roll et al., 2017 |
| Reptilia | Lizard | Hoplocercus spinosus | Roll et al., 2017 |
| Reptilia | Lizard | Iguana iguana | Roll et al., 2017; Present study |
| Reptilia | Lizard | Iphisa elegans | Roll et al., 2017; Present study |
| Reptilia | Lizard | Kentropyx altamazonica | Roll et al., 2017; Present study |
| Reptilia | Lizard | Kentropyx calcarata | Roll et al., 2017 |
| Reptilia | Lizard | Kentropyx paulensis | Roll et al., 2017 |
| Reptilia | Lizard | Kentropyx pelviceps | Roll et al., 2017; Present study |
| Reptilia | Lizard | Kentropyx striata | Roll et al., 2017 |
| Reptilia | Lizard | Kentropyx vanzoi | Roll et al., 2017 |
| Reptilia | Lizard | Kentropyx viridistriga | Roll et al., 2017 |
| Reptilia | Lizard | Lepidoblepharis conolepis | Roll et al., 2017 |
| Reptilia | Lizard | Lepidoblepharis festae | Roll et al., 2017 |
| Reptilia | Lizard | Lepidoblepharis heyerorum | Roll et al., 2017 |
| Reptilia | Lizard | Lepidoblepharis hoogmoedi | Roll et al., 2017 |
| Reptilia | Lizard | Lepidoblepharis xanthostigma | Roll et al., 2017 |
| Reptilia | Lizard | Leposternon infraorbitale | Roll et al., 2017 |
| Reptilia | Lizard | Leposternon microcephalum | Roll et al., 2017 |
| Reptilia | Lizard | Leposternon polystegum | Roll et al., 2017 |
| Reptilia | Lizard | Liolaemus annectens | Roll et al., 2017 |
| Reptilia | Lizard | Liolaemus aparicioi | Roll et al., 2017 |
| Reptilia | Lizard | Liolaemus chavin | Roll et al., 2017 |
| Reptilia | Lizard | Liolaemus cranwelli | Roll et al., 2017 |
| Reptilia | Lizard | Liolaemus fittkaui | Roll et al., 2017 |
| Reptilia | Lizard | Liolaemus forsteri | Roll et al., 2017 |
| Reptilia | Lizard | Liolaemus incaicus | Roll et al., 2017 |
| Reptilia | Lizard | Liolaemus melanogaster | Roll et al., 2017 |
| Reptilia | Lizard | Liolaemus ortizii | Roll et al., 2017 |
| Reptilia | Lizard | Liolaemus pachacutec | Roll et al., 2017 |
| Reptilia | Lizard | Liolaemus polystictus | Roll et al., 2017 |
| Reptilia | Lizard | Liolaemus puna | Roll et al., 2017 |
| Reptilia | Lizard | Liolaemus robustus | Roll et al., 2017 |
| Reptilia | Lizard | Liolaemus signifer | Roll et al., 2017 |
| Reptilia | Lizard | Liolaemus thomasi | Roll et al., 2017 |
| Reptilia | Lizard | Liolaemus variegatus | Roll et al., 2017 |
| Reptilia | Lizard | Liolaemus walkeri | Roll et al., 2017 |
| Reptilia | Lizard | Liolaemus wari | Roll et al., 2017 |
| Reptilia | Lizard | Loxopholis ferreirai | Roll et al., 2017 |
| Reptilia | Lizard | Loxopholis guianense | Roll et al., 2017 |
| Reptilia | Lizard | Loxopholis hexalepis | Roll et al., 2017 |
| Reptilia | Lizard | Loxopholis osvaldoi | Roll et al., 2017 |
| Reptilia | Lizard | Loxopholis parietalis | Roll et al., 2017; Present study |
| Reptilia | Lizard | Loxopholis percarinatum | Roll et al., 2017 |
| Reptilia | Lizard | Loxopholis snethlageae | Roll et al., 2017; Present study |
| Reptilia | Lizard | Lygodactylus wetzeli | Roll et al., 2017 |
| Reptilia | Lizard | Macropholidus annectens | Roll et al., 2017 |
| Reptilia | Lizard | Macropholidus ruthveni | Roll et al., 2017 |
| Reptilia | Lizard | Manciola guaporicola | Roll et al., 2017 |
| Reptilia | Lizard | Marinussaurus curupira | Roll et al., 2017 |
| Reptilia | Lizard | Mesobaena huebneri | Roll et al., 2017 |
| Reptilia | Lizard | Mesobaena rhachicephala | Roll et al., 2017 |
| Reptilia | Lizard | Micrablepharus atticolus | Roll et al., 2017 |
| Reptilia | Lizard | Micrablepharus maximiliani | Roll et al., 2017 |
| Reptilia | Lizard | Microlophus occipitalis | Roll et al., 2017 |
| Reptilia | Lizard | Microlophus stolzmanni | Roll et al., 2017 |
| Reptilia | Lizard | Morunasaurus annularis | Roll et al., 2017 |
| Reptilia | Lizard | Morunasaurus peruvianus | Roll et al., 2017 |
| Reptilia | Lizard | Neusticurus arekuna | Roll et al., 2017 |
| Reptilia | Lizard | Neusticurus bicarinatus | Roll et al., 2017 |
| Reptilia | Lizard | Neusticurus medemi | Roll et al., 2017 |
| Reptilia | Lizard | Neusticurus racenisi | Roll et al., 2017 |
| Reptilia | Lizard | Neusticurus rudis | Roll et al., 2017 |
| Reptilia | Lizard | Neusticurus surinamensis | Roll et al., 2017 |
| Reptilia | Lizard | Neusticurus tatei | Roll et al., 2017 |
| Reptilia | Lizard | Notomabuya frenata | Roll et al., 2017 |
| Reptilia | Lizard | Ophiodes fragilis | Roll et al., 2017 |
| Reptilia | Lizard | Ophiodes striatus | Roll et al., 2017 |
| Reptilia | Lizard | Panopa carvalhoi | Roll et al., 2017 |
| Reptilia | Lizard | Petracola angustisoma | Roll et al., 2017 |
| Reptilia | Lizard | Petracola labioocularis | Roll et al., 2017 |
| Reptilia | Lizard | Petracola ventrimaculatus | Roll et al., 2017 |
| Reptilia | Lizard | Petracola waka | Roll et al., 2017 |
| Reptilia | Lizard | Pholidobolus affinis | Roll et al., 2017 |
| Reptilia | Lizard | Pholidobolus anomalus | Roll et al., 2017 |
| Reptilia | Lizard | Pholidobolus dicrus | Roll et al., 2017 |
| Reptilia | Lizard | Pholidobolus hillisi | Roll et al., 2017 |
| Reptilia | Lizard | Pholidobolus macbrydei | Roll et al., 2017 |
| Reptilia | Lizard | Pholidobolus montium | Roll et al., 2017 |
| Reptilia | Lizard | Pholidobolus prefrontalis | Roll et al., 2017 |
| Reptilia | Lizard | Pholidobolus ulisesi | Roll et al., 2017 |
| Reptilia | Lizard | Pholidobolus vertebralis | Roll et al., 2017 |
| Reptilia | Lizard | Phyllodactylus delsolari | Roll et al., 2017 |
| Reptilia | Lizard | Phyllodactylus interandinus | Roll et al., 2017 |
| Reptilia | Lizard | Phyllodactylus johnwrighti | Roll et al., 2017 |
| Reptilia | Lizard | Phyllodactylus magister | Roll et al., 2017 |
| Reptilia | Lizard | Phyllodactylus pachamama | Roll et al., 2017 |
| Reptilia | Lizard | Phyllodactylus reissii | Roll et al., 2017 |
| Reptilia | Lizard | Phyllodactylus thompsoni | Roll et al., 2017 |
| Reptilia | Lizard | Phyllopezus maranjonensis | Roll et al., 2017 |
| Reptilia | Lizard | Phyllopezus pollicaris | Roll et al., 2017 |
| Reptilia | Lizard | Phyllopezus przewalskii | Roll et al., 2017 |
| Reptilia | Lizard | Plica medemi | Roll et al., 2017 |
| Reptilia | Lizard | Plica plica | Roll et al., 2017; Present study |
| Reptilia | Lizard | Plica umbra | Roll et al., 2017; Present study |
| Reptilia | Lizard | Polychrus acutirostris | Roll et al., 2017 |
| Reptilia | Lizard | Polychrus femoralis | Roll et al., 2017 |
| Reptilia | Lizard | Polychrus jacquelinae | Roll et al., 2017 |
| Reptilia | Lizard | Polychrus liogaster | Roll et al., 2017 |
| Reptilia | Lizard | Polychrus marmoratus | Roll et al., 2017; Present study |
| Reptilia | Lizard | Polychrus peruvianus | Roll et al., 2017 |
| Reptilia | Lizard | Potamites ecpleopus | Roll et al., 2017; Present study |
| Reptilia | Lizard | Potamites erythrocularis | Roll et al., 2017 |
| Reptilia | Lizard | Potamites juruazensis | Roll et al., 2017 |
| Reptilia | Lizard | Potamites montanicola | Roll et al., 2017 |
| Reptilia | Lizard | Potamites ocellatus | Roll et al., 2017 |
| Reptilia | Lizard | Potamites strangulatus | Roll et al., 2017 |
| Reptilia | Lizard | Proctoporus bolivianus | Roll et al., 2017 |
| Reptilia | Lizard | Proctoporus carabaya | Roll et al., 2017 |
| Reptilia | Lizard | Proctoporus chasqui | Roll et al., 2017 |
| Reptilia | Lizard | Proctoporus guentheri | Roll et al., 2017 |
| Reptilia | Lizard | Proctoporus iridescens | Roll et al., 2017 |
| Reptilia | Lizard | Proctoporus kiziriani | Roll et al., 2017 |
| Reptilia | Lizard | Proctoporus lacertus | Roll et al., 2017 |
| Reptilia | Lizard | Proctoporus laudahnae | Roll et al., 2017 |
| Reptilia | Lizard | Proctoporus machupicchu | Roll et al., 2017 |
| Reptilia | Lizard | Proctoporus oreades | Roll et al., 2017 |
| Reptilia | Lizard | Proctoporus pachyurus | Roll et al., 2017 |
| Reptilia | Lizard | Proctoporus rahmi | Roll et al., 2017 |
| Reptilia | Lizard | Proctoporus spinalis | Roll et al., 2017 |
| Reptilia | Lizard | Proctoporus sucullucu | Roll et al., 2017 |
| Reptilia | Lizard | Proctoporus unsaacae | Roll et al., 2017 |
| Reptilia | Lizard | Proctoporus xestus | Roll et al., 2017 |
| Reptilia | Lizard | Pseudogonatodes barbouri | Roll et al., 2017 |
| Reptilia | Lizard | Pseudogonatodes gasconi | Roll et al., 2017 |
| Reptilia | Lizard | Pseudogonatodes guianensis | Roll et al., 2017; Present study |
| Reptilia | Lizard | Pseudogonatodes peruvianus | Roll et al., 2017 |
| Reptilia | Lizard | Ptychoglossus brevifrontalis | Roll et al., 2017; Present study |
| Reptilia | Lizard | Riama anatoloros | Roll et al., 2017 |
| Reptilia | Lizard | Riama balneator | Roll et al., 2017 |
| Reptilia | Lizard | Riama meleagris | Roll et al., 2017 |
| Reptilia | Lizard | Riama orcesi | Roll et al., 2017 |
| Reptilia | Lizard | Riama raneyi | Roll et al., 2017 |
| Reptilia | Lizard | Riama simotera | Roll et al., 2017 |
| Reptilia | Lizard | Riama stigmatoral | Roll et al., 2017 |
| Reptilia | Lizard | Riama striata | Roll et al., 2017 |
| Reptilia | Lizard | Riama unicolor | Roll et al., 2017 |
| Reptilia | Lizard | Riolama leucosticta | Roll et al., 2017 |
| Reptilia | Lizard | Rondonops biscutatus | Roll et al., 2017 |
| Reptilia | Lizard | Rondonops xanthomystax | Roll et al., 2017 |
| Reptilia | Lizard | Salvator merianae | Roll et al., 2017 |
| Reptilia | Lizard | Salvator rufescens | Roll et al., 2017 |
| Reptilia | Lizard | Selvasaura brava | Roll et al., 2017 |
| Reptilia | Lizard | Stenocercus aculeatus | Roll et al., 2017 |
| Reptilia | Lizard | Stenocercus albolineatus | Roll et al., 2017 |
| Reptilia | Lizard | Stenocercus angel | Roll et al., 2017 |
| Reptilia | Lizard | Stenocercus angulifer | Roll et al., 2017 |
| Reptilia | Lizard | Stenocercus apurimacus | Roll et al., 2017 |
| Reptilia | Lizard | Stenocercus arndti | Roll et al., 2017 |
| Reptilia | Lizard | Stenocercus boettgeri | Roll et al., 2017 |
| Reptilia | Lizard | Stenocercus cadlei | Roll et al., 2017 |
| Reptilia | Lizard | Stenocercus caducus | Roll et al., 2017 |
| Reptilia | Lizard | Stenocercus chinchaoensis | Roll et al., 2017 |
| Reptilia | Lizard | Stenocercus chlorostictus | Roll et al., 2017 |
| Reptilia | Lizard | Stenocercus chrysopygus | Roll et al., 2017 |
| Reptilia | Lizard | Stenocercus crassicaudatus | Roll et al., 2017 |
| Reptilia | Lizard | Stenocercus cupreus | Roll et al., 2017 |
| Reptilia | Lizard | Stenocercus empetrus | Roll et al., 2017 |
| Reptilia | Lizard | Stenocercus festae | Roll et al., 2017 |
| Reptilia | Lizard | Stenocercus fimbriatus | Roll et al., 2017; Present study |
| Reptilia | Lizard | Stenocercus formosus | Roll et al., 2017 |
| Reptilia | Lizard | Stenocercus frittsi | Roll et al., 2017 |
| Reptilia | Lizard | Stenocercus guentheri | Roll et al., 2017 |
| Reptilia | Lizard | Stenocercus huancabambae | Roll et al., 2017 |
| Reptilia | Lizard | Stenocercus humeralis | Roll et al., 2017 |
| Reptilia | Lizard | Stenocercus imitator | Roll et al., 2017 |
| Reptilia | Lizard | Stenocercus iridescens | Roll et al., 2017 |
| Reptilia | Lizard | Stenocercus ivitus | Roll et al., 2017 |
| Reptilia | Lizard | Stenocercus latebrosus | Roll et al., 2017 |
| Reptilia | Lizard | Stenocercus marmoratus | Roll et al., 2017 |
| Reptilia | Lizard | Stenocercus melanopygus | Roll et al., 2017 |
| Reptilia | Lizard | Stenocercus nigromaculatus | Roll et al., 2017 |
| Reptilia | Lizard | Stenocercus nubicola | Roll et al., 2017 |
| Reptilia | Lizard | Stenocercus ochoai | Roll et al., 2017 |
| Reptilia | Lizard | Stenocercus omari | Roll et al., 2017 |
| Reptilia | Lizard | Stenocercus orientalis | Roll et al., 2017 |
| Reptilia | Lizard | Stenocercus ornatus | Roll et al., 2017 |
| Reptilia | Lizard | Stenocercus percultus | Roll et al., 2017 |
| Reptilia | Lizard | Stenocercus praeornatus | Roll et al., 2017 |
| Reptilia | Lizard | Stenocercus prionotus | Roll et al., 2017 |
| Reptilia | Lizard | Stenocercus puyango | Roll et al., 2017 |
| Reptilia | Lizard | Stenocercus rhodomelas | Roll et al., 2017 |
| Reptilia | Lizard | Stenocercus roseiventris | Roll et al., 2017 |
| Reptilia | Lizard | Stenocercus scapularis | Roll et al., 2017 |
| Reptilia | Lizard | Stenocercus simonsii | Roll et al., 2017 |
| Reptilia | Lizard | Stenocercus sinesaccus | Roll et al., 2017 |
| Reptilia | Lizard | Stenocercus stigmosus | Roll et al., 2017 |
| Reptilia | Lizard | Stenocercus torquatus | Roll et al., 2017 |
| Reptilia | Lizard | Stenocercus variabilis | Roll et al., 2017 |
| Reptilia | Lizard | Teius teyou | Roll et al., 2017 |
| Reptilia | Lizard | Thecadactylus rapicauda | Roll et al., 2017 |
| Reptilia | Lizard | Thecadactylus solimoensis | Roll et al., 2017; Present study |
| Reptilia | Lizard | Tretioscincus agilis | Roll et al., 2017 |
| Reptilia | Lizard | Tretioscincus oriximinensis | Roll et al., 2017 |
| Reptilia | Lizard | Tropidurus arenarius | Roll et al., 2017 |
| Reptilia | Lizard | Tropidurus azurduyae | Roll et al., 2017 |
| Reptilia | Lizard | Tropidurus callathelys | Roll et al., 2017 |
| Reptilia | Lizard | Tropidurus catalanensis | Roll et al., 2017 |
| Reptilia | Lizard | Tropidurus chromatops | Roll et al., 2017 |
| Reptilia | Lizard | Tropidurus etheridgei | Roll et al., 2017 |
| Reptilia | Lizard | Tropidurus guarani | Roll et al., 2017 |
| Reptilia | Lizard | Tropidurus hispidus | Roll et al., 2017 |
| Reptilia | Lizard | Tropidurus insulanus | Roll et al., 2017 |
| Reptilia | Lizard | Tropidurus itambere | Roll et al., 2017 |
| Reptilia | Lizard | Tropidurus lagunablanca | Roll et al., 2017 |
| Reptilia | Lizard | Tropidurus melanopleurus | Roll et al., 2017 |
| Reptilia | Lizard | Tropidurus oreadicus | Roll et al., 2017 |
| Reptilia | Lizard | Tropidurus spinulosus | Roll et al., 2017 |
| Reptilia | Lizard | Tropidurus torquatus | Roll et al., 2017 |
| Reptilia | Lizard | Tropidurus xanthochilus | Roll et al., 2017 |
| Reptilia | Lizard | Tupinambis cryptus | Roll et al., 2017 |
| Reptilia | Lizard | Tupinambis cuzcoensis | Roll et al., 2017 |
| Reptilia | Lizard | Tupinambis longilineus | Roll et al., 2017 |
| Reptilia | Lizard | Tupinambis matipu | Roll et al., 2017 |
| Reptilia | Lizard | Tupinambis quadrilineatus | Roll et al., 2017 |
| Reptilia | Lizard | Tupinambis teguixin | Roll et al., 2017; Present study |
| Reptilia | Lizard | Uracentron azureum | Roll et al., 2017 |
| Reptilia | Lizard | Uracentron flaviceps | Roll et al., 2017; Present study |
| Reptilia | Lizard | Uranoscodon superciliosus | Roll et al., 2017 |
| Reptilia | Lizard | Urostrophus gallardoi | Roll et al., 2017 |
| Reptilia | Lizard | Vanzosaura rubricauda | Roll et al., 2017 |
| Reptilia | Lizard | Varzea altamazonica | Roll et al., 2017; Present study |
| Reptilia | Lizard | Varzea bistriata | Roll et al., 2017 |
| Reptilia | Lizard | Yanomamia guianensis | Roll et al., 2017 |
| Reptilia | Snake | Amerotyphlops brongersmianus | Roll et al., 2017 |
| Reptilia | Snake | Amerotyphlops minuisquamus | Roll et al., 2017 |
| Reptilia | Snake | Amerotyphlops reticulatus | Roll et al., 2017 |
| Reptilia | Snake | Anilius scytale | Roll et al., 2017; Present study |
| Reptilia | Snake | Anomalepis aspinosus | Roll et al., 2017 |
| Reptilia | Snake | Anomalepis mexicanus | Roll et al., 2017 |
| Reptilia | Snake | Apostolepis ambiniger | Roll et al., 2017 |
| Reptilia | Snake | Apostolepis ammodites | Roll et al., 2017 |
| Reptilia | Snake | Apostolepis assimilis | Roll et al., 2017 |
| Reptilia | Snake | Apostolepis christineae | Roll et al., 2017 |
| Reptilia | Snake | Apostolepis flavotorquata | Roll et al., 2017 |
| Reptilia | Snake | Apostolepis multicincta | Roll et al., 2017 |
| Reptilia | Snake | Apostolepis niceforoi | Roll et al., 2017 |
| Reptilia | Snake | Apostolepis nigrolineata | Roll et al., 2017 |
| Reptilia | Snake | Apostolepis nigroterminata | Roll et al., 2017 |
| Reptilia | Snake | Apostolepis phillipsae | Roll et al., 2017 |
| Reptilia | Snake | Apostolepis pymi | Roll et al., 2017 |
| Reptilia | Snake | Apostolepis striata | Roll et al., 2017 |
| Reptilia | Snake | Apostolepis tenuis | Roll et al., 2017 |
| Reptilia | Snake | Apostolepis underwoodi | Roll et al., 2017 |
| Reptilia | Snake | Apostolepis vittata | Roll et al., 2017 |
| Reptilia | Snake | Atractus albuquerquei | Roll et al., 2017 |
| Reptilia | Snake | Atractus alphonsehogei | Roll et al., 2017 |
| Reptilia | Snake | Atractus altagratiae | Roll et al., 2017 |
| Reptilia | Snake | Atractus apophis | Roll et al., 2017 |
| Reptilia | Snake | Atractus arangoi | Roll et al., 2017 |
| Reptilia | Snake | Atractus atlas | Roll et al., 2017 |
| Reptilia | Snake | Atractus avernus | Roll et al., 2017 |
| Reptilia | Snake | Atractus badius | Roll et al., 2017 |
| Reptilia | Snake | Atractus balzani | Roll et al., 2017 |
| Reptilia | Snake | Atractus bocki | Roll et al., 2017 |
| Reptilia | Snake | Atractus bocourti | Roll et al., 2017 |
| Reptilia | Snake | Atractus boettgeri | Roll et al., 2017 |
| Reptilia | Snake | Atractus boimirim | Roll et al., 2017 |
| Reptilia | Snake | Atractus carrioni | Roll et al., 2017 |
| Reptilia | Snake | Atractus caxiuana | Roll et al., 2017 |
| Reptilia | Snake | Atractus charitoae | Roll et al., 2017 |
| Reptilia | Snake | Atractus chthonius | Roll et al., 2017 |
| Reptilia | Snake | Atractus collaris | Roll et al., 2017 |
| Reptilia | Snake | Atractus dapsilis | Roll et al., 2017 |
| Reptilia | Snake | Atractus duboisi | Roll et al., 2017 |
| Reptilia | Snake | Atractus duidensis | Roll et al., 2017 |
| Reptilia | Snake | Atractus dunni | Roll et al., 2017 |
| Reptilia | Snake | Atractus ecuadorensis | Roll et al., 2017 |
| Reptilia | Snake | Atractus elaps | Roll et al., 2017 |
| Reptilia | Snake | Atractus emmeli | Roll et al., 2017 |
| Reptilia | Snake | Atractus favae | Roll et al., 2017 |
| Reptilia | Snake | Atractus flammigerus | Roll et al., 2017 |
| Reptilia | Snake | Atractus franciscopaivai | Roll et al., 2017 |
| Reptilia | Snake | Atractus gaigeae | Roll et al., 2017 |
| Reptilia | Snake | Atractus gigas | Roll et al., 2017 |
| Reptilia | Snake | Atractus heliobelluomini | Roll et al., 2017 |
| Reptilia | Snake | Atractus insipidus | Roll et al., 2017 |
| Reptilia | Snake | Atractus latifrons | Roll et al., 2017 |
| Reptilia | Snake | Atractus lehmanni | Roll et al., 2017 |
| Reptilia | Snake | Atractus major | Roll et al., 2017; Present study |
| Reptilia | Snake | Atractus modestus | Roll et al., 2017 |
| Reptilia | Snake | Atractus natans | Roll et al., 2017 |
| Reptilia | Snake | Atractus nigricaudus | Roll et al., 2017 |
| Reptilia | Snake | Atractus occipitoalbus | Roll et al., 2017 |
| Reptilia | Snake | Atractus orcesi | Roll et al., 2017 |
| Reptilia | Snake | Atractus pantostictus | Roll et al., 2017 |
| Reptilia | Snake | Atractus paravertebralis | Roll et al., 2017 |
| Reptilia | Snake | Atractus pauciscutatus | Roll et al., 2017 |
| Reptilia | Snake | Atractus peruvianus | Roll et al., 2017 |
| Reptilia | Snake | Atractus poeppigi | Roll et al., 2017 |
| Reptilia | Snake | Atractus resplendens | Roll et al., 2017 |
| Reptilia | Snake | Atractus riveroi | Roll et al., 2017 |
| Reptilia | Snake | Atractus roulei | Roll et al., 2017 |
| Reptilia | Snake | Atractus schach | Roll et al., 2017 |
| Reptilia | Snake | Atractus snethlageae | Roll et al., 2017 |
| Reptilia | Snake | Atractus steyermarki | Roll et al., 2017 |
| Reptilia | Snake | Atractus surucucu | Roll et al., 2017 |
| Reptilia | Snake | Atractus taeniatus | Roll et al., 2017 |
| Reptilia | Snake | Atractus tamessari | Roll et al., 2017 |
| Reptilia | Snake | Atractus tartarus | Roll et al., 2017 |
| Reptilia | Snake | Atractus torquatus | Roll et al., 2017 |
| Reptilia | Snake | Atractus touzeti | Roll et al., 2017 |
| Reptilia | Snake | Atractus trefauti | Roll et al., 2017 |
| Reptilia | Snake | Atractus trilineatus | Roll et al., 2017 |
| Reptilia | Snake | Atractus vertebralis | Roll et al., 2017 |
| Reptilia | Snake | Atractus zidoki | Roll et al., 2017 |
| Reptilia | Snake | Boa constrictor | Roll et al., 2017; Present study |
| Reptilia | Snake | Boiruna maculata | Roll et al., 2017 |
| Reptilia | Snake | Bothriechis schlegelii | Roll et al., 2017 |
| Reptilia | Snake | Bothrocophias andianus | Roll et al., 2017 |
| Reptilia | Snake | Bothrocophias hyoprora | Roll et al., 2017; Present study |
| Reptilia | Snake | Bothrocophias microphthalmus | Roll et al., 2017 |
| Reptilia | Snake | Bothrops asper | Roll et al., 2017 |
| Reptilia | Snake | Bothrops atrox | Roll et al., 2017; Present study |
| Reptilia | Snake | Bothrops ayerbei | Roll et al., 2017 |
| Reptilia | Snake | Bothrops barnetti | Roll et al., 2017 |
| Reptilia | Snake | Bothrops bilineatus | Roll et al., 2017; Present study |
| Reptilia | Snake | Bothrops brazili | Roll et al., 2017; Present study |
| Reptilia | Snake | Bothrops chloromelas | Roll et al., 2017 |
| Reptilia | Snake | Bothrops itapetiningae | Roll et al., 2017 |
| Reptilia | Snake | Bothrops jonathani | Roll et al., 2017 |
| Reptilia | Snake | Bothrops lojanus | Roll et al., 2017 |
| Reptilia | Snake | Bothrops marajoensis | Roll et al., 2017 |
| Reptilia | Snake | Bothrops matogrossensis | Roll et al., 2017 |
| Reptilia | Snake | Bothrops moojeni | Roll et al., 2017 |
| Reptilia | Snake | Bothrops oligolepis | Roll et al., 2017 |
| Reptilia | Snake | Bothrops osbornei | Roll et al., 2017 |
| Reptilia | Snake | Bothrops pauloensis | Roll et al., 2017 |
| Reptilia | Snake | Bothrops pictus | Roll et al., 2017 |
| Reptilia | Snake | Bothrops pulcher | Roll et al., 2017 |
| Reptilia | Snake | Bothrops sanctaecrucis | Roll et al., 2017 |
| Reptilia | Snake | Bothrops sonene | Roll et al., 2017 |
| Reptilia | Snake | Bothrops taeniatus | Roll et al., 2017 |
| Reptilia | Snake | Chironius carinatus | Roll et al., 2017 |
| Reptilia | Snake | Chironius exoletus | Roll et al., 2017; Present study |
| Reptilia | Snake | Chironius flavolineatus | Roll et al., 2017 |
| Reptilia | Snake | Chironius fuscus | Roll et al., 2017; Present study |
| Reptilia | Snake | Chironius grandisquamis | Roll et al., 2017 |
| Reptilia | Snake | Chironius laurenti | Roll et al., 2017 |
| Reptilia | Snake | Chironius leucometapus | Roll et al., 2017 |
| Reptilia | Snake | Chironius maculoventris | Roll et al., 2017 |
| Reptilia | Snake | Chironius monticola | Roll et al., 2017 |
| Reptilia | Snake | Chironius multiventris | Roll et al., 2017; Present study |
| Reptilia | Snake | Chironius quadricarinatus | Roll et al., 2017 |
| Reptilia | Snake | Chironius scurrulus | Roll et al., 2017 |
| Reptilia | Snake | Chironius septentrionalis | Roll et al., 2017 |
| Reptilia | Snake | Clelia clelia | Roll et al., 2017; Present study |
| Reptilia | Snake | Clelia equatoriana | Roll et al., 2017 |
| Reptilia | Snake | Clelia langeri | Roll et al., 2017 |
| Reptilia | Snake | Coniophanes dromiciformis | Roll et al., 2017 |
| Reptilia | Snake | Corallus annulatus | Roll et al., 2017 |
| Reptilia | Snake | Corallus batesii | Roll et al., 2017 |
| Reptilia | Snake | Corallus caninus | Roll et al., 2017 |
| Reptilia | Snake | Corallus hortulanus | Roll et al., 2017; Present study |
| Reptilia | Snake | Crotalus durissus | Roll et al., 2017 |
| Reptilia | Snake | Dendrophidion bivittatus | Roll et al., 2017 |
| Reptilia | Snake | Dendrophidion brunneum | Roll et al., 2017 |
| Reptilia | Snake | Dendrophidion clarkii | Roll et al., 2017 |
| Reptilia | Snake | Dendrophidion dendrophis | Roll et al., 2017; Present study |
| Reptilia | Snake | Dipsas bucephala | Roll et al., 2017 |
| Reptilia | Snake | Dipsas catesbyi | Roll et al., 2017; Present study |
| Reptilia | Snake | Dipsas chaparensis | Roll et al., 2017 |
| Reptilia | Snake | Dipsas cisticeps | Roll et al., 2017 |
| Reptilia | Snake | Dipsas copei | Roll et al., 2017 |
| Reptilia | Snake | Dipsas gracilis | Roll et al., 2017 |
| Reptilia | Snake | Dipsas incerta | Roll et al., 2017 |
| Reptilia | Snake | Dipsas indica | Roll et al., 2017; Present study |
| Reptilia | Snake | Dipsas jamespetersi | Roll et al., 2017 |
| Reptilia | Snake | Dipsas klebbai | Roll et al., 2017 |
| Reptilia | Snake | Dipsas lavillai | Roll et al., 2017 |
| Reptilia | Snake | Dipsas mikanii | Roll et al., 2017 |
| Reptilia | Snake | Dipsas oligozonata | Roll et al., 2017 |
| Reptilia | Snake | Dipsas oneilli | Roll et al., 2017 |
| Reptilia | Snake | Dipsas oreas | Roll et al., 2017 |
| Reptilia | Snake | Dipsas palmeri | Roll et al., 2017 |
| Reptilia | Snake | Dipsas pavonina | Roll et al., 2017 |
| Reptilia | Snake | Dipsas peruana | Roll et al., 2017 |
| Reptilia | Snake | Dipsas schunkii | Roll et al., 2017 |
| Reptilia | Snake | Dipsas temporalis | Roll et al., 2017 |
| Reptilia | Snake | Dipsas turgidus | Roll et al., 2017 |
| Reptilia | Snake | Dipsas vagrans | Roll et al., 2017 |
| Reptilia | Snake | Dipsas vagus | Roll et al., 2017 |
| Reptilia | Snake | Dipsas variegata | Roll et al., 2017 |
| Reptilia | Snake | Dipsas vermiculata | Roll et al., 2017 |
| Reptilia | Snake | Drepanoides anomalus | Roll et al., 2017; Present study |
| Reptilia | Snake | Drymarchon corais | Roll et al., 2017; Present study |
| Reptilia | Snake | Drymarchon melanurus | Roll et al., 2017 |
| Reptilia | Snake | Drymobius rhombifer | Roll et al., 2017 |
| Reptilia | Snake | Drymoluber apurimacensis | Roll et al., 2017 |
| Reptilia | Snake | Drymoluber brazili | Roll et al., 2017 |
| Reptilia | Snake | Drymoluber dichrous | Roll et al., 2017; Present study |
| Reptilia | Snake | Epicrates alvarezi | Roll et al., 2017 |
| Reptilia | Snake | Epicrates cenchria | Roll et al., 2017; Present study |
| Reptilia | Snake | Epicrates crassus | Roll et al., 2017 |
| Reptilia | Snake | Epicrates maurus | Roll et al., 2017 |
| Reptilia | Snake | Epictia albipuncta | Roll et al., 2017 |
| Reptilia | Snake | Epictia antoniogarciai | Roll et al., 2017 |
| Reptilia | Snake | Epictia diaplocia | Roll et al., 2017 |
| Reptilia | Snake | Epictia goudotii | Roll et al., 2017 |
| Reptilia | Snake | Epictia peruviana | Roll et al., 2017 |
| Reptilia | Snake | Epictia septemlineata | Roll et al., 2017 |
| Reptilia | Snake | Epictia striatula | Roll et al., 2017 |
| Reptilia | Snake | Epictia tenella | Roll et al., 2017; Present study |
| Reptilia | Snake | Epictia tricolor | Roll et al., 2017 |
| Reptilia | Snake | Epictia undecimstriata | Roll et al., 2017 |
| Reptilia | Snake | Epictia vanwallachi | Roll et al., 2017 |
| Reptilia | Snake | Epictia vonmayi | Roll et al., 2017 |
| Reptilia | Snake | Erythrolamprus aesculapii | Roll et al., 2017 |
| Reptilia | Snake | Erythrolamprus albertguentheri | Roll et al., 2017 |
| Reptilia | Snake | Erythrolamprus almadensis | Roll et al., 2017 |
| Reptilia | Snake | Erythrolamprus andinus | Roll et al., 2017 |
| Reptilia | Snake | Erythrolamprus bizona | Roll et al., 2017 |
| Reptilia | Snake | Erythrolamprus breviceps | Roll et al., 2017; Present study |
| Reptilia | Snake | Erythrolamprus ceii | Roll et al., 2017 |
| Reptilia | Snake | Erythrolamprus cobella | Roll et al., 2017 |
| Reptilia | Snake | Erythrolamprus dorsocorallinus | Roll et al., 2017 |
| Reptilia | Snake | Erythrolamprus epinephalus | Roll et al., 2017 |
| Reptilia | Snake | Erythrolamprus festae | Roll et al., 2017 |
| Reptilia | Snake | Erythrolamprus guentheri | Roll et al., 2017 |
| Reptilia | Snake | Erythrolamprus ingeri | Roll et al., 2017 |
| Reptilia | Snake | Erythrolamprus jaegeri | Roll et al., 2017 |
| Reptilia | Snake | Erythrolamprus janaleeae | Roll et al., 2017 |
| Reptilia | Snake | Erythrolamprus maryellenae | Roll et al., 2017 |
| Reptilia | Snake | Erythrolamprus melanotus | Roll et al., 2017 |
| Reptilia | Snake | Erythrolamprus miliaris | Roll et al., 2017 |
| Reptilia | Snake | Erythrolamprus mimus | Roll et al., 2017 |
| Reptilia | Snake | Erythrolamprus oligolepis | Roll et al., 2017 |
| Reptilia | Snake | Erythrolamprus poecilogyrus | Roll et al., 2017 |
| Reptilia | Snake | Erythrolamprus problematicus | Roll et al., 2017 |
| Reptilia | Snake | Erythrolamprus pygmaeus | Roll et al., 2017 |
| Reptilia | Snake | Erythrolamprus reginae | Roll et al., 2017; Present study |
| Reptilia | Snake | Erythrolamprus sagittifer | Roll et al., 2017 |
| Reptilia | Snake | Erythrolamprus taeniogaster | Roll et al., 2017; Present study |
| Reptilia | Snake | Erythrolamprus taeniurus | Roll et al., 2017 |
| Reptilia | Snake | Erythrolamprus torrenicola | Roll et al., 2017 |
| Reptilia | Snake | Erythrolamprus typhlus | Roll et al., 2017; Present study |
| Reptilia | Snake | Erythrolamprus zweifeli | Roll et al., 2017 |
| Reptilia | Snake | Eunectes beniensis | Roll et al., 2017 |
| Reptilia | Snake | Eunectes deschauenseei | Roll et al., 2017 |
| Reptilia | Snake | Eunectes murinus | Roll et al., 2017; Present study |
| Reptilia | Snake | Eunectes notaeus | Roll et al., 2017 |
| Reptilia | Snake | Eutrachelophis bassleri | Roll et al., 2017 |
| Reptilia | Snake | Eutrachelophis steinbachi | Roll et al., 2017 |
| Reptilia | Snake | Helicops angulatus | Roll et al., 2017; Present study |
| Reptilia | Snake | Helicops apiaka | Roll et al., 2017 |
| Reptilia | Snake | Helicops hagmanni | Roll et al., 2017 |
| Reptilia | Snake | Helicops leopardinus | Roll et al., 2017 |
| Reptilia | Snake | Helicops pastazae | Roll et al., 2017 |
| Reptilia | Snake | Helicops petersi | Roll et al., 2017 |
| Reptilia | Snake | Helicops polylepis | Roll et al., 2017; Present study |
| Reptilia | Snake | Helicops tapajonicus | Roll et al., 2017 |
| Reptilia | Snake | Helicops trivittatus | Roll et al., 2017 |
| Reptilia | Snake | Helicops yacu | Roll et al., 2017 |
| Reptilia | Snake | Hydrodynastes bicinctus | Roll et al., 2017 |
| Reptilia | Snake | Hydrodynastes gigas | Roll et al., 2017 |
| Reptilia | Snake | Hydrops martii | Roll et al., 2017 |
| Reptilia | Snake | Hydrops triangularis | Roll et al., 2017 |
| Reptilia | Snake | Imantodes cenchoa | Roll et al., 2017; Present study |
| Reptilia | Snake | Imantodes inornatus | Roll et al., 2017 |
| Reptilia | Snake | Imantodes lentiferus | Roll et al., 2017; Present study |
| Reptilia | Snake | Lachesis muta | Roll et al., 2017; Present study |
| Reptilia | Snake | Lampropeltis micropholis | Roll et al., 2017 |
| Reptilia | Snake | Leptodeira annulata | Roll et al., 2017; Present study |
| Reptilia | Snake | Leptodeira septentrionalis | Roll et al., 2017 |
| Reptilia | Snake | Leptophis ahaetulla | Roll et al., 2017 |
| Reptilia | Snake | Leptophis cupreus | Roll et al., 2017 |
| Reptilia | Snake | Leptophis riveti | Roll et al., 2017 |
| Reptilia | Snake | Liotyphlops beui | Roll et al., 2017 |
| Reptilia | Snake | Liotyphlops haadi | Roll et al., 2017 |
| Reptilia | Snake | Liotyphlops ternetzii | Roll et al., 2017 |
| Reptilia | Snake | Lygophis dilepis | Roll et al., 2017 |
| Reptilia | Snake | Lygophis lineatus | Roll et al., 2017 |
| Reptilia | Snake | Lygophis meridionalis | Roll et al., 2017 |
| Reptilia | Snake | Lygophis paucidens | Roll et al., 2017 |
| Reptilia | Snake | Mastigodryas boddaerti | Roll et al., 2017 |
| Reptilia | Snake | Mastigodryas heathii | Roll et al., 2017 |
| Reptilia | Snake | Mastigodryas moratoi | Roll et al., 2017 |
| Reptilia | Snake | Mastigodryas pleii | Roll et al., 2017 |
| Reptilia | Snake | Micrurus albicinctus | Roll et al., 2017 |
| Reptilia | Snake | Micrurus ancoralis | Roll et al., 2017 |
| Reptilia | Snake | Micrurus annellatus | Roll et al., 2017 |
| Reptilia | Snake | Micrurus averyi | Roll et al., 2017 |
| Reptilia | Snake | Micrurus bocourti | Roll et al., 2017 |
| Reptilia | Snake | Micrurus boicora | Roll et al., 2017 |
| Reptilia | Snake | Micrurus catamayensis | Roll et al., 2017 |
| Reptilia | Snake | Micrurus collaris | Roll et al., 2017 |
| Reptilia | Snake | Micrurus corallinus | Roll et al., 2017 |
| Reptilia | Snake | Micrurus diana | Roll et al., 2017 |
| Reptilia | Snake | Micrurus dissoleucus | Roll et al., 2017 |
| Reptilia | Snake | Micrurus diutius | Roll et al., 2017 |
| Reptilia | Snake | Micrurus filiformis | Roll et al., 2017 |
| Reptilia | Snake | Micrurus frontalis | Roll et al., 2017 |
| Reptilia | Snake | Micrurus hemprichii | Roll et al., 2017; Present study |
| Reptilia | Snake | Micrurus isozonus | Roll et al., 2017 |
| Reptilia | Snake | Micrurus langsdorffi | Roll et al., 2017 |
| Reptilia | Snake | Micrurus lemniscatus | Roll et al., 2017; Present study |
| Reptilia | Snake | Micrurus margaritiferus | Roll et al., 2017 |
| Reptilia | Snake | Micrurus mertensi | Roll et al., 2017 |
| Reptilia | Snake | Micrurus mipartitus | Roll et al., 2017 |
| Reptilia | Snake | Micrurus narduccii | Roll et al., 2017 |
| Reptilia | Snake | Micrurus nattereri | Roll et al., 2017 |
| Reptilia | Snake | Micrurus obscurus | Roll et al., 2017 |
| Reptilia | Snake | Micrurus ornatissimus | Roll et al., 2017 |
| Reptilia | Snake | Micrurus paraensis | Roll et al., 2017 |
| Reptilia | Snake | Micrurus peruvianus | Roll et al., 2017 |
| Reptilia | Snake | Micrurus petersi | Roll et al., 2017 |
| Reptilia | Snake | Micrurus psyches | Roll et al., 2017 |
| Reptilia | Snake | Micrurus putumayensis | Roll et al., 2017 |
| Reptilia | Snake | Micrurus pyrrhocryptus | Roll et al., 2017 |
| Reptilia | Snake | Micrurus remotus | Roll et al., 2017 |
| Reptilia | Snake | Micrurus scutiventris | Roll et al., 2017 |
| Reptilia | Snake | Micrurus serranus | Roll et al., 2017 |
| Reptilia | Snake | Micrurus spixii | Roll et al., 2017 |
| Reptilia | Snake | Micrurus steindachneri | Roll et al., 2017 |
| Reptilia | Snake | Micrurus surinamensis | Roll et al., 2017; Present study |
| Reptilia | Snake | Micrurus tikuna | Roll et al., 2017 |
| Reptilia | Snake | Micrurus tschudii | Roll et al., 2017 |
| Reptilia | Snake | Ninia atrata | Roll et al., 2017 |
| Reptilia | Snake | Ninia hudsoni | Roll et al., 2017 |
| Reptilia | Snake | Ninia teresitae | Roll et al., 2017 |
| Reptilia | Snake | Oxybelis aeneus | Roll et al., 2017; Present study |
| Reptilia | Snake | Oxybelis fulgidus | Roll et al., 2017 |
| Reptilia | Snake | Oxyrhopus erdisii | Roll et al., 2017 |
| Reptilia | Snake | Oxyrhopus fitzingeri | Roll et al., 2017 |
| Reptilia | Snake | Oxyrhopus formosus | Roll et al., 2017; Present study |
| Reptilia | Snake | Oxyrhopus guibei | Roll et al., 2017 |
| Reptilia | Snake | Oxyrhopus leucomelas | Roll et al., 2017 |
| Reptilia | Snake | Oxyrhopus marcapatae | Roll et al., 2017 |
| Reptilia | Snake | Oxyrhopus melanogenys | Roll et al., 2017; Present study |
| Reptilia | Snake | Oxyrhopus occipitalis | Roll et al., 2017 |
| Reptilia | Snake | Oxyrhopus petolarius | Roll et al., 2017; Present study |
| Reptilia | Snake | Oxyrhopus rhombifer | Roll et al., 2017 |
| Reptilia | Snake | Oxyrhopus trigeminus | Roll et al., 2017 |
| Reptilia | Snake | Oxyrhopus vanidicus | Roll et al., 2017; Present study |
| Reptilia | Snake | Palusophis bifossatus | Roll et al., 2017 |
| Reptilia | Snake | Phalotris nasutus | Roll et al., 2017 |
| Reptilia | Snake | Phalotris sansebastiani | Roll et al., 2017 |
| Reptilia | Snake | Phalotris tricolor | Roll et al., 2017 |
| Reptilia | Snake | Philodryas aestiva | Roll et al., 2017 |
| Reptilia | Snake | Philodryas agassizii | Roll et al., 2017 |
| Reptilia | Snake | Philodryas amaru | Roll et al., 2017 |
| Reptilia | Snake | Philodryas argentea | Roll et al., 2017; Present study |
| Reptilia | Snake | Philodryas baroni | Roll et al., 2017 |
| Reptilia | Snake | Philodryas boliviana | Roll et al., 2017 |
| Reptilia | Snake | Philodryas erlandi | Roll et al., 2017 |
| Reptilia | Snake | Philodryas georgeboulengeri | Roll et al., 2017 |
| Reptilia | Snake | Philodryas laticeps | Roll et al., 2017 |
| Reptilia | Snake | Philodryas nattereri | Roll et al., 2017 |
| Reptilia | Snake | Philodryas olfersii | Roll et al., 2017 |
| Reptilia | Snake | Philodryas patagoniensis | Roll et al., 2017 |
| Reptilia | Snake | Philodryas psammophidea | Roll et al., 2017 |
| Reptilia | Snake | Philodryas simonsii | Roll et al., 2017 |
| Reptilia | Snake | Philodryas tachymenoides | Roll et al., 2017 |
| Reptilia | Snake | Philodryas varia | Roll et al., 2017 |
| Reptilia | Snake | Philodryas viridissima | Roll et al., 2017 |
| Reptilia | Snake | Phimophis guerini | Roll et al., 2017 |
| Reptilia | Snake | Phimophis guianensis | Roll et al., 2017 |
| Reptilia | Snake | Phrynonax poecilonotus | Roll et al., 2017 |
| Reptilia | Snake | Phrynonax polylepis | Roll et al., 2017 |
| Reptilia | Snake | Pliocercus euryzonus | Roll et al., 2017 |
| Reptilia | Snake | Pseudalsophis elegans | Roll et al., 2017 |
| Reptilia | Snake | Pseudoboa coronata | Roll et al., 2017; Present study |
| Reptilia | Snake | Pseudoboa martinsi | Roll et al., 2017 |
| Reptilia | Snake | Pseudoboa neuwiedii | Roll et al., 2017 |
| Reptilia | Snake | Pseudoboa nigra | Roll et al., 2017 |
| Reptilia | Snake | Pseudoeryx plicatilis | Roll et al., 2017 |
| Reptilia | Snake | Psomophis genimaculatus | Roll et al., 2017 |
| Reptilia | Snake | Psomophis joberti | Roll et al., 2017 |
| Reptilia | Snake | Rhachidelus brazili | Roll et al., 2017 |
| Reptilia | Snake | Rhinobothryum lentiginosum | Roll et al., 2017; Present study |
| Reptilia | Snake | Saphenophis boursieri | Roll et al., 2017 |
| Reptilia | Snake | Siagonodon cupinensis | Roll et al., 2017 |
| Reptilia | Snake | Siagonodon septemstriatus | Roll et al., 2017 |
| Reptilia | Snake | Sibon nebulatus | Roll et al., 2017 |
| Reptilia | Snake | Siphlophis ayauma | Roll et al., 2017 |
| Reptilia | Snake | Siphlophis cervinus | Roll et al., 2017; Present study |
| Reptilia | Snake | Siphlophis compressus | Roll et al., 2017; Present study |
| Reptilia | Snake | Siphlophis worontzowi | Roll et al., 2017 |
| Reptilia | Snake | Spilotes pullatus | Roll et al., 2017; Present study |
| Reptilia | Snake | Spilotes sulphureus | Roll et al., 2017 |
| Reptilia | Snake | Synophis bicolor | Roll et al., 2017 |
| Reptilia | Snake | Synophis bogerti | Roll et al., 2017 |
| Reptilia | Snake | Synophis insulomontanus | Roll et al., 2017 |
| Reptilia | Snake | Synophis lasallei | Roll et al., 2017 |
| Reptilia | Snake | Synophis zamora | Roll et al., 2017 |
| Reptilia | Snake | Tachymenis affinis | Roll et al., 2017 |
| Reptilia | Snake | Tachymenis attenuata | Roll et al., 2017 |
| Reptilia | Snake | Tachymenis peruviana | Roll et al., 2017 |
| Reptilia | Snake | Tachymenis tarmensis | Roll et al., 2017 |
| Reptilia | Snake | Taeniophallus brevirostris | Roll et al., 2017; Present study |
| Reptilia | Snake | Taeniophallus nicagus | Roll et al., 2017 |
| Reptilia | Snake | Taeniophallus occipitalis | Roll et al., 2017 |
| Reptilia | Snake | Taeniophallus quadriocellatus | Roll et al., 2017 |
| Reptilia | Snake | Tantilla capistrata | Roll et al., 2017 |
| Reptilia | Snake | Tantilla melanocephala | Roll et al., 2017 |
| Reptilia | Snake | Tantilla tjiasmantoi | Roll et al., 2017 |
| Reptilia | Snake | Thamnodynastes chaquensis | Roll et al., 2017 |
| Reptilia | Snake | Thamnodynastes hypoconia | Roll et al., 2017 |
| Reptilia | Snake | Thamnodynastes lanei | Roll et al., 2017 |
| Reptilia | Snake | Thamnodynastes pallidus | Roll et al., 2017 |
| Reptilia | Snake | Thamnodynastes ramonriveroi | Roll et al., 2017 |
| Reptilia | Snake | Tomodon orestes | Roll et al., 2017 |
| Reptilia | Snake | Trilepida anthracina | Roll et al., 2017 |
| Reptilia | Snake | Trilepida brasiliensis | Roll et al., 2017 |
| Reptilia | Snake | Trilepida brevissima | Roll et al., 2017 |
| Reptilia | Snake | Trilepida dimidiata | Roll et al., 2017 |
| Reptilia | Snake | Trilepida macrolepis | Roll et al., 2017 |
| Reptilia | Snake | Tropidophis taczanowskyi | Roll et al., 2017 |
| Reptilia | Snake | Typhlophis squamosus | Roll et al., 2017 |
| Reptilia | Snake | Xenodon matogrossensis | Roll et al., 2017 |
| Reptilia | Snake | Xenodon merremii | Roll et al., 2017 |
| Reptilia | Snake | Xenodon nattereri | Roll et al., 2017 |
| Reptilia | Snake | Xenodon rabdocephalus | Roll et al., 2017 |
| Reptilia | Snake | Xenodon severus | Roll et al., 2017; Present study |
| Reptilia | Snake | Xenodon werneri | Roll et al., 2017 |
| Reptilia | Snake | Xenopholis scalaris | Roll et al., 2017 |
| Reptilia | Snake | Xenopholis undulatus | Roll et al., 2017 |
| Reptilia | Snake | Xenopholis werdingorum | Roll et al., 2017 |
| Reptilia | Testudines | Acanthochelys macrocephala | Rhodin et al., 2021 |
| Reptilia | Testudines | Chelonoidis carbonarius | Rhodin et al., 2021 |
| Reptilia | Testudines | Chelonoidis denticulatus | Rhodin et al., 2021; Present study |
| Reptilia | Testudines | Chelus fimbriata | Rhodin et al., 2021; Present study |
| Reptilia | Testudines | Chelus orinocensis | Rhodin et al., 2021 |
| Reptilia | Testudines | Kinosternon scorpioides | Rhodin et al., 2021; Present study |
| Reptilia | Testudines | Mesoclemmys gibba | Rhodin et al., 2021 |
| Reptilia | Testudines | Mesoclemmys nasuta | Rhodin et al., 2021 |
| Reptilia | Testudines | Mesoclemmys raniceps | Rhodin et al., 2021 |
| Reptilia | Testudines | Mesoclemmys vanderhaegei | Rhodin et al., 2021 |
| Reptilia | Testudines | Mesoclemmys wermuthi | Rhodin et al., 2021 |
| Reptilia | Testudines | Peltocephalus dumerilianus | Rhodin et al., 2021 |
| Reptilia | Testudines | Phrynops geoffroanus | Rhodin et al., 2021 |
| Reptilia | Testudines | Phrynops tuberosus | Rhodin et al., 2021 |
| Reptilia | Testudines | Platemys platycephala melanonota | Rhodin et al., 2021 |
| Reptilia | Testudines | Platemys platycephala platycephala | Rhodin et al., 2021; Present study |
| Reptilia | Testudines | Podocnemis erythrocephala | Rhodin et al., 2021 |
| Reptilia | Testudines | Podocnemis expansa | Rhodin et al., 2021; Present study |
| Reptilia | Testudines | Podocnemis sextuberculata | Rhodin et al., 2021; Present study |
| Reptilia | Testudines | Podocnemis unifilis | Rhodin et al., 2021 |
| Reptilia | Testudines | Podocnemis vogli | Rhodin et al., 2021 |
| Reptilia | Testudines | Rhinemys rufipes | Rhodin et al., 2021 |
| Reptilia | Testudines | Rhinoclemmys punctularia flammigera | Rhodin et al., 2021 |
| Reptilia | Testudines | Rhinoclemmys punctularia punctularia | Rhodin et al., 2021 |

REFERENCES 

IUCN (2024) The IUCN Red List of Threatened Species. Version 2023-1. [www.iucnredlist.org](http://www.iucnredlist.org)

Rhodin AGJ, Iverson JB, Bour R et al (2021) Turtles of the World: Annotated Checklist and Atlas of Taxonomy, Synonymy, Distribution, and Conservation Status (9th Ed.). Chelonian Res Monogr 8: 1 – 472. [https://doi.org/10.3854/crm.8.checklist.atlas.v9.2021](https://doi.org/10.3854/crm.8.checklist.atlas.v9.2021)

Roll U, Feldman A, Novosolov M et al (2017) The global distribution of tetrapods reveals a need for targeted reptile conservation. Nat. Ecol. Evol, 1(11), 1677-1682. https://doi.org/10.1038/s41559-017-0332-2

 

# Table S1.2: ANOVA and Tukey HSD test comparing diversity metrics of amphibian lineages across ecoregions in the Amazon Basin.

|  |  | Anura | Caudata | Gymnophiona |
| --- | --- | --- | --- | --- |
| Metric | Bioreg | mean | sd | Tukey | mean | sd | Tukey | mean | sd | Tukey |
| PD | Andes | 1261.409 | 961.546 | a | 0.832 | 4.96 | a | 87.066 | 126.756 | a |
| Western Lowlands | 4738.695 | 1158.221 | b | 3.24 | 8.19 | b | 362.776 | 110.198 | b |
| Eastern Lowlands | 4795.836 | 555.665 | c | 1.42 | 1.65 | c | 436.337 | 19.981 | c |
| Guiana Shield | 3967.548 | 526.354 | d | 0.000 | 0.041 | d | 376.055 | 73.489 | d |
| Brazilian Shield | 3680.764 | 847.012 | e | 0.531 | 1.32 | e | 326.515 | 121.984 | e |
|  |  | F = 17603.291; p = 0.001 | F = 857.4; p = 0.001 | F = 10634.773; p = 0.001 |
| Metric | Bioreg | mean | sd | Tukey | mean | sd | Tukey | mean | sd | Tukey |
| PDmntd | Andes | 154.672 | 36.988 | a | 3.53 | 9.62 | a | 23.565 | 64.460 | a |
| Western Lowlands | 103.991 | 9.287 | b | 17.3 | 13.6 | b | 131.181 | 70.757 | a |
| Eastern Lowlands | 101.905 | 2.767 | c | 10.7 | 12.4 | c | 158.836 | 5.496 | b |
| Guiana Shield | 106.863 | 4.327 | d | 0.000 | 0.306 | d | 159.258 | 46.994 | c |
| Brazilian Shield | 108.306 | 6.648 | e | 3.99 | 9.18 | e | 156.146 | 57.320 | d |
|  | F = 15845.078; p = 0.001 | F = 4328; p = 0.001 | F = 6688.765; p = 0.001 |
| Metric | Bioreg | mean | sd | Tukey | mean | sd | Tukey | mean | sd | Tukey |
| FD | Andes | 4.514 | 3.821 | a | 0.022 | 0.060 | a | 0.194 | 0.284 | a |
| Western Lowlands | 21.193 | 5.401 | b | 0.108 | 0.086 | b | 0.955 | 0.343 | b |
| Eastern Lowlands | 22.434 | 2.588 | c | 0.066 | 0.076 | c | 1.081 | 0.058 | c |
| Guiana Shield | 18.525 | 2.552 | d | 0.000 | 0.002 | c | 1.000 | 0.206 | d |
| Brazilian Shield | 17.786 | 4.170 | e | 0.025 | 0.056 | d | 0.810 | 0.318 | e |
|  |  | F = 19059.922; p = 0.001 | F =4311.303; p = 0.001 | F = 9966.030; p = 0.001 |
| Metric | Bioreg | mean | sd | Tukey | mean | sd | Tukey | mean | sd | Tukey |
| FDmntd | Andes | 0.557 | 0.135 | a | 0.006 | 0.036 | a | 0.065 | 0.180 | a |
| Western Lowlands | 0.446 | 0.041 | b | 0.022 | 0.055 | b | 0.379 | 0.239 | b |
| Eastern Lowlands | 0.456 | 0.021 | c | 0.007 | 0.009 | b | 0.318 | 0.112 | c |
| Guiana Shield | 0.496 | 0.028 | d | 0.000 | 0.000 | c | 0.504 | 0.212 | d |
| Brazilian Shield | 0.493 | 0.025 | e | 0.003 | 0.006 | d | 0.237 | 0.182 | e |
|  |  | F = 5044.955; p = 0.001 | F = 945.1; p = 0.001 | F = 4862.731; p = 0.001 |
| Metric | Bioreg | mean | sd | Tukey | mean | sd | Tukey | mean | sd | Tukey |
| TD | Andes | 15.704 | 15.458 | a | 0.142 | 0.392 | a | 0.541 | 0.845 | a |
| Western Lowlands | 82.637 | 24.359 | b | 0.711 | 0.598 | b | 3.067 | 1.360 | b |
| Eastern Lowlands | 84.623 | 12.252 | c | 0.425 | 0.494 | c | 3.932 | 0.376 | b |
| Guiana Shield | 65.555 | 10.750 | d | 0.000 | 0.012 | c | 3.050 | 0.854 | c |
| Brazilian Shield | 63.616 | 16.616 | e | 0.159 | 0.366 | d | 3.000 | 1.133 | d |
|  |  | F = 16269.347; p = 0.001 | F = 4198.915; p = 0.001 | F = 9248.154; p = 0.001 |

# Table S1.3: ANOVA and Tukey HSD test comparing diversity metrics of reptile lineages across ecoregions in the Amazon Basin.

|  |  | Crocodilians | Lizard | Snake | Testudines |
| --- | --- | --- | --- | --- | --- |
| Metric | Ecoregion | mean | sd | Tukey | mean | sd | Tukey | mean | sd | Tukey | mean | sd | Tukey |
| PD | Andes | 0.687 | 0.702 | a | 1147.320 | 550.161 | a | 1048.657 | 341.569 | a | 0.604 | 1.452 | a |
| Western Lowlands | 1.649 | 0.194 | a | 2332.832 | 270.677 | a | 1670.093 | 122.604 | b | 5.921 | 1.236 | b |
| Eastern Lowlands | 1.649 | 0.072 | a | 2506.767 | 107.643 | b | 1839.352 | 62.923 | c | 6.519 | 1.005 | c |
| Guiana Shield | 1.655 | 0.180 | b | 2515.068 | 139.054 | b | 1927.647 | 59.074 | d | 4.899 | 2.088 | d |
| Brazilian Shield | 1.565 | 0.182 | c | 2325.372 | 313.458 | c | 1811.974 | 134.925 | e | 4.242 | 1.895 | e |
|  |  | F = 13460.916; p = 0.001 | F = 21964.933; p = 0.001 | F = 31947.313; p = 0.001 | F = 13730.047; p = 0.001 |
| Metric | Ecoregion | mean | sd | Tukey | mean | sd | Tukey | mean | sd | Tukey | mean | sd | Tukey |
| PDmntd | Andes | 0.214 | 0.401 | a | 126.886 | 55.268 | a | 33.016 | 8.830 | a | 0.175 | 0.468 | a |
| Western Lowlands | 0.567 | 0.135 | b | 71.992 | 6.451 | b | 23.965 | 1.013 | b | 0.811 | 0.194 | b |
| Eastern Lowlands | 0.538 | 0.056 | b | 68.753 | 3.108 | b | 22.808 | 0.350 | c | 0.741 | 0.109 | c |
| Guiana Shield | 0.567 | 0.128 | c | 69.496 | 3.488 | c | 22.947 | 0.306 | d | 0.837 | 0.402 | d |
| Brazilian Shield | 0.591 | 0.224 | d | 71.649 | 5.539 | c | 23.190 | 0.757 | e | 1.047 | 0.463 | e |
|  |  | F = 3753.033; p = 0.001 | F = 10780.958; p = 0.001 | F = 13095.855; p = 0.001 | F = 6065.743; p = 0.001 |
| Metric | Ecoregion | mean | sd | Tukey | mean | sd | Tukey | mean | sd | Tukey | mean | sd | Tukey |
| FD | Andes | 0.237 | 0.259 | a | 3.073 | 1.519 | a | 10.535 | 3.849 | a | 0.204 | 0.490 | a |
| Western Lowlands | 0.716 | 0.100 | b | 6.654 | 0.780 | b | 17.538 | 1.438 | b | 1.973 | 0.428 | b |
| Eastern Lowlands | 0.737 | 0.046 | b | 7.409 | 0.449 | c | 19.012 | 0.565 | b | 2.315 | 0.343 | c |
| Guiana Shield | 0.717 | 0.088 | c | 7.718 | 0.583 | d | 18.777 | 0.489 | c | 1.654 | 0.691 | d |
| Brazilian Shield | 0.675 | 0.137 | d | 6.998 | 0.733 | e | 18.801 | 1.129 | d | 1.352 | 0.644 | e |
|  |  | F = 15430.124; p = 0.001 | F = 31456.056; p = 0.001 | F = 30433.697; p = 0.001 | F = 15144.876; p = 0.001 |
| Metric | Ecoregion | mean | sd | Tukey | mean | sd | Tukey | mean | sd | Tukey | mean | sd | Tukey |
| FDmntd | Andes | 0.077 | 0.148 | a | 0.385 | 0.137 | a | 0.391 | 0.085 | a | 0.066 | 0.163 | a |
| Western Lowlands | 0.260 | 0.029 | a | 0.257 | 0.035 | b | 0.289 | 0.017 | b | 0.392 | 0.081 | b |
| Eastern Lowlands | 0.265 | 0.012 | b | 0.268 | 0.040 | c | 0.263 | 0.011 | c | 0.366 | 0.064 | c |
| Guiana Shield | 0.263 | 0.031 | c | 0.305 | 0.023 | d | 0.262 | 0.013 | d | 0.350 | 0.134 | d |
| Brazilian Shield | 0.243 | 0.054 | d | 0.276 | 0.025 | e | 0.279 | 0.019 | d | 0.379 | 0.134 | e |
|  |  | F = 10544.875; p = 0.001 | F = 5968.327; p = 0.001 | F = 16928.318; p = 0.001 | F = 8435.637; p = 0.001 |
| Metric | Ecoregion | mean | sd | Tukey | mean | sd | Tukey | mean | sd | Tukey | mean | sd | Tukey |
| TD | Andes | 0.966 | 1.199 | a | 14.412 | 9.277 | a | 45.279 | 20.424 | a | 0.783 | 1.946 | a |
| Western Lowlands | 3.843 | 0.571 | b | 38.932 | 6.457 | b | 87.727 | 9.356 | b | 8.290 | 2.047 | b |
| Eastern Lowlands | 3.985 | 0.291 | b | 42.419 | 4.294 | c | 101.698 | 3.996 | c | 10.511 | 2.373 | c |
| Guiana Shield | 3.832 | 0.497 | c | 40.730 | 4.633 | d | 100.349 | 4.536 | d | 7.212 | 3.533 | d |
| Brazilian Shield | 3.665 | 0.922 | d | 38.624 | 5.544 | e | 97.139 | 8.337 | e | 5.613 | 3.183 | e |
|  |  | F = 17147.817; p = 0.001 | F = 21851.610; p = 0.001 | F = 35592.062; p = 0.001 | F = 12333.188; p = 0.001 |

# Figure S1.1: Additional sampling points located in the Amazon Basin. Sites: 1) Reserva Nacional Pacaya Samiria; 2) Centro de Investigación y Enseñanza Forestal (CIEFOR); 3) Reserva Forestal Santa Cruz; 4) Reserva Nacional Allpahuayo Mishana; 5) Iquitos; 6) Project Amazon; 7) Requena; 8) Catuaba; 9) Balbina; 10) Presidente Figueredo; 11) Segunda Jerusalén; 12) Aguas Calientes – Huacharos; 13) Inkaterra; 14) Juninjillo; 15) Mâncio Lima; 16) Mashira; 17) Puerto Elvira; 18) Soledad; 19) Salvador Pava; 20) Pintoyacu; 21) Alto Nanay; 22) Cuenca Rio Itaya; 23) Tamshiyacu; 24) Situche Norte 25) Bretaña; 26) Machinguenga; 27) Chambira.

# Figure S1.2: Comparison of Olson et al. (2001) Ecoregion Classification (a) with the Present Study's Reclassified Ecoregions (b) in the Amazon Basin.
